# Nrf1 acts as a highly-conserved determinon for maintaining robust redox homeostasis in the eco-evo-devo process of life histories

**DOI:** 10.15698/cst2025.07.306

**Published:** 2025-07-07

**Authors:** Yiguo Zhang, Xi Chen, Meng Wang, Yuping Zhu, Wei Shi, Chao Li, Zhengwen Zhang, Hiroaki Taniguchi, Ping Ao

**Affiliations:** 1 The Laboratory of Cell Biochemistry and Topogenetic Regulation, College of Bioengineering and Faculty of Medical Sciences, Chongqing University, No. 174 Shazheng Street, Shapingba District, Chongqing 400044, China.; 2 School of Life and Health Sciences, Fuyao University of Science and Technology, No. 104 Wisdom Avenue, Nanyu Town, Minhou County High-Tech District, Fuzhou 350109, Fujian, China.; 3 School of Basic Medicine, Guizhou Medical University, No. 6 Ankang Avenue, GUI ‘an New District, Guizhou 561113, China.; 4 State Key Laboratory of Oil and Gas Reservoir Geology and Exploitation & Institute of Sedimentary Geology, Chengdu University of Technology, Chengdu 610059, China.; 5 Key Laboratory of Deep-time Geography and Environment Reconstruction and Applications of Ministry of Natural Resources, Chengdu University of Technology, Chengdu 610059, China.; 6 International Center for Sedimentary Geochemistry and Biogeochemistry Research, Chengdu University of Technology, Chengdu 610059, China.; 7 Laboratory of Neuroscience, Institute of Cognitive Neuroscience and School of Pharmacy, University College London, 29-39 Brunswick Square, London WC1N 1AX, England, United Kingdom.; 8 Department of Experimental Embryology, Institute of Genetics and Animal Biotechnology, Polish Academy of Sciences, 05-552 Jastrzebiec, Poland.; 9 African Genome Center, Mohammed VI Polytechnic University (UM6P), Ben Guerir 43150, Morocco.; 10 College of Biomedical Engineering, Sichuan University, Chengdu, Sichuan 610044, China.

**Keywords:** Nrf1, Nrf2, homeostasis, determinon, regulon, topogenetics, redox stress, eco-evo-devo, redox code, stress-coping code, oncoprotist, ‘zero theory’, Reverse Central Dogma, Grand Redox-Unifying Theory (GRUT), and murburn concept

## Abstract

Differential and even opposing functions of two major antioxidant transcription factors Nrf1 and Nrf2 (encoded by *Nfe2l1* and* Nfe2l2*, respectively) are determined by distinctions in their tempospatial positioning, topological repartitioning, proteolytic processing, and biochemical modification, as well as in their shared evolutionary origin. As a matter of fact, the allelopathic potentials of Nrf1 and Nrf2 (both resembling two entangled ‘Yin-Yang’ quanta that comply with a dialectic law of the unity of opposites) are fulfilled to coordinately control redox physiological homeostasis so as to be maintained within the presetting thresholds. By putative exponential curves of redox stress and intrinsic anti-redox capability, there is inferable to exist a set point at approaching zero with the ‘Golden Mean’ for the healthy survival (i.e., dubbed the ‘zero theory’). A bulk of the hitherto accumulating evidence demonstrates that the set point of redox homeostasis is dictated selectively by multi-hierarchical threshold settings, in which the living fossil-like Nrf1 acts as a robust indispensable determinon, whereas Nrf2 serves as a versatile chameleon-like master regulon, in governing the redox homeodynamic ranges. This is attributable to the facts that Nrf2 has exerted certain ‘double-edged sword’ effects on life process, whereas Nrf1 executes its essential physiobiological functions, along with unique pathophysiological phenotypes, by integrating its ‘three-in-one’ roles elicited as a specific triplet of direct sensor, transducer and effector within multi-hierarchical stress responsive signaling to redox metabolism and target gene reprogramming. Here, we also critically reviewed redox regulation of physio-pathological functions from the eco-evo-devo perspectives, through those coding rules (redox code, stress-coping code, and topogenetic code). The evolving concepts on stress and redox stress were also further revisited by scientific principles of physics and chemistry. Besides, several novel concepts such as oncoprotists, Reverse Central Dogma, and Grand Redox-Unifying Theory’ (GRUT) of life, together with diffusive reactive species (DRS)-based murburn concept integrating all stochastic electron-, proton- and/or moiety-transfer reactive and interactive processes (e.g., PCHEMS), are introduced in this interdisciplinary and synthetic review.

## Abbreviations

ARE - antioxidant response element

BAT - brown adipose tissue

Cat - catalase

CHR - cell homeostatic response

CNC-bZIP - cap 'n' Collar and basic region leucine zipper

CSR - cellular stress response

DEG - differentially expressed gene

DRS - diffusive reactive species

ECM - extracellular matrix

EMT - epithelial mesenchymal transition

EpRE - electrophile response element

ER - endoplasmic reticulum

ERAD - ER-associated protein degradation

ESC - embryonic stem cell

Frx - ferredoxin

GAS - general-adaptation syndrome

GH - growth hormone

GOE - great oxidation event

GRUT - Grand Redox-Unifying Theory

Grx - glutaredoxin

HDL - high-density lipoprotein

HGT - horizontal gene transfer

IRC - intrinsic reactive compounds

KO - knockout

LUCA - last universal common ancestor

LXR - liver X receptor

MI - myocardial infarction

NASH - non-alcoholic steatohepatitis

NESR - neuroendocrine stress response

NOE - Neoproterozoic oxygenation event

NST - N-glycosylated transactivation

NTD - N-terminal domain

OHR - organismal homeostatic response

OSR - organismal stress response

Prx - peroredoxin

RNS - reactive nitrogen species

ROS - reactive oxygen species

RSS - reactive sulfur species

RXR - retinoid X receptor

SOD - superoxide dismutase

TAD - transactivation domain

TM - transmembrane

Trx - thioredoxin

TU - tunicamycin

UBL - ubiquitin-like domain

UPR - unfolded protein response

UPS - ubiquitin-proteasome system

USP - ubiquitin-specific proteases

VLDL - very low-density lipoprotein

WAT - white adipose tissue.

## INTRODUCTION

**Figure 1:**
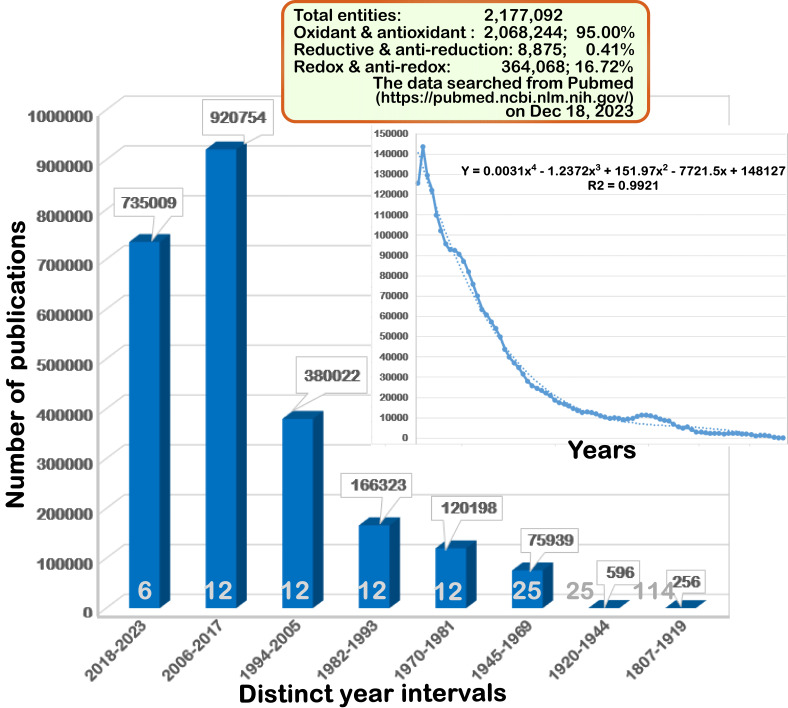
FIGURE 1: An exponentially increasing tendency of publications in the redox-relevant fields.

By searching the PubMed-cited literature on the *red*uctive and *ox*idative (redox called collectively, but dictated by gain or loss of electrons and/or hydrogens, respectively) and anti-redox (antioxidant and anti-reduction), it is absolutely amazing to find that an overwhelmingly large number of publications by such relevant conceptual terms had been collected within at least 2,177,092 entries of this library (at https://pubmed.ncbi.nlm.nih.gov), until the 18^th^ of Dec, 2023, heretofore. As shown in **Figure 1**, this is placed in an exponentially expanding field, with greatly broad implications in physiology, pathophysiology, biomedical and life sciences, except for early crawling over a century after the first experimental report in 1807 by Dispan 1 on the gaseous oxide of azote and the second one in 1811 by Wollaston on cystic oxide, a new species of urinary calculus 2. Nonetheless, a great majority of redox studies have been focused disproportionately (95%) on those oxidative and antioxidant topics, whereas the other studies on reductive and anti-reduction topics were done only by a ratio of lesser than 0.5 % (**Figure 1**, top box). The distinction demonstrates that (anti)-reductive researches had been roughly neglected or ignored for a rather long term. Rather recently, the crucial importance of (anti-)reductive studies appears to be gradually recognized in this redox research field 3,4,5,6,7,8, but still needs to be further imposed.

The concept of oxidative stress was firstly proposed by Paniker *et al.* in 1970 9 and later formulated by Helmut Sies in 1985, and further redefined by Dean P. Jones in 2006 10,11,12. Albeit such a continuously-heating topic on redox stress (including reductive stress 13) and redox defense response seem to have thoroughly penetrated into the cell stress zeitgeist, it is remaining open to arouse great concerns from workers in distinct fields and also is one of the most persistently-existing intractable problems to be addressed for health and disease 14, particularly in a vast variety of changing environmental conditions. Among its merits elicited by evoking biological stress responses, a steady-state redox balance is constantly maintained within a homeodynamic threshold range by cell respiration, aerobic metabolism and redox switches governing redox stress defense responses 10,11. In the meantime, the pitfalls of oxidative stress could also lead to another indiscriminate use of this term as a global concept, but without a clear relation to ‘real redox chemistry’ per se, in each of particular cases. For the underlying molecular details, the major roles for antioxidant and anti-reductive defense systems are fulfilled predominantly by those redox-controlling biomolecules, such as key proteins, enzymes, transcription factors and co-factors, which are involved in cellular redox biochemical and metabolic processes, as well as by the adaptive reprogramming of their relevant gene expression profiles in the normal physiological and even pathophysiological responses to redox stress during distinct eco-evo-devo stages of life histories.

In this synthetic review, we firstly introduce the past and present of redox-based systems in the ‘redox central theory’ (that are distinctive from, but correlative to, the generally nonspecific ‘stress-coping system’), along with their pivotal roles being exerted during the course of life’s origin and ensuing evolution. Secondly, we have critically reviewed redox regulation of physio-pathological functions from the eco-evo-devo perspectives, through distinct mechanisms and obligate coding rules (e.g. redox code, stress-coping code, and topogenetic code, all selected by nature). Thirdly, the evolving concepts of stress and redox stress were further revisited by scientific principles of physics and chemistry, together with redox stress and anti-redox response being quantitatively stratified with the ‘zero theory’, in addition to two novel concepts of ‘oncoprotists’ and ‘reverse central dogma’ being proposed by a means of the interdisciplinary synthesis. Fourthly, we listed a host of convincing evidence, revealing that the antioxidant Nrf1 (nuclear factor, erythroid 2-related factor 1, encoded by* Nfe2l1*) is a living fossil’s transcription factor, that is rather closer than Nrf2, to their commonly-shared ancient orthologues arising from the eco-evo-devo process of life histories. Of sharp not, Nrf1 also exhibits its unique pathophysiological phenotypes, all of which are distinctive or absent from Nrf2 (nuclear factor, erythroid 2-related factor 2, encoded by* Nfe2l2*). This is attributable to the unique topogenetic folding of Nrf1 and dynamic topovectorial repositioning of this CNC-bZIP factor across endoplasmic reticulum (ER) membranes to dislocate the nucleus before transcriptional regulation of cognate target genes. Lastly, we have also critically reviewed the uniquely differentiated yet integrated roles of both Nrf1 and Nrf2 in governing cellular redox, energy, and metabolism homeostasis and organ integrity during distinct life processes, in which activation of Nrf1 is evoked by distinct regulatory mechanisms. Overall, the allelopathic potentials of Nrf1 and Nrf2 (both dubbed as two entangled ‘Yin-Yang’ quanta abiding by a dialectic law of the unity of opposites) are fulfilled to coordinately control redox physiological homeostasis within certain homeodynamic threshold ranges, which are being perpetually maintained for the normal healthy survival.

## THE FAR PAST AND PRESENT OF REDOX-BASED SYSTEMS

### ‘Central redox theory’ refined by the nature

From the origin of life and its ensuing evolution to the modern present of the 21^st^ century, all cellular life forms have greatly experienced a vast variety of challenges within diversely changing environments, a major one of which is spawned predominantly by cellular stress, particularly redox stress, threating normal homeostasis of a life system 15,16,17. It is since life fundamentally depends on the free energy (ΔG) provided by electrochemical disequilibrium between reduced (electron-donating) and oxidized (electron-accepting) environmental substrates 18 11 and also has a crucial ability to further convert such a redox environmental disequilibria into its intracellular disequilibria, as defined by Schrödinger 20. The redox (i.e., electron exchanging) reactions had, de facto, existed early in both the chemical and metabolic worlds during the origin of life and have been always persisted in all life processes throughout the entire evolutionary course until now 6,21,22. Of note, those reductive stressors are much likely to provide a primordial force, insomuch as to meet essential bioenergetics needs for the origin of life and its long-term evolution 18,23, and has been embodied and embedded in the present-day’s life processes 15,24. This is remaining to be accompanied by an ancient anti-reduction strategy for fitness to prevent excessive reduction of intrinsic compounds by undergoing reduction themselves or by removing of ambient dihydrogen (H_2_, which enables reduction of sulfate to H_2_S, along with certain organic materials being oxidized) 8. However, much less attentions have been paid on such anti-reductive responses to reductive stress, relative to their counterparts in an overwhelming majority of studies for the prevailing antioxidant responses to oxidative stress. 

**Figure 2:**
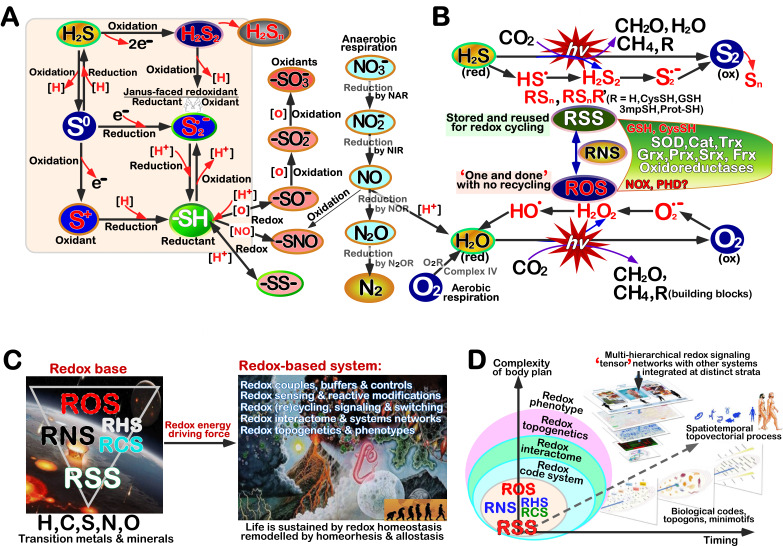
FIGURE 2: Major redox reactive species and their physiological significances in the eco-evo-devo process of life. **(A)** H_2_S and relevant reactive sulfur species (RSS), along with reactive nitrogen species (RNS), part of which are from the NO respiratory chain by a series of reductases to produce energy under anaerobic conditions 23. **(B)** The upper panel shows that H_2_S is involved in anoxygenic photosynthesis to yield energy and building blocks, and also in its successive oxidative reactions to yield RSS together with S2 and Sn (polysulfides) 37. The lower panel shows that O_2_ arises from the oxygenic photosynthesis, and is involved in the aerobic respiratory chain to yield biological energy and building blocks, aside from it being reduced to reactive oxygen species (ROS). In addition, portion of evolutionally conserved oxidoreductases were also indicated. In RSnR’, R and R’ represent CysSH, GSH, 3mpSH (3-mercaptoSH) and/or protein-SH. All other abbreviations were defined in the text and relevant references cited. **(C)** The physiological roles of major reactive species in co-evolution of life with its surrounding environments on the earth. Those reactive species arise from the ecological environmental evolution of early earth (left panel), to provide energy for redox-based systems and serve as driving forces to promote the origin of life and its evolution (right panel). **(D)** Multi-dimensional orthogonality of redox regulation by integrated ‘tensor’ networks of multi-hierarchical signaling with distinct omics systems across strata, levels and scales of biological self-organization. The complexity of life’s body plan (Y axle) dictated by its functional information is incrementing with the evolution timing of life (X axle), which complies with a law of Darwinian evolution dynamics 38. Note: a part of this panel was adapted from 39.

 A bulk of accumulating evidence has demonstrated that, the hitherto known free radicals and reactive species of sulfur (RSS), nitrogen (RNS), oxygen (ROS), carbonyl (RCS) and halogen (RHS), along with transition metals (e.g., Fe, Cu, Mn, Zn, Ni, Co, Mg) and minerals (e.g., Se, As), all of which can be collectively construed as ‘Redox X species’, serve as constantly electron- and/or proton-exchanging key players in distinct series of multi-hierarchical redox reactions, relevant interactions and/or relationships with biomolecules 25,26,27,28. In such process, a considerably large portion of those active reactants and redox products per se have the Janus face with being reduced and also oxidized as ‘redoxidants’ (for example, as shown in **Figure 2A**). It is of crucial importance to notice that redox balance between redox stress and anti-redox response is maintained for health life process in a certain robust homeostatic state, as summarized in the ‘redox code’ with a particular conceptual proclivity for ROS-based oxidative stress and antioxidant protection 29,30,31,32. The inaccurate scenario of ‘central redox theory’ biased for the oxidative stress defense system 11,33,34 should have been established on the foundation of the so-called ‘ox-tox’ hypothesis 35,36, as a commonly accepted antioxidant mechanism to detoxify the bourgeoning production of O_2_ and ROS. This is exemplified by the statement in believing to be "…richly elaborated in the oxygen-dependent life where activation/deactivation cycles involving O_2_ and H_2_O_2_ contribute to spatiotemporal organization for differentiation, development, and adaptation to the environment" and where "disruption of the organizational structure during oxidative stress represents a fundamental mechanism in system failure and disease" 29.

### The evolving redox-based systems

As a matter of fact, the cellular life began in an anoxic ferrous ocean approximately 3.8 billion years ago (Ga) and then experienced its nearly seven-eighths of evolution course under almost no or less (< 0.1% PAL) oxygenized environments 16 17 40. As such being the case, the appearance of those so-called ‘classic antioxidant’ enzymes, which include catalase (Cat), superoxide dismutases (SODs), glutaredoxin (Grx), thioredoxin (Trx), peroredoxin (Prx), sulfiredoxin (Srx), and other oxidoreductases, seems to be rather coincidently and closely aligned with the origin of anoxygenic photosysthesis (i.e., H_2_S with CO_2_ is reduced to yield methane and oxidized polysulfides) over a billion years prior to the great oxidation event (GOE, arising from the incrementing oxygenic photosysthesis (i.e., H_2_O with CO_2_ is oxidized to yield O_2_ and methane) 21,37,41,42,43,44. Such O_2_/ROS-detoxified defense systems 43 45 46, together with the early respiratory nitric oxide (NO) reductases (NORs, structurally related to the present-day respiratory O_2_ reductases (alias cytochrome/quinol oxidases or Complex IV) 23 44, were further corroborated to be existing in the ‘obligate O_2_/ROS-tolerant anaerobes’ created at the early stages of the evolution of life, and even emerged in the primordial populations of protocells known jointly as the last universal common ancestor (LUCA) of all life forms on the Earth. Importantly, several lines of experimental evidence have validated that such ‘classic antioxidant’ enzymes (e.g., Cat, SOD, Trx, Grx, Prx) are essentially involved in the redox metabolism of H_2_S and polysulfides (H_2_S_n_, n=2-8), which are collectively referred to as RSS, viewed as a physiological relevant O_2_-sensing mechanism 37 47 48 49. Altogether, it is inferable that these ‘antioxidant detoxification’ systems should be lent and also evolutionarily extended from copying with the prior sulfur-based redox reactions in anti-redox signaling response to RSS, possibly via RNS, towards dealing with the nowadays prevailing oxygen-based redox reactions in relevant cytoprotective response to ROS (**Figure 2B**). This notion was also proposed by 39 49 50, albeit it remains really hard to distinguish disparate causal efficacy of RSS from ROS in relevant redox stress responses by means of the hitherto established methodologies 51, whereby RSS was, rather, measured more sensitively than ROS per se 37,49,52,53.

Clearly, it was conjectured from the mounting evidence that the onset of life was driven by redox disequilibria resulting from the evolution of nascent terrestrial niches, followed by co-evolution with its surviving environments during a long-term highly reducing (H_2_S/RSS) to another gradually oxygenated (O_2_/ROS) status 22,37,54,55,56. Such biological evolution of life takes place abiding by (pre)Darwinian law 38,57 of the nature selection for its novelty to ensure that its functional information will increase within the possibility space of configurations, which are also preferentially selected based on its functions and enable all of those growing functions to be efficiently performed, during the redox-driving process (**Figure 2C**). Hence, the origin of life and its ensuing evolution appears to be ‘open-ended’, forging its adaptations to the changing environmental challenges and constructing its expectable functional configuration spaces, all of which are selected in a stochastic manner 38 58. Collectively, the redox-driving force (provided by redox recycling reactions and interactions) should play a central role for the evolving life system to be selectively self-organized, self-replicated and self-maintained. In the redox central theory, redox code remains as a quintessence of the conserved functional traits achieved from adaptive co-evolution of life with its environments. Yet, such ‘redox code’ should also be refined by encompassing the H_2_S/RSS-based redox and anti-redox principles, because this ever-existing H_2_S/RSS-based system exerts certain pivotal and indispensable effects to be stored for ‘redox memory’ and then reused for ‘redox recycling’, which are substantially disparate from those ‘one-off’ effects of the O_2_/ROS-based redox system 37,47,53. More importantly, a considerable key portion of the O_2_/ROS-based redox and anti-redox effects within the ‘originally defined redox code’ 29 are de facto transferred to the H_2_S/RSS-based redox system and hence indirectly executed by this reactive thiol-switching system 26,37,50,59.

## REDOX REGULATION OF PHYSIO-PATHOLOGICAL FUNCTIONS IN THE NATURE ECO-EVO-DEVO PROCESS 

As an evolving self-organizing system, life is simply dichotomously defined to comprise two parts (selected for adaptation and variation) as a whole, by a Darwinian evolutionary law 38, along with another law of incrementing functional information, albeit as its complexity is gradually increased 58. The major stably-selected portion of life should be made of those predominantly statically-persistent systems with a stronger robustness. By stark contrast, the remaining variated part of life should be principally composed of those dynamically-persistent systems with a versatile plasticity to explore its novelty. The variating novelty is determined by newly generated organic codes, an arbitrary set of those principally-coding rules from a geological point of view that amounts to a sudden event 60,61,62, which could be yet predicted by its experience-building memory (storage of functional information acquired by sensing and measuring) and/or by a memory-independent fashion 58. Thereby, it is plausible that those stable configurations of life and its selected physiological functions should be maintained and perpetuated by its robust homeostatic mechanisms, and further remodeled by two distinct and even opposing mechanisms, homeorhestasis and allostasis, respectively 63.

### Redox regulation by distinct mechanisms 

In this regulatory process, redox and anti-redox systems certainly play vital determinant roles in orchestrating the configurations of life during its morphogenesis and regulating the architecture of its physiological functions at normal morphostasis 50,64,65,66,67. This is due to the fact that redox and anti-redox systems have inherently evolved to establish a set of *lingua franca* for life 39, unifying the entire eco-evo-devo (i.e., ecological, evolutionary, and developmental biological) process 30,68,69,70. Besides ‘redox code’, there are also existing those innately codifying biology mechanisms, which are executed directly by the redox electrochemical reactions and the relevant specific biochemical modifications. As aforementioned, most of these (but not all) enable to be stored for ‘redox memory’ within the redox-derived *lingua franca* dictionary and, if being later required for biological needs, reused for ‘redox recycling’ to directionally switch redox regulation of physiological and pathophysiological functions. These redox-reactive species and redox-bearing carriers also enable to be transported throughout intracellular and extracellular (interstitial) fluids, insomuch as to allow for efficient redox signaling communications between different subcellular organelles, distinct cell lineages, and diverse tissues and organs 26,37,47,71. 

Such redox communications are yet remaining to be, to certain extents, confined by those inherent ‘kinetic’ barriers that frustrate their immediate diffusion and free dissipation so as to reach a somehow (electrochemical) equilibrium, leading to disparate redox-compartmentalized distributions throughout intracellular and extracellular contexts 72,73. Consequently, discrete ‘redox potential gradients’ with a selective modularity are established in diverse topovectorial processes and ‘phase spaces’ that are redoxokinetically compartmentalized so to give rise to distinct redox potential energy capacitors (**Figure 2D**), as evinced by distinct status of ‘redox phenotypes’ 74. Such ‘redox gradients’ have been shown to act as an internal impetus to the embryogenesis and ensuing physiological development, and healthy growth during life process 30. But, conversely, ‘aberrant redox gradients’ were also implicated in the ageing and pathogenesis of relevant diseases 31,50,72. 

In addition to redox reactions and interactions with all other regulatory elements (e.g., organic codes, topogons, minimotifs) and signaling molecules in redox interactome and systems biology, the redox electrochemical reactive systems also enable to arouse certain extents of their intrinsic quantum biological effects on the relevant physio-pathological regulation by virtue of their unique quantum mechanics’ mechanisms 75,76. Thus, it is inferable that such redox-reactive quanta (along with putative quantum effects exerted possibly via a system of ‘meridians’ recognized for the long history of traditional Chinese medicine 77) could play an essential role in critically unifying all systems networks to regulate diverse physiological and pathophysiological functions across all distinct strata of life from molecular and subcellular levels to the whole body.

### ‘Redox code’ acquired and refined from the eco-evo-devo process

The self-organizing life appears to be also conjectured as a self-manufacturing artifact by self-copying and self-codifying according to those pre-existing templates and/or coding rules, all of which are selected by (pre)Darwinian evolutionary law, during its nature eco-evo-devo process 38,62,78. All distinct types of constituents of life (e.g., components, adaptors, topogons, minimotifs, templates, and codes) are stereochemically patterned and further self-assembled in certain topovectorial phase spaces to yield diverse topoforms (each with specific physiological functions and unique behaviors) of molecular machines, subcellular apparatus, body-planned cells, tissues, organs or organismic individuals (**Figure 2D**). All of these have well fulfilled according to their cognately-coding rules in a combination with their topological orders, biochemical relationships and ontological status 61,79,80. In reality, most components and adaptors of the living systems should be plastically changed in the long run of the eco-evo-devo processes, but only a few of them (also including codes, templates, minimotifs, topogons) must be absolutely conversed to gain the robustness of living systems. Of note, the arbitrary coding rules are also highly conserved, because they serve as a universally governing mechanism that nature has constantly employed in the course of life’s origin and ensuring evolution. From the eco-evo-devo scenario, it is inferable that the ‘redox-based coding rules’ should predate the establishment of the commonly accepted genetic code and other organic codes, because ‘redox code’ (defined by Jones & Sies 29) has originally taken an early leading place, particularly in transforming the inanimate to animate worlds 37,81,82. This notion is based on the finding that far ancient genetic templates are likely executed by the living fossil-like ‘basic-region zipper minimotifs’, albeit with a low infidelity 83,84,85, which are later evolutionarily replaced by the polynucleotide templates (i.e., RNAs or DNAs).

**Figure 3:**
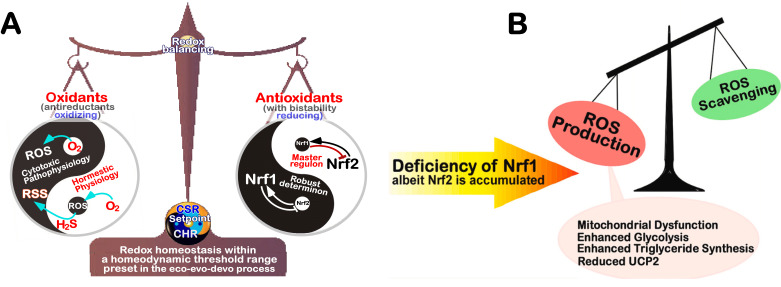
FIGURE 3: Distinct roles of Nrf1 and Nrf2 in redox homeostatic equilibrium. **(A) **Maintenance of redox homeostasis by balancing between oxidants (and/or anti-reductants) and antioxidants (also with reducing bistability) within certainly presetting homeodynamic threshold ranges in the eco-evo-devo process. In each side, there is also existing a redox ‘Yin-Yang’ equilibrium that abides by a dialectic law of the unity of opposites. Of note, distinct roles of Nrf1 (as a robust determinon) and Nrf2 (as a master regulon), which are dubbed as two entangled ‘Yin-Yang’ quanta, are integrated for their coordinate control of redox homeostasis. CHR and CSR represent cell homeostatic and stress responses, respectively. **(B)** Deficiency of Nrf1 leads to severe imbalanced redox stress arising from augmented ROS production, but this phenotype cannot be rescued by the hyperactive Nrf2, albeit it was accumulated in *Nrf1^(((^* cells (as graphically adapted from 115).

According to the general ‘code theory’ modelled by Barbieri 62,78, redox code is referred to as a set of the consensus rules for codifying all redox-based spatiotemporal systems and their interactions with other biological systems in the body-planning networks of life, and tightly governing its intrinsic anti-redox responsive mechanisms to cell stress. Such ‘refined redox code’, like those organic codes 62, is also reckoned to have gone through five possible phases from its origin to being completely established. In the beginning of redox code, its 1^st^ version had emerged possibly as a means of performing a series of particular redox-relevant functions by diverse redox reactive cascades with organic macromolecules, but with certain extents of necessary ambiguity to enable a closer linkage of the inanimate to animate worlds during the origin of life. Such ambiguity was, in the 2^nd^ phase of redox code, steadily reduced by gradually-improved compartmentalization of redox-based systems to yield distinct gradients and stereotyped capacitors with different redox potential energy, enabling life to effectively distinguish its internal milieu of life’s body from the surrounding environments, but enable certain interchanges between the internal and external environments across its membrane-based platform system. In the 3^rd^ phase, the redox code was optimized by selecting its specificity to monitor those particular physiological functions by gradient redox potential energies within distinct redox compartments, and across those redox-configured microdomains, e.g., by NAD(P)H/NAD(P)^+^-driven redox metabolisms, and O_2_/H_2_O_2_-, NO/RNS- or H_2_S/RSS-leading redox signaling networks, altogether with relevant (bio)chemical modifications. In the 4^th^ phase of redox code, its major transition is much likely yielded by redox switching of putative physio-pathological functional activation or inactivation of some molecular machines and subcellular apparatuses primarily by thiol-active and/or -reactive redox (re)cycling networks, especially upon the cellular redox sensing to signaling responses with interactomes. The stress-leading damage repair and disposal mechanisms are also likely embedded in this major transformation phase, which is manifested by certain changed configurations. In the last 5^th^ phase, the conservation of redox code is de facto embodied by either programming or reprogramming of redox signaling to cellular redox metabolism and relevant gene expression profiling critically for its genetic, and epigenetic (and topogenetic) responses to diverse redox stress. The inheritable conservativity of redox code may be enforced by intra- or inter-generationally incorporating selenocysteine (Sec), persulfidated cysteine (CysS_n_H, n >= 2) and other redox-active not-yet-identified amino acids (e.g., HO-Pro) into redox proteomes during alternative translation of potential redox-responsive gene transcripts 71,86. Of note, those known basic-region (zipper) superfamily of transcription factors, HIF1α, Nrf2 (encoded by *Nfe2l2*) and Nrf1 (encoded by *Nfe2l1*, **Figure 3A**), together with NF-κB and FOXO, are identified to act as the conserved players in the redox programming or reprogramming responses 64,65,87,88, and further govern the proteostasis of the redox proteome during coping with stress 89.

### Redox kinetic barriers dictated by membrane topogenesis (via ‘topogenetic code’) 

Differential positioning of redox gradient distributions in distinct topovectorial phase spaces and subsequent repartitioning of redox-based systems across diverse topovectorial spaces are all dictated predominantly by redox-compartmentalized membranes serving as certain ‘kinetic’ barriers (including redoxsome). More importantly, such membranes have fulfilled an irreplaceable pivotal role in transmitting architectural orders during self-organization of cellular life 90,91,92. This is just because cell membranes possess an indispensable topological property to be never constructed *de novo*, enabling them to be always growing from its pre-existing membranes and passing down from one generation to the next in an uninterrupted chain of descent 93 94. Since this remarkable innate property of cell membranes has been defined as a chromosome-like ‘membrane heredity’ 61 93, it is inferable to be determined by its ever-existing, but not-yet-identified, ‘topogenetic code’ for manufacturing distinct types of membrane topogenesis and further orchestrating most of the self-assembling topobiological structures of cellular life in real tempospatial phases on diverse membrane-based platforms, to perform accurate physiological functions in orderly organization. Such place (i.e., position in space)-dependent morphogenesis endowed with unique physiological functions during the eco-evo-devo process is dictated by membrane-associated topology and topobiology 92 95 96.

Initially, topobiology was only referred to as the place-dependent interactions of differential cell adhesion to extracellular matrix (ECM) via membrane-based platforms to drive morphogenesis in the developing embryo, and the origin of living systems 96,97,98. This paradigm was later revised to include force-dependent molecular switches, cell and tissue tension and reciprocal interactions with the microenvironments through conversed decision-making modules (switch, connector, capacitor, transistor and topogon) to specify the morphogenesis of multiscale forms with a unique functionality 95. The morphostasis is maintained by spatial segregation and organization of those anchored proteins and secreted factors through emergent properties of tissues, including tension fields and energy optimization. The concept of topobiology, as an important key decision-making mechanism accounting for the eco-evo-devo process has been further refined by applying its original mathematic and physic laws of topology into life science and medicine, aiming to unlock real Gordian knots (e.g., of redox electrochemical and quantum systems) in biology 99,100,101,102. Collectively, such inheritable membranes cannot only determine the compartmentalization of redox microdomains and its kinetic systems, and also dictate the orderly morphogenesis of self-organizing life and its morphostasis (of a given topoform with specified physiological function).

On the evolving membrane-based platforms, the redox-reactive system of H_2_S/RSS together with iron sulfide (FeS) plays a vital role in the morphogenesis and morphostasis of early life 37 103. This is supported by molecular evolution of ancient ferredoxin (Frx), which consists of only 23-aa with four Cys residues enabling a [4Fe-4S] cluster to be anchored on a positively charged mineral surface and thus mediating the electron transfer in the primordial membrane system prior to the origin of species diverged from the LUCA 104. Subsequent evolutionary respiratory NO to O_2_ reductases, possibly with Frx, contribute to energy metabolism by distinct species’ membrane systems 23,105, but also to the yield of H_2_O_2_/ROS, NO/RNS and H_2_S/RSS in the electron transferring processes 47,65. The membrane-tethered NAD(P)H oxidases (NOX, also called NAD(P)H-dependent reductases) cannot only reduce O_2_ to yield most of ROS (i.e., O_2_^-^ and H_2_O_2_) and remove O_2_ to water (H_2_O) 106. Furthermore, O_2_/Fe^2+^-dependent prolyl hydroxylases (PHDs, as transmembrane enzymes governing the HIF1 turnover, but conversely induced by HIF1 107 108 109 110), together with Cu^2+^-dependent lysyl oxidases (LOXs), of collagens (secreted from the ER to the ECM) dictate the rigid formation of its stereotyped ‘tensor networks’ by multiple-crosslinking with cell adhesive molecules, transmembrane-tethering proteins (e.g., integrins and fibronectins), and consecutively associating with membrane-enclosed cytoskeleton and karyoskeleton, together with all the subcellular organelles being connected with one another through their membrane extensions (e.g., stromules, peroxules, and matrixules). Collectively, these membrane-based topobiology (possibly via ‘topogenetic code’ rules for body plan) cannot only enhance the compartmentalization of redox microdomains and its kinetic systems in all distinct intracellular and extracellular matrix contexts, but further determine distinct topoforms of cell lineages (by their interaction forces), tissues, organs and even individual beings in distinct positioning sizes of topovectorial phase spaces.

### Coordinated control of redox responsive mechanisms by Nrf1 and Nrf2

Overall, such diverse topogenetic phenotypes are also modulated by distinct redox status, as dubbed ‘redox phenotypes’, which is being maintained at certain physiological homeostasis during normal conditions. Once such homeostasis (along with morphostasis) is markedly disrupted by excessive redox stimulation for a long term, this results in relevant pathophysiological deterioration and pathogenic phenotypes of many chronic diseases including cancer, diabetes, atherosclerosis, and neurodegenerative diseases 111 112. Hence, in order to combat excessive redox stimulation, all cellular life forms have been evolutionarily armed with a series of innate powerful anti-redox defense systems. Amongst them is a set of such essential anti-redox, detoxification and cytoprotective mechanisms governed by the cap 'n' Collar and basic region leucine zipper (CNC-bZIP) family of transcription factors 87,113,114. Of striking note, Nrf1 and Nrf2 are two principal CNC-bZIP factors in vertebrates, to finely tune transcriptional expression of cognate genes by binding the consensus antioxidant or electrophile response elements (AREs/EpREs) in their promotor regions. In fact, we unraveled that the allelopathic potentials of Nrf1 and Nrf2 (both resembling two entangled ‘Yin-Yang’ quanta that comply with a dialectic law of the unity of opposites, as illustrated in **Figure 3A**) are exerted so to coordinately govern a certain redox physiological homeostasis to be maintained within the presetting threshold ranges 115. 

To date, a large number of studies on Nrf2 have revealed that it functions as a master regulator of antioxidant response and relevant redox signaling 116. However, such versatile Nrf2 acts de facto as a promiscuous, but not essential, player for optimally ARE-binding to most of its target genes 117. This supports a concluding notion that Nrf2 is dispensable for normal growth and development 118, with not any obvious pathological phenotypes being manifested in its global knockout mice. As a matter of fact, Nrf1, rather than Nrf2, is a living fossil with its ancestral properties, because it shares an evolutionary conservativity with SKN-1, Cnc and Nach factors 114. Like its ancient homologues 119 120, Nrf1 is topologically integrated within the ER, then repartitioned and dislocated across ER membranes to enter extra-ER subcellular compartments, in which it is processed to yield a mature N-terminally-truncated factor, similar to Nrf2, before transactivating its cognate target genes 121,122,123. Collectively, such a highly-conserved, indispensable role is fulfilled by Nrf1 (with a unique topobiological feature), but not by Nrf2, for maintaining the steady-state threshold of normal redox homeostasis. This is much likely to further dictate the morphostasis of distinct topogenetic phenotypes with healthy physiological functions, because the loss of its function results in severe endogenous oxidative stress, as accompanied by disruption of cellular lipid, glucose and protein homeostasis and organ integrity, leading to cancer development and malignance, even though Nrf2 is aberrantly accumulated in such *Nrf1α*-deficient cells (**Figure 3B**) 115.

##  SCIENTIFIC CONCEPTUAL EVOLUTION OF REDOX STRESS FROM PHYSICS AND CHEMISTRY TO BIOMEDICINE 

**Figure 4:**
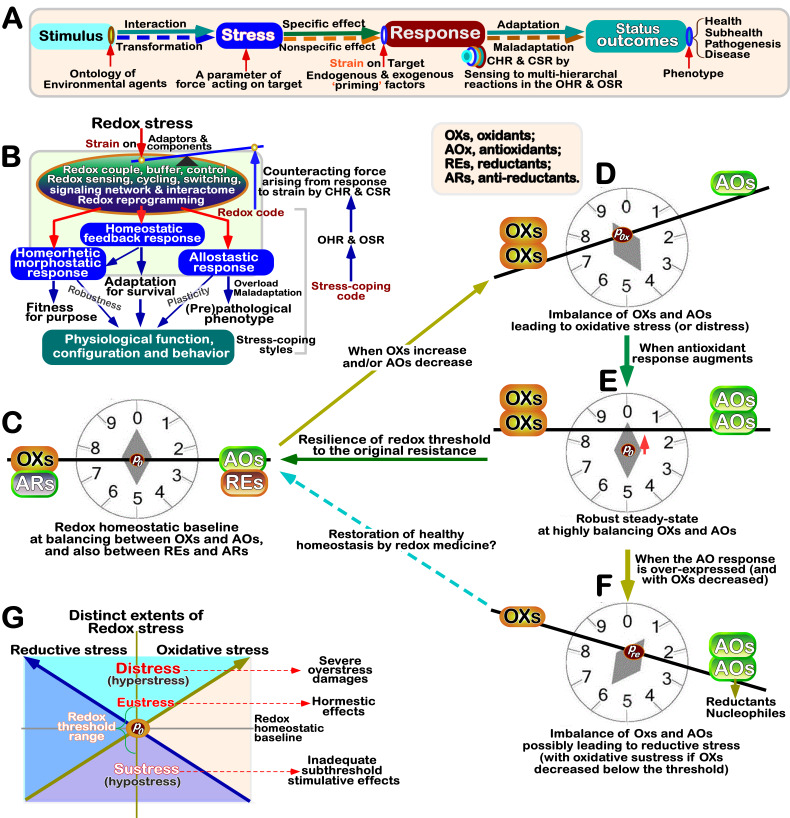
FIGURE 4: Axiomatic representation of redox stress and defense response systems in cellular life. **(A)** Axiomatic representation of cellular adaptive response to stress induced by distinct stimuli. The stress and its resulting strain are viewed in physics as two coherent parameters of force acting on targets, whereas its counteracting force arises from adaptive responses to strain by cell homeostatic and stress responses (i.e., CHR and CSR). Taken together, different outcomes are output as an integrated adaptive or maladaptive status with distinct physio-pathological phenotypes. **(B)** Schematic diagram of redox stress and its coping responses by distinct mechanisms and obligate coding rules (i.e., stress code and stress-coping code). The detailed explanations are provided in the text. OHR and OSR represent the organismal homeostatic and stress responses, similar to CHR and CSR at cellular levels, respectively. **(C-F)** Schematic rationales for redox balanced or imbalanced statuses, along with the concise explanations as indicated. **(G)** A graphic abstract of distinct extents of redox stress, including oxidative stress and reductive stress. Distress (also called hyperstress) can lead to severe overstress damage, whilst sustress (also called hypostress) could give rise to inadequate subthreshold stimulative effects, in addition to eustress enabling adaptive hormestic effects for the healthy survival.

To gain a better understanding of physio-pathological effects of redox stress (also including the secondary redox stress trigged by many other types of stress) on biological systems and their correspondingly coping strategies, it is rather imperative to properly define the ‘stress’ and the resulting ‘strain’ that triggers all relevant compensatory, adaptive, maladaptive and neutral responses in target cells, tissues, organs and organisms (**Figure 4A**), all of which are placed at stressful bottlenecks of the nature evolutionary selection pressures 124. This is because distinct stress-induced changes and its target coping responses give rise to certain heritable phenotypes for fitness, and also leads to some phenotypic variations at the population levels 88,125, which is conductive to adapt for host survival and maintain its homeostasis under stress arising from the changing eco-evo-devo processes.

### Axiomatic concepts of stress and relevant parameters revisited by principles of physics

#### Physics-based concepts of stress and parameters in biology 

Although the contemporary concepts regarding ‘stress’ employed conventionally in a wide variety of scientific disciplines have evolved over the past 2½ millennia 126 127, it remains to be a hot subject of distinct scientific debates. In fact, the word ‘stress’ originates etymologically from the common proto-Indo-European root ‘*str*’, which has been historically associated with exertion of pressure 126. Until 1660, ‘stress’ was introduced in mechanical physics and accurately formulated by Hooke’s law of elasticity (*F*=Δ*L*×*k*); the relationship of ‘stress’ (σ=*F/A*=Δ*L*×*k/A*, a ratio of the internal force brought into play when a substance is distorted to the area over the force acts, whereas the external force producing the distortion was defined as ‘load’ 128) over the resulting ‘strain’ (ε=Δ*L/L_0_*, a ratio of such distorted change in size or shape to their original ones) was further scientifically defined by Thomas Young’s elastic modulus (*E_m_*= σ/ε = *L_0_*×*k/A*) in 1807 129. Such original meaning of stress per se was also used to denote the internal force generated within a living body by any force which leads to strain (or distortion) of the body 128. The Hooke’s law was recently extended by Dietmar Kültz for its application in biological systems, aiming to properly and unambiguously refine the concept of stress by its original physic principles, which was hence restated as *F*=Δ*H_c_*×*k* 124. Here, *k* is a constant that describes the phenotype of a biology system at the time of exposure to the force *F* arising from stress, while Δ*H_c_* represents the extent of dysregulation of this system differing from its set-point of the homeostatic norm (*H*) of the most critical and limiting physiological variables *c*.

As *F*= ∫*f*(Δ*H_c_*)×*k* was refined here, this is because a maximally informative yet minimally complex set of variables Δ*H_c_* may be modeled by a certain function (*f*) of their core endogenous network 130,131 and further integrated (∫) in ontogenetics and phylogenetics 132, the topology of which would accurately reflect the amount of strain (deformed) on this system during stress. Since ‘stress σ’ and ‘strain ε’ were restated as σ=* F/R* =∫*f*(Δ*H_c_*)×* k/R* and ε = Δ*H_c_/H_c,_* respectively 124, this system’s elasticity (or plasticity) may be formulated as *P*=[∫*f*(Δ*H_c_*)]/Δ*H_c_*×* H_c_*×* k/R*, and further simplified as *P* ≈ *H_c_*×* k/R*, only if *f*(Δ*H_c_*) = Δ*H_c,_* in which R represents the robustness of a living system (i.e., cell or organism) and acts as an inherent property of this evolving, complex dynamic system. Moreover, if the stress potential energy stated *E_s_* = ½ *k*× (Δ*H_c_*)^2^, it may be rewritten as *E_s_*= ½ *k*× (ΔG+ΔS×*T*)^2^, because free energy (ΔG) is formulated by ΔG= Δ*H_c_*-ΔS×*T*. Herein, the stress-reactive entropy is represented by ΔS, it is due to the nature of stress on the atomic level in dense polymer systems (of cell or organism) was classically viewed as being molecular, based on the ‘entropic spring’ concept 133, stating the intrinsic monomer stress by contribution of the individual monomer to the macroscopic stress referred to a local moving coordinate system in which its backbone bonds attached to the monomer are fixed. ΔG is equal to do the responsive work, i.e., *W*= ∑ (*W_CSR_*+*W_CHR_*+*W_OHR_*), arousing a counteracting impetus of the host against stress by distinct coping mechanisms 88 125 126. Collectively, stress, strain, plasticity and robustness, along with other relevant parameters, can all be subjected to precision evaluation and axiomatic demarcation by princples of physics.

#### Non-physics-based concepts of stress response evolving in biomedicine

However, it should also be noted that all those other evolving definitions of stress, as known so far, seem to be largely irrelevant to the original meaning of its physics 128 134 135 136 137 138. As such being the case, multi-faceted stress and relevant responses remain to be partially delineated by their evolving concepts nearly for a century after being introduced into biology, revealing the reaction and interaction of diverse changing environmental (including social and psychosomatic) stimuli with all life systems from distinct aspects of bioscience and medicine (**Figure 4A** and **B**). From a fluid matrix of life’s body demarcated as ‘millieu interieur’ by Claude Bernard 139 124 to its steady state named as ‘homeostasis’ by Walter Cannon 141 142, the stress and strain of homeostasis were first described in a biological system, ‘in terms of a homeostatic index (*H_0_/H_c_*), which states the measured ability to react, without disturbance of the fluid matrix, to a group of standard stresses that may readily distturb it’ or cause ‘an excessive strain upon the protective agencies’ 142. Later, the stress may be defined by Hans Selye as a ‘nonspecific deviation from the normal resting state; it is caused by function or damage’, but also ‘stimulates repair’ 143. This is just due to ‘the facts that stress is not necessarily the result of damage but can be caused by physiological function, and that it is not merely the result of a nonspecific action but also comprises the defense against it’. Thereby, the remaining portion, after subtraction of all the specific changes caused by distinct stimuli and their reacting targets, is considered as ‘the general-adaptation syndrome (GAS) produced by diverse nocuous agents’ 144. The GAS was determined by such nonspecific commonly-shared responses to all types of stresses, with all the relevant activities (e.g., to affect behavior, temperament, physiological homeostasis and anatomic morphostasis) being harmoniously integrated by the hormonal and nervous system of organisms. The autonomic neuroendocrine responsive systems (e.g., HPA and SAM predominantly) and extended immune systems to any stress were all collectively referred to as ‘stress response system’ 126 127 143. By contrast with physiological protective responses for the purpose of a fit healthy resilience, prolonged or exaggerated stress is rather accompanied with a continuum of inappropriate adaptive to maladaptive responses, ultimately leading to development of a series of pathological phenotypes and even chronic diseases (i.e., ‘stress-led syndromes’, including psychosomatic disorders) 126,143,145,146.

### Ontological concepts of redox stress revisited by principles of chemistry

#### Scientific basis for redox stress in chemistry

Redox stress is integrated in term of a Janus-faced parameter of redox electrochemical potential disequilibrium arising from ‘biologically inappropriate’ reactive imbalances amongst all oxidative, antioxidant, reductive and anti-reductant agents involved during interaction of adequate stimulus with the host and ensuing transformation to its adaptive responses. This collective definition of whether is prone to favor oxidative or reductive stress depends on the principle of which stress changes the nature of redox potential energy. This may be accurately determined by the Nernst-Peters derived equation *E_h_* = *E_0_* + 0.03× log_10_ [oxidant/reductant] 147. Hence, Δ*E_h_* = *E_h_* -*E_0_* was calculated by a logarithmic ratio of [oxidant] to [reductant] (Δ*E_h_* = 0.03× log_10_ [oxidant/reductant]; their relationship was also shown in a sigmoid buffering curve 147. The term ‘near equilibrium’ is defined to describe the reactions in which there is essentially zero free energy (Δ*G* = 0) available from putative electrons (and/or hydrogens) being transferred or exchanged because this reaction is maintained so very close to an equilibrium of redox distribution between reactants and products 32. That is, Δ*G* = Δ*E_h_* = 0, such that [oxidant] is equally balanced to [reductant]. Therefore, it is inferable that such a redox equilibrium of biology systems in cells or organisms should be preserved only by tightly governing a ratio of ∑[oxidants] to ∑[reductants] balanced to near zero extents (as theoretically shown in **Figure 4C**).

Such being the case of an imbalanced redox status, whether or not an onset of the oxidative or reductive stress remains depends on the antioxidant or anti-reductant capacity of life, respectively, together with its detoxifying cytoprotective capability in its emergency response system. This poses a challenging question of how the cells and organism deal with a mounting pool of redox equivalents to properly balance the generation of ‘reactive X species (including ROS, RNS and RSS) and their elimination, and optimize all those redox buffer couples (e.g., NADH/NAD^+^, NADPH/NADP^+^, GSH/GSSG, CysS/CysSX, O_2_/H_2_O_2_, and H_2_S/RSS_n_), particularly during cellular stress. To address this question, it is required to clearly figure out the scientific distinctions amongst oxidative (oxidant) stress, reductive (reductant) stress and redox stress, just because of their previously confounded terms of which had been employed disparately by biologists, but distinguishably from chemists 148.

#### Debating concepts of oxidative stress in biology

The term ‘oxidative stress’ was initially used in 1970 by Paniker *et al*. for a study of the erythrocytic glutathione metabolism affected by glutathione reductase (GSR) deficiency, which was determined by measuring a ratio of GSH to GSSG in the cell response to exogenous H_2_O_2_ (viewed as a stimulus of oxidative stress imposed on the cell) 9. At the end of 1970, the intracellular H_2_O_2_ changes were first discovered in eukaryotes by Helmut Sies and Britton Chance through a pioneering collaboration by spectrophotometry of isolated hemoglobin-free perfused rat livers 149. Later, such a phenomenon of ‘oxidative stress’ was originally formulated by Sies H. in 1985 as ‘a disturbance in the prooxidant-antioxidant balance in favor of the former, leading to potential damage to intact cells and organs’ 150 151. Since that time to date, oxidative stress has been inspiring too many investigations of all relevant fields as a mechanism for inducing a unique set of adaptive homeostatic responses in cells and organisms (i.e., CHR or OHR, in **Figure 4B**). This notion has also evolved in biomedical research as an experimental method for exploring adaptive redox responses and as a putative determinant of ageing and other pathological processes 148. In this process, ‘oxidative stress’ was also further refined by Jones D.P. in terms of ‘an imbalance between oxidants and antioxidants in favor of the former oxidants, leading to a disruption of redox signaling and control and/or molecular damage’ 10,12. 

Quite recently, the concept of oxidative stress was updated by Lushchak VI and Storey KB, who revisited it in terms of ‘a transient or long-term increase of steady-state ROS levels, disturbing cellular metabolic and signaling pathways, particularly ROS-based ones, and leading to oxidative modifications of an organism’s macromolecules that, if not counterbalanced, may culminate in cell death via necrosis or apoptosis’ 152. Overall, as to biologists, oxidative stress implies that a cell or organism produces or is exposed to an excess of the highly reactive molecules (i.e., RXS), predominantly oxygen- and/or nitrogen-centered ROS/RNS, which exceeds its endogenous antioxidant capacity (to be endowed on the organism with a host of all those oxidoreductases, reductases, and small-molecule antioxidants, as illustrated in **Figure 4D**).

#### Major challenges for precision definition of oxidative or reductive stress by chemistry

Herein, it should be noted that a critical pitfall of oxidative stress may lead to indiscriminate use of this term as a global concept with certain ambiguity and circularity, but without a clear relation to redox chemistry, in each of particular cases 10. Accordingly, by reevaluating those previously-debating concepts of oxidative stress from the chemical perspective, it was found that most of those definitions by biologists appear ‘too vague and imprecise to recognize explicitly what the oxidants are set in a given set of intracellular and extracellular circumstances and how their reactivity is countered, controlled or quenched, and most importantly, what the reductants are really to which the oxidants must be coupled for the redox reactions to proceed’ 148. Rather, to achieve a precision definition of oxidative stress by redox chemical ontology in complex biological systems, there are existing certain unavoidable challenges for the following reasons. i) It is very hard to identify or quantify each specific species of ROS/RNS (not as a global index 153), in a hitherto uncompleted picture of all their sources, sinks and fluxes 148. ii) Their chemical reactions, particularly with the very short-lived free radicals involved in a wide range of redox potential propagating to terminating reaction cascades, are often too rapid to be accurately determined within their availably measured time scales of the presently established techniques 51. iii) The redox compartmental heterogeneity of ROS/RNS in their gradient distributions varies with changing status of the intracellular and extracellular contexts, so that they are difficult to trace in the real-time topovectorial space by relevant reporters, an ideal molecule that can react sufficiently and rapidly with each interest species of ROS/RNS with exquisite specificity, at its low enough concentrations so as not to affect the steady-state level of the indicated species 148. iv) Many of the currently ROS/RNS-estimated methods may also have actually and more sensitively detected RSS, such that they are rarely distinguished from each other 37 49 52. In fact, the ratio of ROS to RSS in cells was examined to be considerably less than three (ROS/RSS < 3), because H_2_S/RSS can be co-produced with H_2_O_2_/ROS (e.g., by SOD, Cat, respiratory chain), so that its production may well exceed the yield of ROS on most occasions 3 47. As such being the case, the term ‘oxidation’ exemplified this ingrained bias as there is no equivalent term ‘reduction’. But, as a biological consequence of oxidative stress, it is cognized only in light of the reductants that have been oxidized in this process and the effects of those coupled redox reactions on the biological functional pathways in which the redox-active molecules are involved. Collectively, it is inferable that this complicated redox biochemical process cannot be well generalized by an overly simplistic biological principle and nor determined by another chemical precision algorithm applied for a statistically complex ensemble of such redox reactions.

Intriguingly, the most of important biological oxidants (connoting oxidative stress) are derivatives from O_2_ (with a proclivity for electrons), but they exist in partially reduced forms, including superoxide anion (O_2_^·-^) and H_2_O_2_, from a simple chemical perspective. Coincidently, these ‘Janus-faced redoxidants’ can oxidize some molecules of the appropriately matched negative redox potential (Δ*E_h_* < 0), as accompanied by reducing other molecules of more positive redox potential (Δ*E_h_* > 0) 148. For instance, O_2_^·-^ can reduce disulfides (to yield reduced thiol forms) and simultaneously oxidize α-tocopherol, leading per se to its oxidation to O_2_ and its reduction to H_2_O_2_, respectively. Similarly, H_2_O_2_ can reduce ferrylhemoglobin (to yield O_2_-carrying Fe^2+^Hb) and oxidize methionine or the thiol-active proteins, leading per se to its oxidation to O_2_^·-^ and its reduction to H_2_O, respectively. As a consequence, certain disulfide linkages are constructed by oxidation of thiol-active proteins to facilitate their proper folding to gain a functional configuration or crosslinking with other proteins in the more oxidizing ER and extracellular environments. From these points, a certain excess of O_2_^·-^ and H_2_O_2_ is not necessary for leading to a simply ‘oxidative stress’. Conversely, this should be just as well viewed as imposing a ‘reductive stress’ potential on biological systems from a chemical perspective, since as partially reduced forms of ROS involving redox reactions in which they reduce those molecules of more positive redox potential (Δ*E_h_* > 0). Therefore, ‘misleading definitions’ of oxidative stress by biologists appear to be only focused on the oxygen-centered nature of ROS without regard to the precision chemistry of redox-coupled reactions in which they can be involved and/or to the precious changes in redox compartments where they are generated or controlled 148. That is, on the contrary, a properly scientific definition of ‘oxidative or reductive’ stress by a chemical principle should be dictated by the flow of those electrons (or hydride anions) exchanged in the redox-coupled reactions in which all the reactive species have been implicated.

#### Proper definition of redox stress as ‘a generic term’ by biological chemistry

Reductive stress was originally observed in hypoxia hepatocytes by Gores *et al*. 13; this phenomenon caused by respiratory inhibition favors the formation of toxic oxygen species (i.e., hydroperoxide), as was accelerated during aerobic but not anaerobic chemical hypoxia. This stress was further found to contribute to lethal cell injury, which was greater during aerobic, as compared with anaerobic, chemical hypoxia, but delayed by desferrioxamine or cyanidanol, rather than by SOD ± Cat, during intermittent or incomplete oxygen deprivation 13. In this setting, reductive stress is only recognized, from a biological perspective, ‘as a complement of oxidative stress in virtue of providing an excess of reducing equivalents’ that cannot be adequately quenched by endogenous oxidoreductases and accommodated by the existing oxygen counterparts in the local environments (due to oxygen being a terminal electron acceptor in the respiratory chain electron transport and redox reactions in living systems) 148. However, such a definition of ‘reductive stress’, if interrogated from a chemical perspective, can also pose the same misleading and confusing readers in this process as the aforementioned ‘oxidative stress’. For an instance of even the absence of re-oxygenation by reperfusion, sustained hypoxia leads to an increase in ROS, albeit as oxidative stress from the biological angle, whilst these species are de facto partially-reduced forms of O_2_ that can evolve in this setting to hierarchically increase reducing equivalents, with their consequences depending on the redox-coupled reactions in which they are involved. Taken together, given the ‘oxidative and reductive’ complexity as yet oversimplified previously with chemical imprecision, ‘redox stress’ should be commonly accepted as ‘a generic term’, to describe the ‘disturbances in the oxidation-reduction reactions arising from an excess of oxidants or reductants, with their functional consequences in a biological system’ 148. Also, it is critically important for ‘a need to be as explicit as possible about the particular molecular species (most of which are Janus-faced and varied within distinct ambient contexts) involved in the redox reactions that modify biological phenotypes’.

### Stress-coping code acquired by evolving cellular life responses

Generally, a physiological or pathophysiological functional consequence of redox stress depends on the extent and duration of whether or not there is a mismatch between the excess of oxidants or reductants in the redox biological systems of organisms and its intrinsic anti-redox capacity to counteract or mitigate their potential damaging effects in the (mal)adaptive responses to those reactive species during the life process (**Figure 4B-F**). Thereby, such redox stress has been recognized, by the eco-evo-devo perspective, as a powerful mechanistic mediator of the life history trade-offs between its traits (arise and reflect constraints imposed by the environment and physicochemical laws during the evolutionary process) 154 155 156 157 158. If an organism can appropriately recognize and respond to the changing environmental stress challenges, with minimal costs, it is an important physiological attribute, with a great adaptive value (**Figure 4C** vs **E**). On the other way round, if this organism is unable appropriately to do so, with greater costs, it fits into a pathophysiological or (pre)pathological attribute, with another great maladaptive value (**Figure 4D** and **F**). Such striking distinctions in those costs and benefits arising from diverse mechanisms that selectively enable for the organisms to cope with the changing stresses experienced from the predictable and controllable to unpredictable and uncontrollable status are manifested to distinctive extents at different life stages, and hence can serve as a strong selective pressure to drive the evolution of life histories 159.

In the life process, a ‘stress-coping code’ is acquired by nature selection for a set of arbitrary rules accounting for discrete organisms to cope with distinct types of stresses (including primary and secondary redox stress, and even psychogenic stress) instigated by all challenges from internal and external environments. Such a proper code for life to cope with stress by ‘the nonspecific response of its body to any demand’, as early conjectured by Hans Selye 160, should be constructed ‘primarily based on the laws of nature, all of which must be accepted’ within the ready-made machine. That is based on how the body works (and/or how it should work) within a stress-coping code founded on nature laws (which was figured out as a basic necessity of living systems, especially work whose fruits can be accumulated), as with the ‘genetic code’ containing the receipt of all those traits inherited in chemical language 160. However, such putative stress-coping code does not yet appear to be unveiled heretofore, albeit animal adaptive stress responses fall into two coding styles (i.e., proactive hawk and reactive/passive dove) of their behavior, physiological health and relevant pathological diseases 158 161 162 163. 

Following nature principles applied to code biology 61 62 79, ‘stress-coping code’ could be defined as a series of set rules that can enable distinct cellular life forms to cope with all various stresses caused by changing internal and external environments. Such ‘stress-coping codes’ have experienced five phases, as well done by other organic codes 62. In the beginning phase, the first version of the coding rules appears in a living system as means of performing a particular function to cope with stress by governing all relevant adaptors and components, which were orchestrated into an ensemble of both ‘stress-sensing’ (e.g., physiochemical, electrochemical, or quantum chemical) reactive cascades and its ‘strain-leading’ biochemical metabolisms with its responsive signaling pathways. The certain ambiguity of this code in its second phase is evolutionarily reduced insomuch as to steadily improve its particular functional specificity to cope with stress, as a result, giving rise to distinct subsets of multi-hierarchical stress-coping physiochemical reaction cascades in their real spatiotemporal orders coupled with multi-dimensional signaling responsive transduction pathways, comprising a complex gradient-ordered ‘tensor’ network. Such code in its third phase is further optimized by distinct mechanisms (such as hormesis, homeorhesis, homeostasis, alloststasis and morphostasis, as in **Figures 4B** and **G**). The consequence enables a living life to establish two major classes of ‘stress-coping responses’, i.e., cellular homeostatic response (CHR) and cellular stress response (CSR), both of which are also extended to organismal homeostatic response (OHR) and organismal stress response (OSR), as described by Dietmar Kültz 124 125. In the fourth phase, this optimized code is further subjected to a major transition (by an integral functional and structural means of distinct strata of molecular, subcellular, cellular, tissue to organismal and even population levels), so far as to construct a perfect responsible set of ‘stress-coping systems’ coordinately regulated by distinct mechanism (as proposed by two groups of Kültz D. 88 124 164 and Chrousos G.P. 126 127 165). In the last fifth phase, such stress-coping code continues to evolve by selective programming and further reprogramming with its requisite specificity. So ultimately, it becomes an intact set of highly conversed inheritable rules, that can tightly monitor all the relevant responsive behavior, physiological and pathophysiological adaption or even maladaption to various stresses (e.g., two distinct coping styles), aside from that can be virtually transmitted by genetics, epigenetics and topogentics to the descendants. For example, the CSR is one of the most highly evolutionary conserved responses inherent in all cells, which represents a prerequisite for evolution of the neuroendocrine stress response (NESR). This NESR cannot only integrate the response to stress in metazoans, but also, in turn, is another prerequisite for the evolution of psychological and/or emotional stress response (PESR) in the vertebrates to its preeminence in humans 88,124. Overall, stress is not something to be avoided, no matter what happens; there thus arises a demand for stress-coping code to provide the necessary principles for the tempospatial order to perform the tasks required to maintain the homeostasis of cellular life, and also resist and adapt to the changing influences from its internal and external environments (including redox stress).

##  QUANTITATIVE STRATIFICATION OF REDOX STRESS AND ANTI-REDOX RESPONSES 

**Figure 5:**
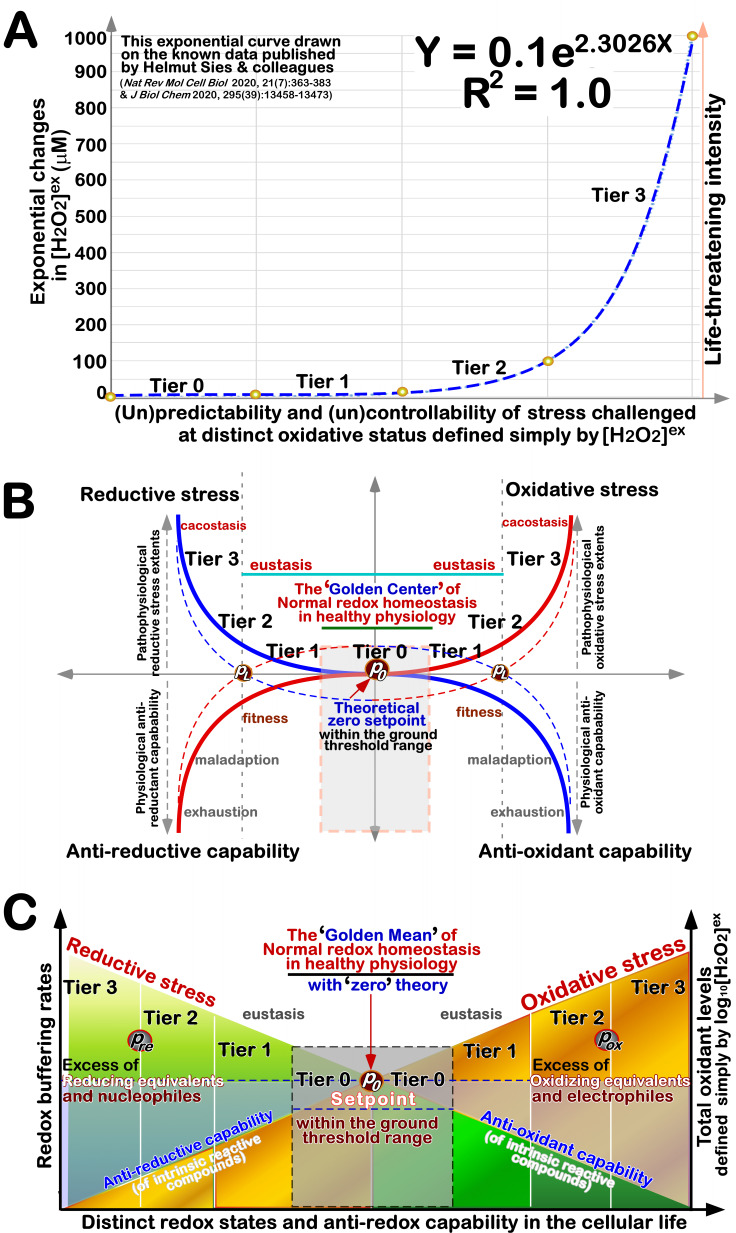
FIGURE 5: Quantitative stratification of redox stress and anti-redox responses into distinct output statuses. **(A)** The exponential curve was drawn from the data published by Sies and colleagues 64,169. The Y axle shows a positive correlation of exponential changes in [H_2_O_2_]^ex^ to the life-threatening intensity, while the X axle shows distinct oxidative statuses defined by [H_2_O_2_]^ex^, as indicated at Tier 0 to Tier 3, from predictability and controllability to unpredictability and uncontrollability. **(B)** The quadripartite curves are composed of oxidative stress and antioxidant capability, together with reductive stress and anti-reductive capability. In the Golden Center of normal physiological redox homeostasis 170, there exists a theoretical ‘zero’ setpoint (*P_0_*) within the homeodynamic threshold range. The eustasis for fitness principally takes place between Tier 1 and Tier 2, whereas cacostasis from maladaption to exhaustion occurs at Tire 3. **(C)** Simplification of the above-curved parameters by a base-10 logarithm. The Golden mean of normal redox homeostasis is demarcated for the healthy physiology with the ‘zero’ theory (at *P_0_*). Oxidative stress arises primarily from an excess of those oxidizing equivalents and electrophiles, whilst reductive stress results from excess of those reducing equivalents and nucleophiles. Yet, such redox stress and relevant defense responses occur, all of which also rely on the overall antioxidant and/or anti-reductive capability of intrinsic reactive compounds.

In 1977, the stress of life was simply qualified by Hans Selye 160, to be good eustress or bad distress, and also quantified as hyperstress (overstress) or hypostress (sustress); all four basic variations are deciphered in **Figure 4G**. Three decades later, Nel A. *et al*. presented a hierarchical redox stress model, in which Tiers 1 to 3 were first defined by distinct extents of oxidative stress with the inverse ratios of GSH/GSSG 166, but Tier 0 at the normal physiological conditions was left in a blank space. This blank space was filled by our work focused on Nrf1 167. Moreover, oxidative (and reductive) stress was classified by relevant dose-effect curves on the basis of its intensity and its time course 152,168. Oxidative eustress and distress were quantitatively distinguished by Helmut Sies and colleagues, based on extracellular and intracellular H_2_O_2_ concentrations (i.e., [H_2_O_2_]^ex^ and H_2_O_2_]^in^) 64 169 (as shown in **Figure 5A**).

### Updated hierarchical modelling of redox stress and anti-redox capability 

As illustrated in **Figure 5A**, a natural exponential curve was drawn from the data of [H_2_O_2_]^ex^ reported by Sies, H. *et al*. 64 169. The X-axle represents changes from predictability and controllability of eustress to unpredictability and uncontrollability of distress challenged at distinct oxidative status (Tiers 0 to 3), while the Y-axle shows the life threatening intensity of [H_2_O_2_]^ex^ (µM). On this base, according to the natural law of symmetry (as a fundamentally driving force), we thus conjecture that a robust steady-state redox system should be, at least in theory, demarcated by four dynamic curves, which represent oxidative stress, reductive stress, antioxidant capability and anti-reductive capability (of intrinsic reactive components), respectively (**Figure 5B**). In such parameter spaces, it is inferable there exists a highly conversed ‘habitable Goldilocks Zone’, in which all those essential redox and anti-redox biochemical elements should be tightly governed within certain homeodynamic threshold settings for its normal physiological hemostasis. The redox homeostasis could also be represented by a ‘Golden Center’ of healthy living systems, just as proposed by Ursini F. *et al*. 170. Equally importantly, a set point at approaching zero (*P_0_*) co-exists within this ‘Golden Mean’ for healthy living beings. This may be dubbed the ‘zero theory’, similar to that of a mathematic function, f(x) (± *C_R_*, a robust constant). Taken altogether, this conserved symmetry of redox stress and anti-redox responsive system closely adheres to Noether’s theorem (which states that the symmetry of their action corresponds to a conservation law, also with a conserved quantity).

The point of limit (*P_L_* ) is represented to allow for the resilience of this system to its normal archetype by proper responses to eustress, albeit this can lead to a novel eustasis for fitness 165. Conversely, further deterioration of redox distress could result in a server malfunction status of this response system (called cacostasis), with its maladaption or even exhaustion (**Figure 5B**). These can be reflected by more explicit **Figure 5C**, which was obtained from simple generalization by a base-10 logarithm of the above-curved parameters (e.g., log_10_[H_2_O_2_]^ex^), which is fully in accord with the Weber-Fechner law (*S* = *k* log_c _X). Moreover, it should be noted that those aberrant redox set-points (*P_ox_* or *P_re_*) may emerge during the malignant transition to cacostasis, and are monitored possibly by allostatic mechanisms 158 171.

### Differential involvement of Nrf1 and Nrf2 in redox stress responses stratified by physio-pathology

**Figure 6:**
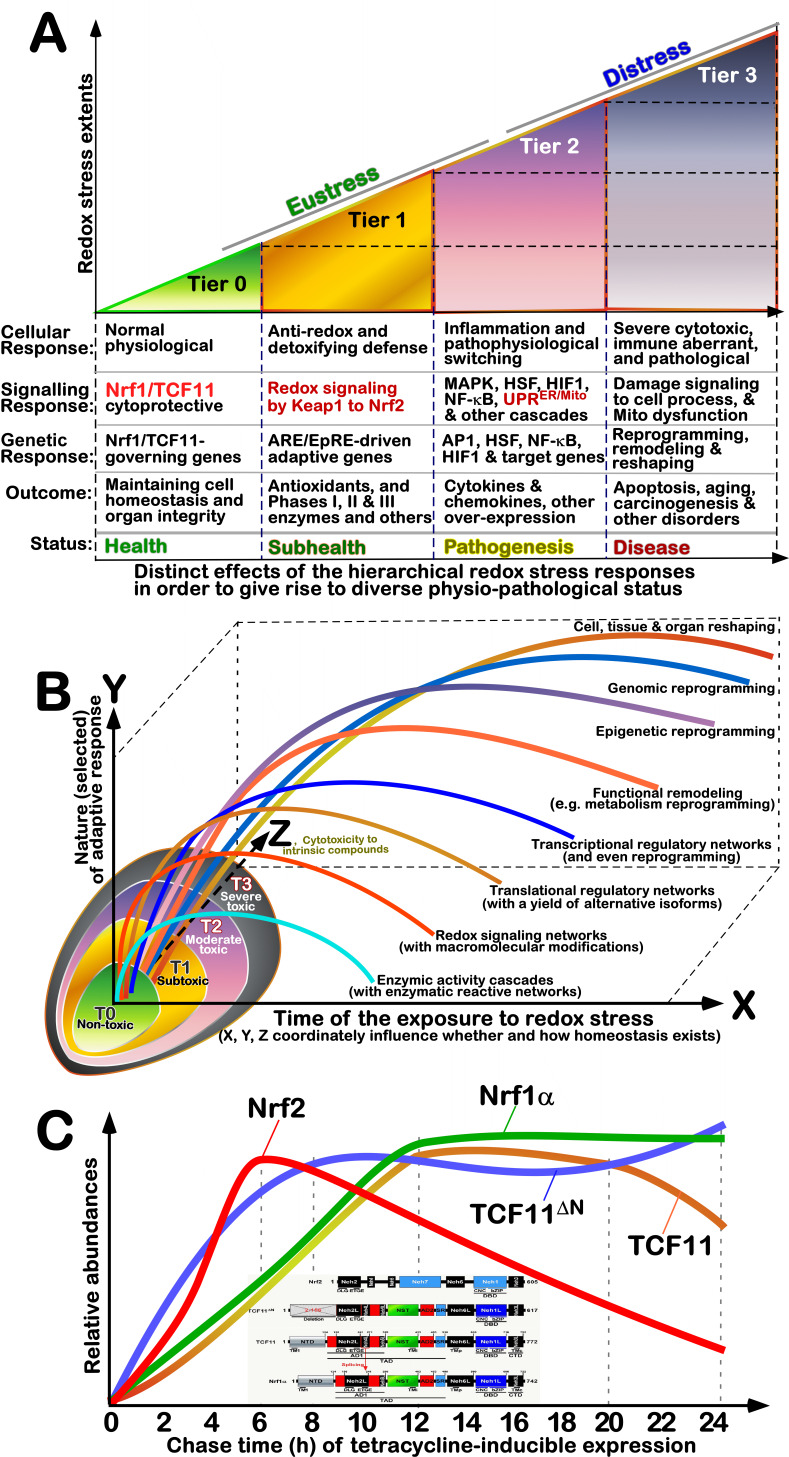
FIGURE 6: Definition of distinct hierarchical physio-pathological responses to distinct extents of redox stress. **(A) **Stratification of the physio-pathological defense responses to distinct extents of redox stress, with distinct progressive outputs established on the basis of previous multi-hierarchical models 166,167. Of note, distinct major roles of Nrf1 and Nrf2 are predominantly exerted to determine distinct statues defined at Tier 0 and Tier 1, respectively. **(B)** A three-dimensional graphic abstract of multi-hierarchical physio-pathological adaptive responsive curves (at Y axle) to distinct extents of cytotoxicity (at Z axle) arising from redox stress exposed for distinct lengths of time (at X axle). Overall, distinct physio-pathological statues are determined by these different X, Y and Z parameters. **(C)** Distinct time courses of stably expressing Nrf1α, TCF11, TCF11^ΔN^ and Nrf2 induced by a tetracycline-inducible HEK293 cell system (for detailed descriptions, please see the text and two cited references 123,178).

As shown in **Figure 6A**, we further refined distinct tiers of redox stress responses by being optimally stratified from their molecular, subcellular to cellular levels, which are established on the base of previous multi-hierarchical models 166 167. Of note, Tier 0 is defined under the normal physiological (healthy) status, which is predominantly dictated by Nrf1 (and its long isoform TCF11), together with its unique subset of target genes responsible for anti-redox, detoxifying and cytoprotective roles in maintaining cell homeostasis and organ integrity. This is just due to ever-accumulating facts that the loss of Nrf1/TCF11’s function results in severe endogenous oxidative stress 115,172,173; this is also accompanied by significant spontaneous pathological phenotypes as reviewed in detail 87 174. By contrast, loss of Nrf2 enables the resulting deficient cells and/or animals to become more susceptible and vulnerable to redox stressors or carcinogens, but not accompanied by any obvious spontaneous phenotypes 175. These demonstrate that Nrf1 is essential for CHR, whereas Nrf2 is not required for CHR, but still for CSR as defined by Kültz 124. At Tier 1, which is generally accepted as a redox eustress status with hormesis 166 167 176 177, the anti-redox, detoxifying and cytoprotective defense system is aroused by predominantly Keap1-sensing redox signaling to Nrf2-mediated target genes (driven by those cis-regulatory consensus antioxidant and/or electrophile response elements, called AREs/EpREs, within their promoter regions) in cellular responses to redox stress, particularly acute stress (**Figure 6A**). At Tier 2, is viewed as a major pathophysiological transition switching from a subhealthy status into the onset of certain pathology if redox stress has deteriorated, its responses become hence complicated. In addition to overstimulation of those (nonspecific) stress signaling networks responsible for activation of pathophysiological switching, the inflammatory responsive pathway mediated by NF-κB is critically activated and even hyper-activated, leading to the onset of certain pathologies and some pathogenesis of chronic diseases. When redox stress is further deteriorated from Tier 2 to Tier 3 (as a distressed status), severe cytotoxicity along with accumulating damage signaling to cell process, including mitochondrial dysfunction, leads to metabolic, epigenetic and genetic reprogramming, topogenetic remodeling and even cell and/or organ reshaping, with aberrant immunopathology. As consequence, this results in cell apoptosis and ageing-related chronic diseases, or otherwise aberrant proliferative carcinogenesis overstimulated by this distress. Collectively, the underlying molecular and cellular details in the response to distinct tiers of redox stress (**Figure 6B**) are required to be further elucidated. However, the major role of antioxidant and anti-reductive defense system (aiming to prevent, intercept, repair and eliminate) is fulfilled predominantly by redox-controlling enzymes, key transcription factors (e.g., Nrf1 and Nrf2) and co-factors involved in cellular redox biochemical and relevant metabolic processes, as well for a long-term adaption principally by reprogramming of their regulatory gene expression profiles in the physiological and pathophysiological responses to distinct severity of redox distress.

As shown in **Figure 6C**, stably expressed Nrf2 is rapidly induced within 2 h and then fast reaches to its maximal peak at 6 h of induction by a tetracycline-inducible HEK293 cell system (as described by 123 178), but thereafter gradually diminished and even extinguished. By sharp contrast, Nrf1α and its long isoform TCF11, along with its N-terminally-truncated isoform TCF11^ΔN^, are gradually induced by this tetracycline-inducible system to reach their maximal activation from 8 h to 12 h of induction and then maintained at so higher levels until experiments stopped. Additional two shorter Nrf1β and Nrf1γ (as a dominant negative form) are stably induced by tetracycline (Figure S1). Of note, both Nrf1α and TCF11 are required for N-linked glycosylation and then deglycosylation before being selectively processed to remove its N-terminal portion and thus give rise to a mature active CNC-bZIP factor, such as TCF11^ΔN^ (Figure S2A and C). All these distinct isoforms of Nrf1/TCF11, with differential half-lives of their protein stability (Figure S2), enable them to exert distinct or even opposing roles in the transcriptional regulation of their target genes 123 178. As such being the case, Nrf1/TCF11 is innately endowed to fulfill a unique indispensable function, that is distinctive from that exerted by Nrf2, in response to distinct tiers of redox stress. Hence, it is inferable that Nrf1α/TCF11 exerts an irreplaceable and pivotal role in governing CHR, whilst Nrf2’s role in handling such a rapid emergency response (CSR) has provided a way of ‘buying time’ for the transition to the lagging CHR mediated by Nrf1α/TCF11, insomuch as to coordinately cope with redox stress threatening cell homeostasis and organ integrity of living systems.

### Adaptive landscapes accounting for distinct responsive phenotypes to redox stress

**Figure 7:**
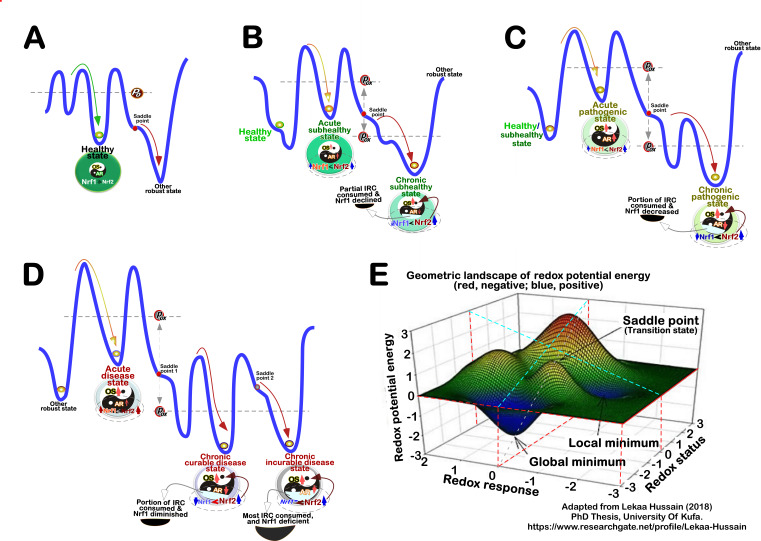
FIGURE 7: Adaptive landscapes account for distinct responsive phenotypes to redox stress. **(A) **The adaptive landscape at healthy redox state (dictated by Nrf1/TCF11), which is maintained within a homeodynamic threshold range (around the presenting *P_0_*), but can also be transformed into another robust state through saddle point. **(B)** The subthealthy adaptive landscape triggered by aberrantly augmented Nrf2 to yield a novel rise of setpoint at *P_ox_*. Its acute state is likely transited into another chronic state, while the intrinsic reactive compounds (IRC) were, at least in part, consumed along with Nrf1/TCF11 being declined by redox stress. **(C) **The pathogenically switching landscape was determined by redox stress-consumed IRC and decreased Nrf1/TCF11, leading to the onset of certain pathological phenotypes and even chronic pathogenesis. Of note, this cannot only be rescued, but conversely, further deteriorated by hyperactive Nrf2 under some conditions. **(D)** The pathological adaptive landscape is also likely transformed into chronic curable or even incurable disease states, at which the IRC was largely exhausted by severe redox stress, with the progressive loss of Nrf1’s function. **(E)** Geometric landscape of redox potential energy, with a strong correlation of redox adaptive responses to distinct redox stress statues (adapted from the model proposed by Lekaa Hussain 184).

Based on the mathematic models of Darwinian evolutionary laws and systems biology developed by Ao’s group 38 179 180 181, it is inferable that discrepant responsive physio-pathological phenotypes (and genotypes) to distinct tiers of redox stress should be determined predominantly by coordinated control of Nrf1/TCF11 and Nrf2, along with their differential but yet integral transcriptional regulation of cognate target genes responsible for anti-redox, detoxifying and cytoprotective defenses, as deciphered by distinct adaptive landscapes (**Figure 7A-D**). This is because distinct phenotypic (and/or genotypic) steady-states are dictated by different profiling of those key differential expression genes at the distinct status of each multi-hierarchical robust endogenous molecular-cellular network. Therein, differential expression of such a minimum set of key genes at distinct strata is de facto exhibited at their abundances, activities and topoforms at different topovectorial phase transitions, along with their intricate interactions between those core modular molecules in different subcellular, intracellular, and extracellular contexts.

A healthy redox homeostasis is maintained within a certain homeodynamic threshold range around P0 preset by Nrf1/TCF11-leading CHR to normal redox physiological fluctuations, in a robust basic redox metabolic network with cell respiration and aerobic energy metabolism (**Figure 7A**). According to the homeodynamic model (Figure S3) based on the theory for self-organization of dynamic systems by Lloyd D. *et al*. 182, homeostasis is only viewed as an exclusive mode of operation to emphasize the stability of the internal milieu toward perturbation, which is tightly governed by a series of feedback mechanisms controlling the normal physiological steady-state. That is to say, it is a relatively balanced (i.e., redox and anti-redox) stage of dynamics with monotonic states (fixed points). Since biological systems are indeed endowed with the dynamic capability to be self-referenced, self-organized and self-maintained in the eco-evo-devo process, they are placed as a steady state with high activity, particularly at certain phase transitions (of saddle points as a bistable redox switch at the excitable bifurcation, or contingently as a spontaneous oscillator with chaotic potentials so to lose their stability, but rapidly retake their resilience) 182 (**Figures 7A** and S3). Such processes could proceed on different spatiotemporal scales from those very rapid reactive processes between redox and anti-redox molecules within the membrane-compartmented spaces to other very slow evolutionary changes of, e.g., ‘redox-coded homeostasis’, in life histories 177 183. 

When the redox homeostasis is threatened by stress, the response of life experiences at least three phases: i) alarm reaction, ii) adaption with resistance, and iii) exhaustion leading to ‘general adaption syndrome’ 144. The time course of such redox adaptation was also classified into instant (emergency) response, short-term (metabolic) and long-term (transcriptional) adaptations 111 185. In addition to homeostatic feedback regulations, it is also required for multi-hierarchical homeorhetic regulations from molecular, and cellular to organismal strata (**Figure 6B**). Once a homeostatic loss of stability at the redox excitable bifurcation to a certain extent, the heterostasis and even allostasis mechanisms are switched to reset a new point of *P_ox_* or *P_re_* 186 187. The robustness of the homeostatic regulation is based on high-gain integral feedback mechanisms, while heterostasis could be associated with low-gain integral feedback processes, when organisms are submitted to the unitary-step disturbances or changes of the set-point (*P_ox_/P_re_*) for the novel feedback loop at the entrance of subhealthy eustasis or pathological cacostasis, which depend on whether such new *P_ox_/P_re_* are prescribed within an acceptable or even unacceptable ranges 188 (as shown in **Figure 7B-D**). Of note, the regulatory mechanisms for redox responsive adaptations are dependent on crucial roles of those key intrinsic reactive compounds (IRC), including important functional signaling molecules, metabolic enzymes, and transcription factors (e.g., Nrf1, Nrf2, HIF1, HSF and NF-κB) in reprogramming their critical signaling cascades, metabolism networks and target gene expression profiles insomuch as to dictate distinct redox physio-pathological phenotypes 64 65 87 88. If too much loss of homeostatic stability enables it to be gradually exhausted, so that its adaptive resilience is not fully recovered, or otherwise allostasis is overloaded, even to reach cacostasis, certain pathophysiological maladaptive responses are triggered, consequently leading to the putative pathogenic switches to chronic diseases, particularly when IRC is almost largely consumed or even exhausted, of which Nrf1 is diminished or abolished (as deciphered by adaptive landscapes in **Figure 7C-D**). Moreover, another explicit interpretation by a geometric landscape of redox potential energy coordinated with the redox responses to distinct redox states of different physio-pathological phenotypes is presented (in **Figure 7E**), as consistent with that model proposed by Lekaa Hussain 184.

## NRF1 IS A LIVING FOSSIL’S TRANSCRIPTION FACTOR, THAT IS CLOSER THAN NRF2 TO THE ANCIENT ORTHOLOGUES ARISING FROM THE ECO-EVO-DEVO PROCESS OF LIFE HISTORIES

Just for certain limitations existing in the past three decades, the origin of CNC-bZIP proteins seems to be only traced back to vertebrates, although their highly conserved CNC domain was first identified in the *Cnc* gene from *Drosophila melanogaster* 189 and thereafter unraveled in the *Skn-1* from *Caenorhabditis elegans* 190. Another noteworthy topic of such CNC-bZIP study is disproportionately focused on Nrf2 (also called NFE2L2), a stress-emergency responsive gene enabling to be rapidly examined by available experimental tools along with a necessary and powerful preference of its non-lethal knockout animals 87. The consequence was positively misleading to the putative state that the priority of Nrf2, together with its negative regulator Keap1 (Kelch-like ECH-associated protein 1), was evolutionally selected 191, so as to enable it to serve as a predominant and essential determinant responsible for antioxidant, detoxification and cytoprection against a variety of cellular stress 11 64 65 170 177 192 193 194. Nonetheless, such is not an accurately true case as a matter of objective facts that should be clarified hereby.

### Nrf1 is much closer than Nrf2 to the ancient orthologues emerged during evolution of life

**Figure 8:**
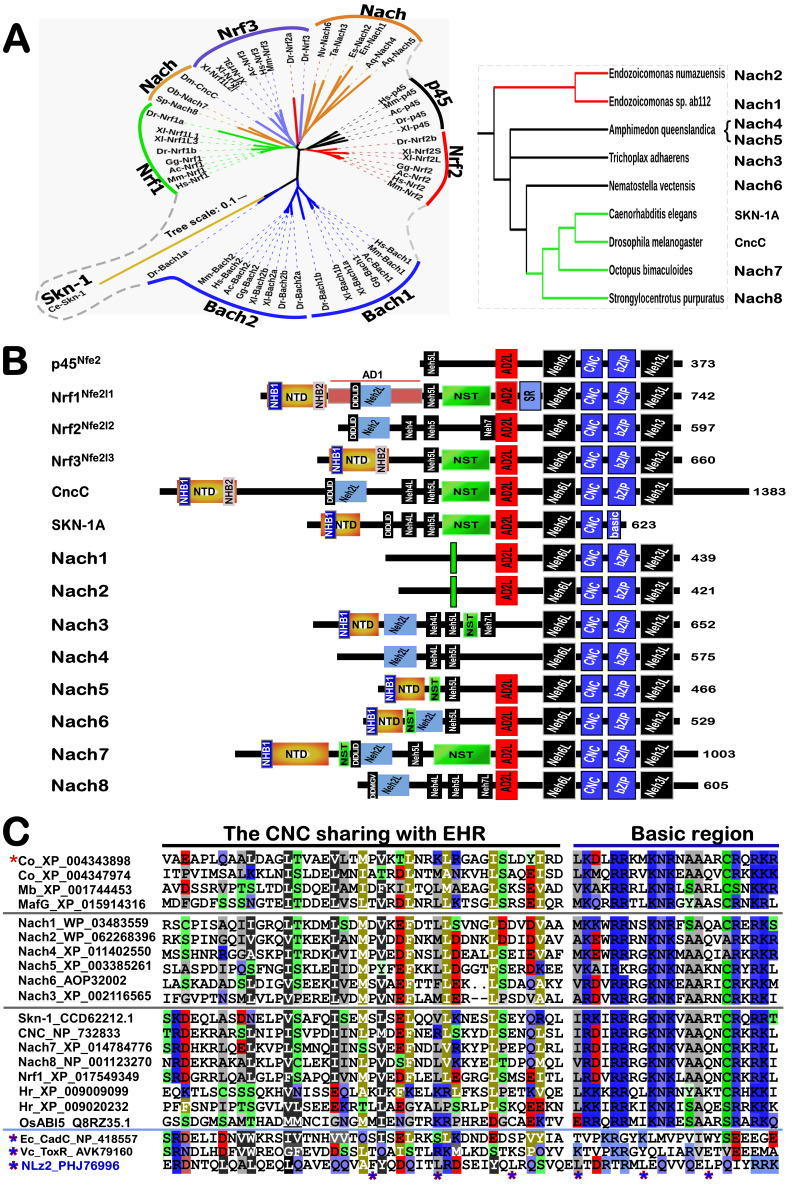
FIGURE 8: The living fossil-like Nrf1 is much closer than Nrf2 to their commonly shared ancient orthologues. **(A) **Two distinct models of the neighbor-joining phylogenetic tree of the CNC-bZIP family transcription factors, including Nach1 to Nach8, CncC, Skn-1, p45Nfe2, Nrf1, Nrf2 and Nrf3 from distinct species, as described elsewhere 200. **(B)** Schematic representation of distinct structural and functional domains of CNC-bZIP family members, along with some motifs indicated. For the detailed descriptions, please see the relevant text and another cited reference 87. **(C)** An alignment of multiple amino acid sequences within the DNA-binding CNC (conserved with the EHR)-adjoining basic regions from Nach1 to Nach8, CNC, Skn-1, Nrf1, MafG, and other putative homologous factors.

A neighbor-joining phylogenetic tree (**Figure 8A**) seems to be fan-shaped with three distinct clades and a bundle, to clarify the phylogenetic relationship of all selected CNC-bZIP family members by analyzing the evolutionary conservation of their amino acid sequences. Amongst them, a major clade is comprised of Nrf1 (also called NFE2L1, along with a long isoform TCF11 and another short form LCR-F1 or Nrf1β and), Nrf3 (also called NFE2L3) and Nach (i.e., Nrf and CNC homology) proteins from distinct metazoan species, which are much closer to their founding members Cnc and Skn-1 (**Figure 8A**, right panel). By contrast, Nrf2 and NFE2 p45 are closely clustered in the second clade, whilst the third clade is made up of the transcription repressors Bach1 and Bach2 (**Figure 8A**, left panel). These CNC-bZIP family members are also further gathered closer to both the sMaf/Maf and Jun subfamilies, within a relatively larger phylogenetic tree, that was constructed by distinct families of 495 bZIP proteins (Figure S4), as described previously in detail 114. This early-originated basic-region superfamily of transcription factors also includes those containing the basic helix-loop-helix zipper (bHLH-ZIP, e.g., SREBP1/2) or the bHLH-PAS domain (e.g., HIF1α). Such basic-region minimotifs, which were conjectured as a primordial peptide-replicated template 83, albeit with a rather lower fidelity, have been retaining divergently in most viral, bacterial and archaeal reigns, and all eukaryotic kingdoms of cellular life since the origin of their last common ancestor (LUCA, Figures S4 and S5). 

These CNC proteins except Skn-1 can heterodimerize with their partners sMaf 195 or other bZIP proteins (e.g., Jun, c-Fos, Fra1, ATF2, ATF4) 196 by physical interaction of their bZIP domains, before binding cognate target genes involved in antioxidant, detoxification and cytoprotection against oxidative and other stresses 197. Besides the bZIP region, the unique DNA-binding CNC domain is highly conserved in all the family members, including the *D. melanogaster* Cnc 198, *C. elegans* Skn-1 199 and other metazoan proteins Nach1 to Nach8 114, which emerged at the early evolutional stage of life histories (**Figure 8B**), but none of their orthologues are identified in plants and fungi. Of note, such a novel subgroup of Nach1-8 with high homology with all the vertebrate CNC-bZIP proteins (e.g., NFE2p45. Nrf1, Nrf2 and Nrf3) are identified to be predominantly present in the Echinodermata, Mollusca, Actiniaria, Placozoa, Porifera and bacteria, respectively (**Figure 8A** and **B**). Collectively, these findings demonstrate that the CNC-bZIP proteins are originated from the marine bacteria to the multicellular organism.

However, a perplexing gap appears to exist between the marine bacteria to the multicellular metazoa (e.g. *Amphimedon queenslandica*), because none of their orthologues have been identified in the unicellular protozoans. Further alignment of multiple amino acid sequences (**Figure 8C**) revealed that there still exists a conserved basic-adjoining region homologous with the CNC domain and another extended homology region (HER, within the Maf/sMaf family 201) in the filasterean bZIP protein (XP_004343898.1) from *Capsaspora owczarzaki* and also in the choanoflagellate bZIP protein (XP_001744453.1) from *Monosiga brevicollis*. Moreover, this is likely represented by a CNC-like Smc domain in the cyanobacteria bZIP protein (PHJ76996) from *Nostoc linckia z2* (**Figure 8C**, bottom line). Together with the marine bacterial Nach 1/2, these suggest that they were endowed with such an ancestral conservativity from the bacteria to the protozoa, to be commonly shared by all four families of CNC, Maf/sMaf, ATF2 and XBP1, albeit with certain variations. In addition, a bZIP protein (KRG21159.1) is yielded by *Candidatus Berkiella aquae*, a g-proteobacterium that exists as the obligate intranuclear endosymbiont of freshwater amoebae 202 [202]. This suggests a putative horizontal gene transfer (HGT) may occur during the protozoan evolution.

Of note, only one or two Nach proteins are found in each species such as ascidian, sea urchin, octopus, fly and hydra, with no exception of CNC/EHR-like bZIP proteins in the aforementioned protozoan organisms. This indicates that the expansion and diversification of the CNC-bZIP family seem to take place only in the vertebrate, leading to the yield of four to ten homologues encoded by distinct genes 114. Further comparison of structural domains in these CNC-bZIP proteins (as deciphered in **Figure 8B**) revealed that functional distinctions between Nrf1 and Nrf2 are dictated predominantly by an extra membrane-binding NTD (N-terminal domain, which contain an ER-targeting NHB1 signal peptide and another protease-processing NHB2 motif) and another N-glycosylated transactivation (NST) domain in Nrf1, but not in Nrf2 203. Such ER-associated NTDs are also present in Nach3, Nach5, Nach6, Nach7, CncC, Skn-1A and Nrf3, implying they should be endowed with similar topobiological features to those conferred upon Nrf1 87 119 120. By contrast, the stability of Nrf2 and its transcriptional activity are principally negatively regulated by Keap1, a redox-sensing adaptor targeting the former CNC-bZIP protein to the ubiquitin-mediated proteasomal degradation system 204. The negative regulation of Nrf2 by Keap1 is dictated primarily by physical interaction of the DGR domain of Keap1 with the N-terminal Neh2 domain of Nrf2 205 206. Similar Keap1-binding Neh2L domains are represented in Nach3, Nach4, Nach6, Nach7 and Nach8, as well in CncC and Nrf1, but not Skn-1 (**Figure 8B**). Yet, Keap1 is also absent in the sponges, placozoans, sea anemones or nematodes, although putative Keap1-like orthologues were found in some invertebrates, such as ascidian, sea urchin, octopus and fruit fly 207. 

Taken altogether, these demonstrate that Nrf1 rather than Nrf2 is much closer to its ancient orthologues (e.g., Nach, Skn-1 and Cnc) of this CNC-bZIP family emerged during the early evolution of life. This notion is also supported by the facts that distinct length isoforms of CNC or SKN-1, like Nrf1 are yielded from alternative splicing of various transcripts of their single genes, and ensuring alternative translation and post-translational processing of these proteins, an N-terminally-truncated isoform (as similar to Nrf1^ΔN^ or TCF11^ΔN^) of which functions as done by Nrf2 (**Figure 7B** and **C**), whilst their full-length isoforms (CncC and Skn-1A) evinced similar to the intact Nrf1α/TCF11 (**Figure 8B**). Therefore, it is inferable that the vertebrate Nrf2 is evolutionally selected by genetic duplication of a putative Nrf1-like gene that has been trimmed off the negative regulatory NTD, which enables it to rapidly mediate an efficient emergence response to stress and thus acquire a ‘buying time’ for the robust homeostatic response mediated by Nrf1/TCF11.

### Nrf1 is a living fossil of the CNC-bZIP transcription factors emerged in the redox eco-evo-devo process

**Figure 9:**
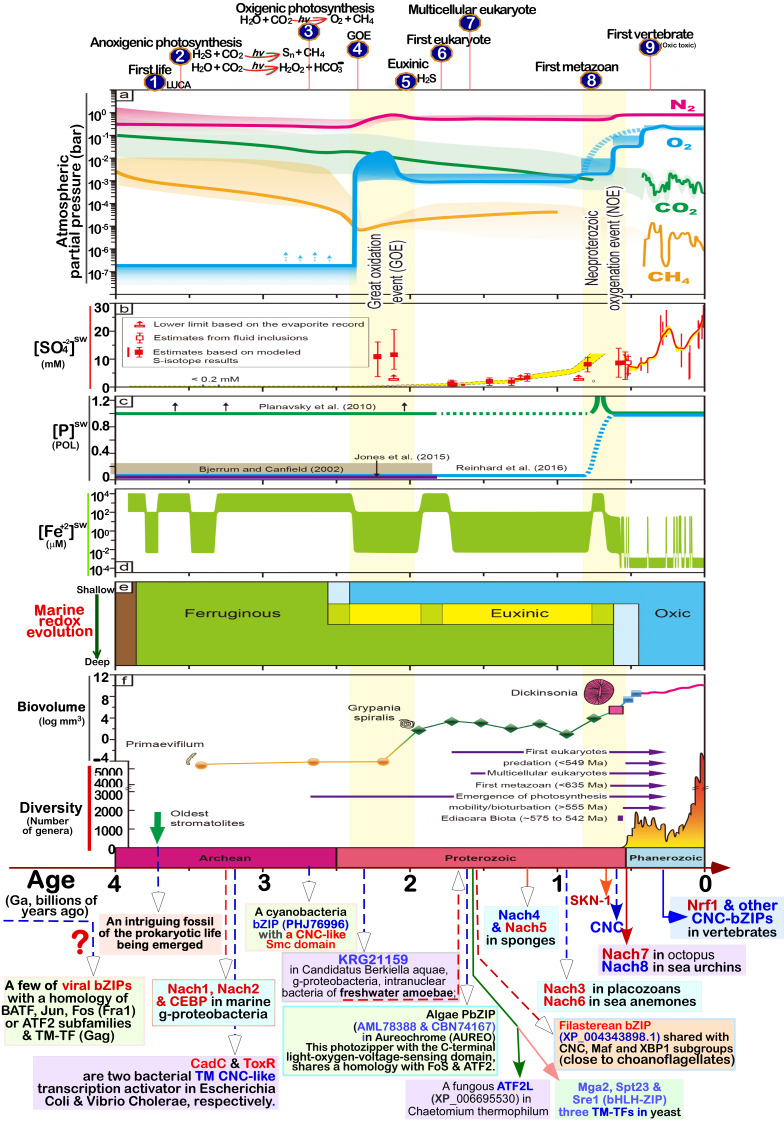
FIGURE 9: Co-evolution of life with redox atmosphere and oceans throughout the post-Archean (about 4.0 Ga to present). **(A) **Evolution of major constituents of the Earth’s atmosphere 208. Dinitrogen (N_2_, carmine line) may have tracked to the O_2_-levels due to an oxidative weathering and denitrification source of N_2_, but the changes in the partial pressure of nitrogen (pN_2_) are debated 209,210, as shown by a schematic of the speculative pN_2_ range in different time intervals consistent with very sparse proxy data (carmine shading). The O_2_ curve (blue line) shows a ‘classical, two-step’ view of atmospheric evolution 17,211: the first proposed O_2_ level of < 0.1% PAL between 1.8 and 0.8 Ga 40,212 and the second proposed O_2_-level of 10-40% PAL around 0.54 Ga 213. The median hydrogen carbon (CO_2_) with a 95% confidence interval (green lines) is from a carbonate-silicate climate model 214; this curve merges with a fit to CO_2_ proxy estimates from 0.42 Ga ago to present 215, also with various Precambrian pCO_2_ proxy estimates 216,217,218,219,220. A schematic history of methane (CH_4_) constraints (orange lines) is from a biogeochemical box model coupled to photochemistry 81, as consistent with possible biological CH_4_ fluxes into atmospheres of rising O_2_-levels at the GOE (Great Oxidation Event) and in the Neoproterozoic. The notion that such moderately high levels of methane may have contributed to greenhouse warming in the Proterozoic 221,222,223 has been disputed 224,225, which may depend on the fluxes from sources on land 226. The curve from ~0.4 Ga ago to the present is adapted from 227. **(B-D)** Evolution of marine sulfate concentrations [SO_4_^2-^] **(B) **estimated by data sources 228,229,230,231. Similar variations in the ocean phosphorus concentrations [P] **(C)** 232,233,234,235. Evolution of the deep-water iron concentrations [Fe^2+^] (**D**, below storm wave base) 236, with the lower limit of iron concentrations for Banded Iron Formation (BIF) and Marine Red Beds (MRB) being 50 μM and 4 nM, respectively. **(E)** Ocean redox conditions have evolved through time 237,238. The evolution of oxygenic photosynthesis permitted episodic accumulation of O_2_ (i.e., ‘oxygen oases’) along the late Archean ocean margins. Widespread surface ocean oxygenation accompanied the GOE. Elevated rates of microbial sulfide production at sites of a high organic carbon export enabled the development of euxinic conditions at mid-depths (and in restricted basins). The deep oceans remained predominantly ferruginous 239. The expansion of ferruginous deep oceans is thought to have occurred during a major crustal growth event at 1.88 Ga and during the severe Neoproterozoic glaciations. Widespread ocean oxygenation commenced by the Ediacaran Period 82,240,241. Yet, significant oscillations in ocean redox conditions may have occurred until a Devonian oxygenation event (DOE) led to permanent ventilation of the oceans (except for sporadic and transient oceanic anoxic events). **(F)** The evolutionary history of life on the Earth. The earliest records of life (in oldest stromatolites) are as old as ~3.7 to 4.1 Ga 242,243. Oxygenic photosynthesis must be present before the GOE, either directly before the GOE 244 or as early as 3.0 Ga 40,55. The earliest convincing evidence for eukaryotic life is present at ~1.7 to 1.6 Ga 245, albeit the origin of eukaryotes could have been much earlier 246. Records of the multicellular eukaryotes appear as early as ~1.2 to 1.6 Ga 247,248. The first appearance of animals, as indicated by the biomarker evidence for early sponges, occurs during the Cyrogenian ‘Snowball Earth’ episode 249. The Neoproterozoic Oxygenation Event (NOE) possibly unleashed major biological innovations, including the appearance of new biological and ecological strategies: the first metazoans 249,250, the appearance of the first large and architecturally complex organisms during the Ediacaran 251, the Cambrian Explosion with subsequent rapid increases in diversity of species (yellow-to-red shaded area) 252,253, the emergence of mobility and predation 254,255. The upper logarithmic curve shows the increase of the maximum size of organisms throughout the Earth’s history, such as prokaryotes (orange dots), protists (dark green diamonds), vendobiont (probable multicellular eukaryotes, e.g. Dickinsonia, carmine square), animals (blue squares), vascular plants (green line), which was modified after Payne *et al*., 2009 256. Of note, all annotations on the bottom were based on Supplemental Figure S6, and interpreted in the text.

From the origin of life and its co-evolution with the surrounding atmosphere (consisting of N_2_, O_2_, CO_2_, CH_4_) and ocean (SO_4_^-2^, P and Fe^+2^-containing) redox environments of life histories to give rise to diversity of the modern biomes on Earth was deciphered schematically in **Figure 9**. At least nine of the major historical events of life had occurred (as indicated on the top) throughout the entire eco-evo-devo process, nearly seven-eighths of which were dominantly placed on the sulfur-based reductive environments, whereas only the recent one-eighth of life histories was principally based on the oxygen-centered oxidative environments 37 48. In such distinct challenging conditions, the intracellular redox homeostasis of almost all life forms has steadily been maintained within a homeodynamic threshold range by evolving their intrinsic anti-redox responsive mechanisms (CHR, CSR, OHR and OSR), which are differentially monitored by the CNC-bZIP family transcription factors, as described above (in **Figures 3, 4B** and **6A**).

#### Co-evolutionary histories of life with its ambient redox environments

Redox evolution of the Earth’s atmosphere environments 208 was dependent on changes in the major constituents of small molecular gases, such as H_2_/H_2_S, NO/N_2_, O_2_/H_2_O_2_, and CO_2_/CH_4_. Of note, only one-eighth of life histories nearly to the present had relied on the oxygen-based redox ambient atmosphere, as shown by the O_2_ curve (**Figure 9A**). A ‘classical, two-step’ view of atmospheric evolution 17 211 revealed the first proposed O_2_ level of < 0.1% PAL between 1.8 and 0.8 Ga 40 212 and the second proposed O_2_ level of 10-40% PAL around 0.54 Ga 213. Another curve merges with a fit to CO**2** proxy estimates from 0.42 Ga ago to the present 215, also with various Precambrian pCO_2_ proxy estimates 216 217 218 219 220. A schematic history of methane (CH_4_) is from a biogeochemical box model coupled to photochemistry 81, as consistent with possible biological CH_4_ fluxes into atmospheres of rising O_2_ levels at the GOE and in the Neoproterozoic oxygenation event (NOE). By contrast, the prior seven-eighths of life histories relied on sulfur-based redox environment, with early energy metabolism from the H_2_S/FeS and respiratory NO pathways 23 37 105. The NO is produced primarily by successively cascaded reduction by the NO_3_^-^ → NO_2_^-^→ NO pathway, which is reduced to yield N_2_O, the latter continuously reduced to generate N_2_ (as shown in **Figure 2**).

The origin of life and its ensuing evolution was de facto dictated by evolving the ocean redox environments on the Earth 237 238. The beginning of life was recorded in the oldest stromatolites (**Figure 9F**) from ~3.7 to 4.1 Ga ago 242 243. That is, the origin of life was placed in an anoxic (no O_2_) and reducing (H_2_/H_2_S) environments, and also dominated by ferrous iron (Fe^+2^/FeS) in the ferruginous oceans, demonstrating that the first organisms (i.e., LUCA) were likely sulfur-metabolizing. About 200 million years later, anoxygenic photosynthesis appeared allowing organisms to escape from a chemotrophic existence by using H_2_S to reduce CO_2_ to yield organic carbon (e.g., CH_4_) while oxidizing sulfur to polysulfide (S_n_) (**Figure 9**, on the top). In this anoxygenic process CO_2_ may be partially reduced to HCO_3_^-^ along with H_2_O being oxidized to yield minor H_2_O_2_. Around 3.0 Ga when the efficiency of light-gathering antennae increased, H_2_O is sufficiently used as the reductant to generate O_2_ in oxygenic photosynthesis (appeared firstly in cyanobacteria). The evolution of oxygenic photosynthesis permitted an episodic accumulation of O_2_ (i.e., ‘oxygen oases’) along the late Archean ocean margins. Widespread surface ocean oxygenation accompanied the GOE in a slight increase of atmospheric O_2_ levels. Such slightly increased O_2_ in the atmospheric conditions enabled sulfur to be oxidized to sulfate (SO_4_^-2^) that was then washed to sea, in which this sulfur was also reduced insomuch as to form massive euxinic oceanic areas that were anoxic and sulfidic (H_2_S/RS) (**Figure 9E**). The elevated rates of microbial sulfide production at sites of a high organic carbon export enabled further development of euxinic conditions at mid-depths (and in the restricted basins), but the deep oceans remained predominantly ferruginous 239. Expansion of the ferruginous deep oceans is thought to have occurred during a major crustal growth event at 1.88 Ga and during the severe Neoproterozoic glaciations. Then widespread ocean oxygenation commenced by the Ediacaran Period 82 240 241. Yet, significant oscillations in the oceanic redox- stratified conditions may have occurred until a Devonian oxygenation event (DOE) led to permanent ventilation of the oceans, except for sporadic and transient oceanic anoxic events 82. 

From the evolutionary history of life (**Figure 9F**), oxygenic photosynthesis must be present directly the GOE 244 or as early as 3.0 Ga 40 55. The earliest convincing evidence for eukaryotic life appeared in the euxinic environments at ~1.6 to 1.7 Ga 245 and shortly thereafter plants also emerged, albeit the origin of eukaryotes could have been much earlier 246. The records of multicellular eukaryotes appear as early as ~1.2 to 1.6 Ga 247 248. The first appearance of animals, as indicated by the biomarker evidence for early sponges, occurs during the Cyrogenian ‘Snowball Earth’ episode 249. The NOE occurred when a considerable amount of O_2_ was produced predominantly by plants, besides cyanobacteria, gradually rising to present-day levels, allowing the ocean to be sufficiently oxidized, such that the sulfide disappeared, before major biological innovations were unleashed. Those include the appearance of new biological and ecological strategies: the first metazoans 249 250, the first large and architecturally complex organisms during the Ediacaran 251, the Cambrian Explosion with subsequent rapid increases in diversity of species 252,253, along with the emergence of mobility and predation 254 255.

#### Nrf1 is a living fossil of the CNC-bZIP factors arising from distinct evolutionary stages of life

**Figure 10:**
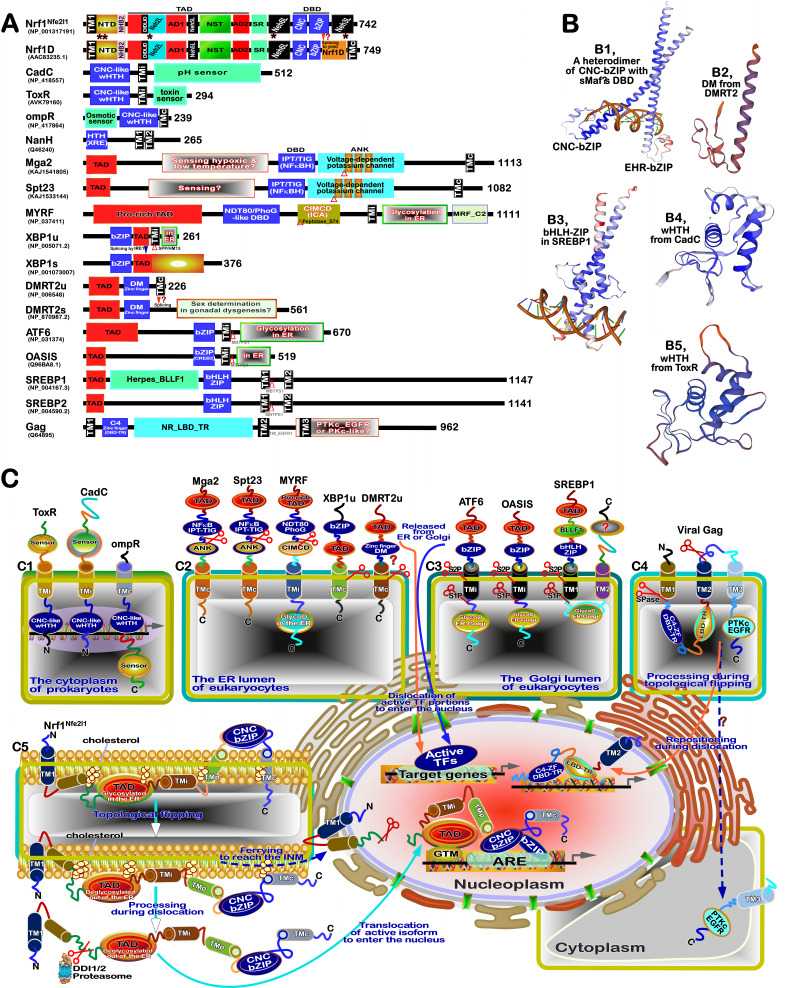
FIGURE 10: Unique topogenetic folding of Nrf1 and its dynamic dislocating across the ER membranes to the nucleus. **(A) **A set of transmembrane transcription factors selected from all distinct kingdoms of life on the earth. These life species include prokaryotes (bacteria), and unicellular and multicellular eukaryotes (yeasts, algae, plants and complicated animals). Most of their functional domains of Nrf1, Nrf1D and others have been identified (in the relevant references) except the viral Gag. **(B)** Several highly conserved structures of the DNA-binding domains (e.g., CNC-bZIP, bHLH-ZIP) from those indicated key transcription factors, which were adapted from αFoldDB (https://www.uniprot.org/). **(C)** By membrane-topogenetic folding of the heretofore identified transmembrane transcription factors, they are divided into five distinct classes. Of note, Nrf1 is distinctive from other factors, because it is endowed with unique membrane-topogenetic folding and dynamic repositioning across membranes, before selective proteolytic processing of this CNC-bZIP factor and then dislocating from the ER to the nucleus, before regulating cognate genes.

All those CNC-bZIP and CNC-like transcription factors existing in distinct species, so far as we have known, were marked according to the evolutionary tree of life (Figure S6, adapted from  https://evogeneao.s3.amazonaws.com/images/tree_of_life/tree-of-life_2000.png ). The possible emergence of distinct evolutionary stages of life histories was markedly associated with the ecological co-evolution of their relevant ambient environments (**Figure 9**, on the bottom). Since the oldest stromatolites are an intriguing fissile of the prokaryotic life being first emerged from ~3.7 to 4.1 Ga ago 242 243, the facts that both Nach1 and Nach2, along with ancient CEBP were found in the marine g-proteobacteria 114, and their CNC-bZIP domains are highly conserved closer to those early originated animal Nach4, Nach5 (both in sponges), Nach6 (in anemones) and Nach3 (in placozoans) (**Figure 8C**) demonstrates that the CNC-bZIP family of transcription factors is at least early originated from the marine bacteria (**Figure 9**, on the bottom). Further, the vertebrate Nrf1 rather than Nrf2 or other homologues is evolutionarily conserved closer to the metazoan CNC-bZIP factors, such as Skn-1 (with no Keap1 in nematodes), CNC (in fruit flies), Nach7 (in octopus) and Nach8 (in sea urchin) (**Figures 8** and **9**). In addition, it is important to note that two bacterial transmembrane CNC-like transcription activators CadC and ToxR 257 258 were found in *Escherichia coli* and *Vibrio cholerae*, respectively (**Figures 8C** and **10**). Taken together, these demonstrate that the transmembrane-associated Nrf1 is a living fossil-like CNC-bZIP factor, that is much closer to the above-mentioned ancient orthologues arising from distinct stages of life evolutionary histories, when compared with the water-soluble Nrf2 factor.

From the evo-evo-devo perspective, the euxinic oceanic reductive environments (H_2_S/RSS without O_2_) leads to the appearance of the first eukaryotes, i.e., unicellular protozoans, albeit after the GOE aroused from oxygenic photosynthesis in cyanobacteria (with CNC-like Smc domain-containing bZIP protein, PHJ76996). Coincidentally, two protozoan CNC/HER-like bZIP proteins (i.e., XP_004343898.1 and XP_001744453.1) were found to exist in the filasterean *Capsaspora owczarzaki* and the choanoflagellate *Monosiga brevicollis*, respectively, and also highly conserved with the CNC, Maf/sMaf, ATF2 and XBP1 families (**Figure 8C**). According to the endosymbiotic theories for eukaryote origin 259 260, the naissance of the chimeric eukaryote was accompanied by origin of the nucleus from the karyomastigont in amitochondriate protists. This notion is further supported by an obligate intranuclear endosymbiont of a g-proteobacterium in the freshwater amoebae *Candidatus Berkiella aquae* (encoding a bZIP protein, KRG21159.1) 202. Thereby, it is postulated that bacterial CNC/bZIP-encoding genes were allowed for the putative HGT to be retained in the earliest eukaryotes (i.e., the unicellular protists) and further differentiated to yield different ancient progenitors sharing with distinct families of versatile bZIP transcription factors emerged in the subsequently evolving protozoans, and even in the multicellular metazoans.

It is of equal importance to notice that the transmembrane CNC-bZIP transcription factors (Nach5) emerged as accompanied by the first appearance of animals, as indicated by the biomarker evidence for the early sponges 249. Similar ancient transmembrane-bound orthologues Nach3, Nach6 and Nach7 are present in placozoans, anemones and octopus, respectively, alongside CncC and Skn-1 (**Figure 8**). Besides, two additional bacterial transmembrane CNC-like transcription activators CadC and ToxR 257 258 were also found in *E. coli* and *V. cholerae*, respectively. Hence, it is inferable that a putative fusion gene encoding a transmembrane-associated polypeptide fused with another portion of CNC-bZIP protein is much likely generated by homologous recombination of an endosymbiont transmembrane CNC-like gene with another host CNC-bZIP gene and efficiently expressed during the origin of multicellular metazoans. The appearance of such a membrane-tethered CNC-bZIP factor enables the ancient animals to require adaptation to the H_2_S/RSS-based euxinic oceanic environments. Thereafter, the expansion and diversification of this CNC-bZIP family took place only in the vertebrate, leading to a yield of 6 to 10 homologues (e.g., Nrf1, Nrf2, Nrf3, p45NFE2, Bach1 and Bach2) encoded by distinct genes that had been duplicated and also differentiated during evolution 114. This is contributable to an efficient response to the NOE caused primarily by plant photosynthesis, which leads to the gradual appearance of the first large and architecturally complex organisms (e.g., vertebrates) and subsequent rapid increases in the diversity of life species 251 252 253. 

Overall, the transmembrane-bound Nrf1, rather than water-soluble Nrf2, is a living fossil-like CNC-bZIP factor that is highly conserved with the aforementioned ancient orthologous members emerging at distinct stages of life evolutionary histories. Such objective facts demonstrate that Nrf1 is innately endowed to fulfill a subset of certain unique, intrinsic and indispensable biophysiological functions, which are distinctive from those done by Nrf2, at maintaining cell homeostasis and organ integrity 87. Conversely, loss of Nrf1’s function is inevitable to resulting in severe redox stress and pathological phenotypes 87 174. For example, the conditional knockout of Nrf1 in mouse livers leads to spontaneous non-alcoholic steatohepatitis (NASH) and subsequent malignant transformation into hepatoma 261 262. The full-length Nrf1α-specific knockout by two distinct gene-editing techniques in human HepaG2 cells leads to several redox stress and lipid deposition, along with metaflammation 263 264 and cancer malignance 265 266. Collectively, these indicate that Nrf1, particularly Nrf1α/TCF11, is intrinsically conferred to act as a potent cancer-repressor 123 167.

#### Cancer is likely defined as ‘oncoprotists’ by the eco-evo-devo ontology of its origin 

From an integrated eco-evo-devo perspective 267 268 269, cancer could be defined by the ‘nature scientific essence of cancer’ to selectively survive as an atavistically specialized unicellular life form (like protists) originated from the host tissues comprising collective multicellular sets in a harmonious cooperation with distinct types of cell lineages. Generally, cancer is early originated from an oncogenically-specialized cell, that was derailed from the integrated multicellular collective controlling system setting for the host tissue homeostasis and had also undergone adaptive selection pressure by laws of Darwinian evolutionary dynamics under ecological conditions of redox stress damage and/or long-term inflammatory infection within the host tissues, particularly upon the loss of Nrf1. Ultimately, it turned out that such a specialized oncogenic cell-proliferating population has undergone an exclusive continuum of the retrogressive and degenerative process by reprogramming of cell metabolisms, genetics, epigenetics and topogenetics during intragenerational proliferation insomuch as to return the original status of its unicellular life. This is, in ontological essence, viewed as a single-cellular protozoan, hence designated as ‘oncoprotists’, some with primitive characteristics of polyploid giant cancer cells (PGCC) 270. This notion has been already evidenced by experimentally establishing almost all cancer unicellular lines and all relevant tumor-transplanted xenograft models. From this view, it is inferable that the most critical core of scientific questions in the current cancer research is how to decode the origin and evolution of oncoprotists (i.e., cancer life); that is, *nothing in this core makes sense except in the light of cancer biology*. Thereby, there is deduced to exist a vital key for cancer prevention strategy from dedifferentiation of host cells (N →1) to generate an ‘offbeat’ oncogenic organism (i.e., oncoprotist) and in the meantime, how to induce the oncoprotists to be redifferentiated further to switch onto the right normal physiological paths (1→ N).

## UNIQUE TOPOGENETICS OF NRF1 AND ITS DYNAMIC DISLOCATION ACROSS MEMBRANES TO THE NUCLEUS BEFORE REGULATING TARGET GENES

The basic concepts of topogenetics about membrane heredity, membrane system platform and relevant coding rules in topobiology have been introduced above (in Section "Redox kinetic barriers dictated by membrane topogenesis (via 'topogenetic code')"). Briefly, the early origin of almost all cellular life forms was dictated predominantly by the topogenesis of the most primordial membrane platforms and subsequent evolving membrane systems with inheritance. These cellular membrane-based frameworks are multi-hierarchically cross-linked with intracellular and extracellular matrix-scaffolding networks to selectively self-assembly an adaptive ‘toposkeleton’ preset for the body plan to self-organize a topoform-specific life. Thereby, the membrane system platform is essentially constructed for laying a solid foundation of the origin of life and ensuing evolution. Such membrane-relevant topogenetics should be tightly governed by key transmembrane-molecular machinery, of which critically membrane-bound transcription factors are encompassed in order, to serve especially as essential controls of the topogenetic transformation, e.g., between the epithelial and mesenchymal status of cellular life.

### The peptide minimotifs evolving from primitive templates to specific DNA-binding transcription factors

According to the Central Dogma (i.e., DANN→ RNA→ Protein), the diversity of almost all cellular life’s identifications and behaviors, with a vast variety of topoforms and relevant physio-pathological status should be predominantly determined by this dogma-governed gene expression profiling. Such gene-centered determinism dominating life sciences for over 70 years seems to be almost not questioned. Although the genetic codes for Central Dogma were selected by Nature during a long-term eco-evo-devo process, it is impossible that the first formation of such DNA encoding genes was de facto made in the primordial worlds (e.g., ‘chemical world, metabolic world, lipid world, peptide world, and RNA world’), all of which were likely to be included within the membrane-based vesicles at the early beginning of cellular life. Hence, it was conjectured that the primeval replicating templates in the reproducing pre-cells or proto-cells are likely executed by those living fossil-like minimotifs (e.g., ‘basic-region zipper’) 83 84 85, albeit with low infidelity (to yield multiple paraorthologous polypeptides by direct self-replication). Besides, the natural selection from such genetic minimotifs of peptides can also enable the ‘reverse translation’ to give rise to the first single-stranded nucleotides (RNAs and/or DNAs) of life’s origin 271. Such a reversed flow of the genetic information from the peptide minimotifs as templates to yield the primitive RNA/DNA (i.e., peptide → RNA/DNA), alongside another flow of known genetic information from RNA to DNA or topological folding chromatin 272, should thus collectively be referred to as the ‘*Reverse Central Dogma*’, an obligate rule that is de facto followed in the course of prion propagation 273, although not central to biology. Moreover, the route of information transfer from protein to the genome might not be completely blocked; that is, so formally, the strict validity of the Central Dogma could be questioned 272, possibly by the convergence of foundational discoveries from synthetic biology (e.g., of centriole).

Conversely, in the ensuing molecular evolutionary process, a house of such fissile-like minimotifs-containing polypeptides (e.g., the ‘basic-region zipper’ superfamily) were ultimately selected as distinct sets of specific DNA binding transcription factors, which are innately endowed to fulfill essential functions in controlling their cognate genes. Of note, a portion of such *trans*-acting transcription factors was further subjected to distinct topogentic folding to be integrated and positioned within and around the membrane-based platforms (**Figure 10**).

### Distinct topological folding of transmembrane transcription factors existing in all kingdoms of life

Such membrane-spanning transcription factors were predicted computationally to exist in all kingdoms of life, and even in viruses 274. Amongst them, transmembrane-associated CNC-ZIP transcription factors (Nrf1, Nrf1D, Nrf3, Skn-1A, CncC, Nach3, Nach5, Nach6, and Nach7 in **Figure 7B**) were identified by their conserved NHB1 signal sequences targeting the ER and anchoring the ER membranes 114 203 275. In bacteria, a group of membrane-spanning transcription factors (e.g., CadC, ToxR, ompR) with distinct sensors for pH, toxin, osmotic changes across the membranes (**Figure 10A**) were identified to regulate target genes by directly DNA-binding wHTH (winged helix-turn-helix) domains 257 258, which seems to be topologically folded as similar to that of the CNC-like domain (**Figure 10B**). One of the extensively studied pH-responsive systems is CadC, which is a genetic transmembrane one-component sensor for dynamic regulation of D-xylonic acid accumulation 276. The monotonic membrane-spanning ToxR regulon is also linked to the lipid-remodeling gene expression profile in *V. cholerae* 277; its life cycle is controlled by regulated proteolysis of ToxR allowing rapid adaptation to stress 278. NanH was identified to act as a bitonic membrane-spanning HTH transcription regulator that binds the promoters of genes involved in sialic acid metabolism of the anaerobic pathogen *Clostridium perfringens* 279 280, in which the sialidase mediates the early-life colonization by a pioneering gut commensal 281. This is just attributable to the fact that NanH facilitates commensal resilience and recovery after antibiotic treatment in a defined microbial community, revealing a co-evolutionary mechanism with microbiota by the host-derived glycans to promote stable colonization. Furthermore, by genetically dissecting the functions of regulatory proteins and enzymes responsible for catechol metabolism in the human gut bacterium *Eggerthella lenta*, another widespread family of the polytonic membrane-spanning LuxR-type transcriptional regulators was also revealed to be topologically folded, as similar to DadR, HcdR and CadR 282.

In yeasts, two homologous transmembrane NF-κB-related transcription factors Spt23 and Mga2 are processed selectively in proximity to the ER by an internal proteasomal cleavage within the ANK repeat domains (retaining a voltage-dependent potassium channel) to be released as mature trans-activators 283 (**Figure 10B** and **C2**). Both factors play distinctive roles in the activation of the Δ9 fatty acid desaturase gene *OLE1* (crucial for *de novo* biosynthesis of unsaturated fatty acids as lipid building blocks) in *Saccharomyces cerevisiae* 284, but the fatty acid-mediated regulation of the Mga2 activity is independent of its proteolytic processing to yield a soluble transcription activator. Of note, Mga2p was originally identified as the first eukaryotic sensor for low temperature and oxygen to induce the *OLE1* expression 285 through the low oxygen response elements (LOREs) in their promoter regions, but this gene is repressed by unsaturated fatty acids 286. The OLE pathway is generally accepted as the best-characterized, eukaryotic sense-and-control system to regulate the membrane lipid saturation by Spt23 and Mga2 287, two key factors to determine the maintenance of a fluid lipid bilayer as membrane integrity and even cell viability. This is just based on the fact that such membrane fluidity and phase behavior are dictated by a proper proportion of both saturated and unsaturated acyl chains in the composition of membrane lipids.

In almost all eukaryotes from yeast to humans, another link exists between the ER membrane lipid sensing by a juxta-membrane amphipathic helix within the transmembrane domain of inositol requiring enzyme 1 (IRE1, acting as both kinase and endoribonuclease) and the resultant IRE1 signaling activation by detecting lipid bilayer stress to trigger the unfolded protein response (UPR) mediated by XBP1 (along with its homologous HAC1 in yeasts and bZIP60 in plants) 288. This fissile-like IRE1-XBP1-UPR axis 289 is induced by lipid disequilibrium to counteract membrane stress-induced cell death by reprogramming protein homeostasis and maintaining membrane functions without affecting its composition 290. For this, the expression of different responsive genes to lipid bilayer stress and/or unfolded protein stress is regulated by spliced XBP1/HAC1/bZIP60 through the IRE1 sensor 291. However, the unspliced XBP1u mRNA and its protein are targeted to the ER membrane 292 293 294. Under normal conditions, the prototypic XBP1u protein is anchored within membranes through its C-terminal transmembrane (TMc) region (**Figure 10C2**), in a topological fashion similar to the C-terminal Nrf1D, before eliciting its unique function as a transcriptional repressor against its spliced effects 114. Upon exposure to ER stress, the splicing of XBP1u mRNA transcripts by IRE1 to remove 26 nucleotides gives rise to an open reading frame-shifting variant XBP1s (**Figure 10B**), which lacks an available TM-targeting peptide to directly translocate the nucleus and regulate target genes involved in the UPR 289. Similarly, the TMc peptide of prototypic DMRT2u (of 226 aa) enables it to anchor within membranes (**Figure 10C2**), but alternative splicing of its transcripts in the stop codon-containing exon 4 gives rise to a fusion protein (DMRT2s) with an additional 328-aa polypeptide sharing 58% identity with *Terra* critical for somitogenesis and sex determination 295. 

Another classic ER stress signaling axis to UPR is monitored by the TM sensor ATF6, which is folded to adapt its initial membrane-topology within and around the ER (**Figure 10C3**). If stimulated by ER stress, it is transported to the Golgi apparatus, in which this bZIP protein is allowed for the progressive two-step process by Site-1 and Site-2 proteases (i.e., S1P and S2P) to yield a cleaved ATF6n factor 296 297. Then, ATF6n is released to enter the nucleus and activate target genes driven by either UPR elements or ESRE (ER stress response element) within their promoter regions. Similar topological folding and processing also take place in the case of OASIS (**Figure 10C3**). By contrast, although sterol regulatory element binding protein 1 (SREBP1) contains two TM domains with distinct local topologies integrated within and around the ER, only its TM1 is folded in a similar orientation to that of ATF6. When its target genes are required for cholesterol and other lipid synthesis, SREBP1 is allowed for a similar transfer to ATF6 and then dislocated from the ER through the Golgi to the nucleus (**Figure 10C3**). This processing is attributed to intramembrane proteolysis by S1P and S2P, successively, in the Golgi apparatus so to generate a cleaved activator SREBP1n 297 298. Additional evolutionarily conserved TM-bound transcription factor MYRF (myelin regulatory factor) was identified in invertebrates to vertebrates, which is subject to proteolytic self-processing into an active factor required for myelin development and relevant diseases 299 300 301 302. Overall, the intracellular redox, protein and lipid homeostasis all are robustly maintained and thereby tightly governed by all aforementioned transmembrane proteins together with other canonical sensors and transducers increasing during evolution from yeasts to vertebrates in the response to distinct types of stresses.

### Unique topological processing of Nrf1 to yield an active CNC-bZIP factor along with short isoforms

The unique membrane-topogenetic folding of Nrf1, its post-synthetic modifying and proteolytic processing in close proximity to the ER were comprehensively reviewed in detail 87 167. Briefly, the NHB1 signal peptide of Nrf1 enables its TM1 to be integrally anchored within the ER membranes in a co-translocational way 203 303, and also determines its topological folding of adjacent domains (and amphipathic helix-adjoining domains) to be selectively partitioned into either the luminal side (e.g., its transactivation domains) or the cytoplasmic side (e.g., its DNA-binding CNC-bZIP domains) of membranes 275 304 (**Figure 10C5**). In the ER lumen, Nrf1 is allowed for N-linked glycosylation in its NST domain by oligosaccharyl transferase to yield an inactive glycoprotein 203 305 306. When required for biological cues, dynamic repositioning of the luminal-resident transactivation domain (TAD) of this intact CNC-bZIP factor is driven by p97-fueled retrotranslocation machinery through the Hrd1-leading retrotranslocon across membranes to enter the cytoplasmic side 275. Once its NST domain of Nrf1 is dislocated out of the ER, this protein is allowed for deglycosylation by N-glycanase 1 (NGLY1), so that its amino acid sequence is re-edited from those glycosylated asparagines to deglycosylated aspartates, thus potentiating its transactivation activity 275 307; (similar work was later confirmed on Skn-1 308). In the cytoplasmic subcellular compartments, Nrf1 is also likely modified by O-GlcNAcylation in its Neh2L region of TADs 309 and its Neh6L adjacent to CNC-bZIP domain, respectively 310 311. Such similar but differential modifications can bi-directionally (i.e., negatively and positively) monitor the protein stability of Nrf1 and its transactivation activity. In the extra-ER subcellular compartments, this CNC-bZIP protein is further subjected to selective proteolytic processing by cytosolic proteases DDI1/2 or proteasomes to yield an active N-terminally-truncated CNC-bZIP factor (e.g., Nrf1/TCF11^ΔN^), together with distinct lengths of other short isoforms (e.g., Nrf1β and Nrf1γ) 122 312 313 314. Such distinctive functional isoforms are released from membranes and translocated into the nucleus, in which each isoform of Nrf1 is only enabled to form a functional heterodimer with its partner sMaf or other bZIP proteins, before ensuring them to exert different transcriptional regulation of target genes 87.

Collectively different tempo-spatial modifications and selective proteolytic processing of Nrf1 are dominantly dictated by the dynamic topovectorial folding and trafficking of this CNC-bZIP protein within and around the ER to dislocate the nucleus, which determines its protein stability and transcription activity to regulate cognate genes 87 315. This notion is further supported by experimental evidence provided by Martinon’s group 316. They also found that both Rad23A and Rad23B are required for proteolytic processing of Nrf1 by DDI2, which was promoted by HRD1-mediated ubiquitination of this CNC-bZIP protein 316. However, our experimental evidence revealed that the ubiquitination of Nrf1 is not a prerequisite for its proteolytic processing, but it is further activated by de-ubiquitination by USP19, enabling Nrf1 to rescue from the ER-associated protein degradation (ERAD) by proteasomes 317. Like Rad23 and Dsk2, DDI2 can also serve as a shuttling factor, but it contains a retroviral protease domain that influences the binding of ubiquitylated proteins (e.g., Nrf1) through its N-terminal ubiquitin-like domain (UBL) and ensuing proteasomal degradation 318. Altogether, these indicate that DDI2 determines the selective proteolytic processing of Nrf1 to either yield a mature active factor (by this protease) or to be targeted for proteasomal degradation (via this shuttling factor). However, Nrf1 is, indeed, also likely processed by other DDI1/2-independent proteolytic pathways 319. More importantly, rapid recovery of proteasome activity from sub-lethal proteasome inhibitors is DDI2-independent, which occurs before transcription of proteasomal genes is upregulated by Nrf1 but requires protein translation 320. The N-terminal domain of Nrf1 (containing NHB1 and NHB2) was further predicated to fold as an atypical UBL 122, similar to the equivalents of DDI1, DDI2, Rad23 and Dsk2 (Figure S7). Overall, the selective proteolytic processing of Nrf1 by DDI1/2 and/or proteasomes depends on the dynamic tempo-spatial (re)positioning of NHB2 adjoining with its putative UBL domain in different topovectorially-localized subcellular compartments 122 312 321, even though the unique topogenetic folding of Nrf1 is dictated by its NHB1-adjioning TM1 orientation within and around the ER.

It should be important to note that in unstressed conditions, only a fraction of Nrf1 is allowed for proteolytic degradation mediated by ubiquitin proteasome system, because most of this CNC-bZIP protein is *in situ* protected by ER membranes and then ferried through such ER-associated endomembrane network to reach the inner nuclear membrane (INM) 305, whilst its functional domains are repositioned in the nucleoplasmic compartments, so that it can be enabled to exert its unique physio-biological functions. In such topogenetic dislocation of Nrf1 from the ER to the nucleus, it is likely allowed for its topovectorial processing by another DDI1/2-independent pathway to yield a mature CNC-bZIP factor, which activates its cognate genes responsible for maintaining normal physiological homeostasis and organ integrity.

In addition, a specific variant Nrf1D arises from alternative splicing of mRNA transcripts to give rise to a reading frameshift mutation, leading to a constitutive substitution of the intact Nrf1’s C-terminal 72-aa residues (covering the second half of its zipper motif to the C-terminal Neh3L domain) by another extended 80-aa stretch, which was folded into a redox-sensitive transmembrane domain (TMc, **Figure 10A**), enabling it to be tightly integrated within the ER membranes, beyond its NHB1-adjoining TM1 region 322. Furthermore, Nrf1D was hitherto identified to act as the first candidate secretory transcription factor, albeit its precursor was predicated to fold as an integral transmembrane-bound CNC-bZIP protein that entails dynamic topologies within and across ER membranes 322. However, it is unknown about how to be proteolytically processed by a not-yet-identified protease within the TMc-adjoining domain, before being unleashed from the ER and then secreted to enter the blood.

## UNIQUE PATHOPHYSIOLOGICAL PHENOTYPES OF NRF1 THAT ARE DISTINCTIVE FROM NRF2

**Figure 11:**
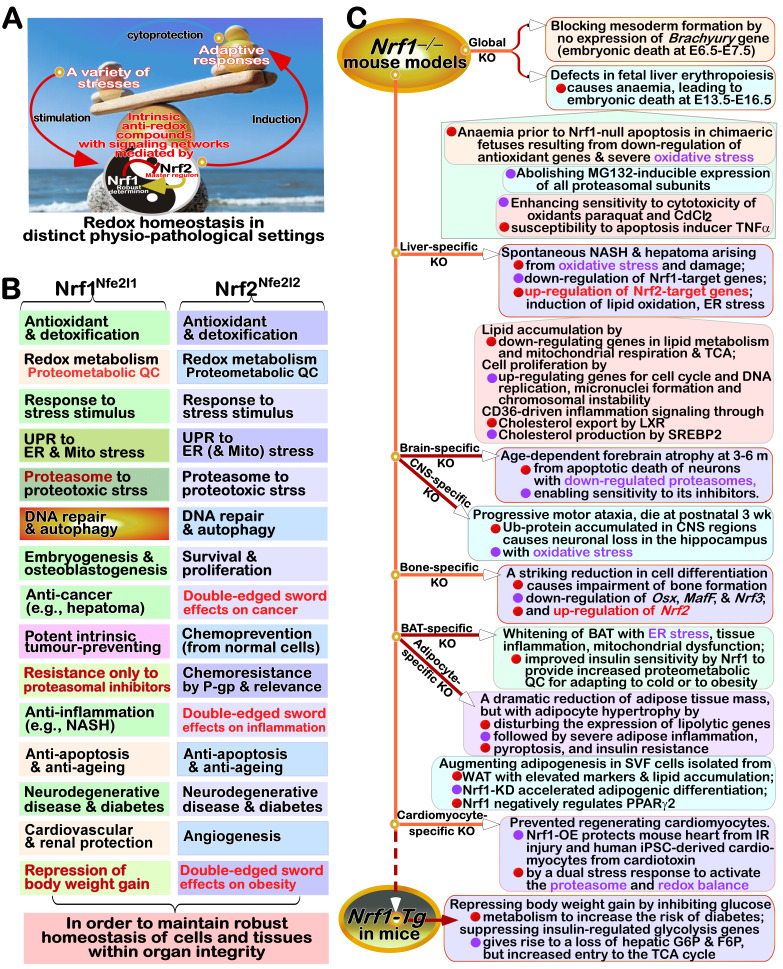
FIGURE 11: Unique and significant pathophysiological functions of Nrf1 that are distinguishable from Nrf2. **(A) **Schematic diagram of inter-regulation between Nrf1 and Nrf2, to exert uniquely differentiated yet integrated roles in governing intrinsic anti-redox compounds involved within multi-hierarchical signaling networks so as to mediate adaptive cytoprotective responses against a variety of cellular stress. **(B)** Functional distinctions between Nrf1 and Nrf2 evinced in at least 15 different aspects. Nrf1 is manifested as its unique physio-pathological functions that are distinctive or event absent from Nrf2. By sharp contrast, Nrf2 acts as a versatile chameleon-like master regulon and thus often plays certain ‘double-edged sword’ effects on some aspects. **(C)** Schematic representation of different tissue-specific loss of Nrf1’s function in animal models, which are all significantly manifested with distinct pathophysiological phenotypes. Rather, constitutive activation of Nrf1 (to gain its function in *Nrf1-Tg:MGRD* mice) can also give rise to observing phonotypes, which were induced by a high-fat diet.

From the molecular phylogenetic evolution of the CNC-bZIP family as described above (**Figure 8**), its members are diverged up on vertebrates 114. Of note, Nrf1 and Nrf2 are two major principal CNC-bZIP members in mammalians, of which Nrf1 appears to act de facto as an orthologue possibly by gene vertical transfer from its ancestors, whilst Nrf2, together with other homologues, should be viewed as a redundant paralogue (i.e., para-orthologues). Such ratiocination is convincingly evidenced by supportive gene-targeting experiments, revealing an objective fact that global *Nrf2^-/-^* knockout (KO) mice are viable and fertile, with neither any obvious defects nor typical pathological phenotypes (e.g., spontaneous cancer) occurring during embryonic development and postnatal growth 118 323. This demonstrates that Nrf2 is not necessary for normal development and healthy growth 324, even though *Nrf2^-/-^* mice were observed to be more susceptible than wild-type mice to chemical carcinogens 325. By contrast, Nrf1 is indispensable for determining cell homeostasis and organ integrity, because it is innately endowed with unique remarkable features that are distinctive from Nrf2 87 167 (**Figure 11A** and **B**). This is just based on those facts that were discovered by gene-targeting strategies for KO of *Nrf1* that are employed to create distinct animal models with significant pathological phenotypes (**Figure 11C**) 261 262 326 327 328 329.

### Global KO of *Nrf1* results in embryonic lethality

Apart from elevated expression in the heart, midbrain and head mesenchyme between embryonic day (E) 8 and 9, the single *Nrf1/Nfe2l1* gene is ubiquitously expressed with a constant level of its mRNA transcripts detected in all other tissues from E7.5 to E17.5 330. Conversely, global KO of *Nrf1^-/-^* in mice leads to embryonic lethality at E6.5 to E14.5, resulting from severe oxidative stress damages 326 327 328. This presages that the loss of Nrf1 cannot be compensated by Nrf2, even though both factors can elicit similar overlapping functions in regulating ARE-driven gene expression 331 as confirmed by their double KO (*Nrf1^-/-^:Nrf2^-/-^*) mouse model 332. This fact demonstrates that Nrf1, rather than Nrf2, fulfills its unique and indispensable functions for embryonic development.

#### Genetic deletion of Nrf1’s aa 172-741 causes embryonic lethality by blocking mesoderm formation 

The first global KO mutant of *Nrf1* (i.e.,* Lcrf1^tm1uab^*, directly deleting 3.5-kb of its gene sequence with a loss of its aa 172-741) resulted in the embryonic lethality in early gastrulation on Black Swiss outbred backgrounds 327. The *Nrf1^-/-^* embryos developed to the late egg cylinder stage indistinguishable from wild-type counterparts, but thereafter arrested at E6.5 and most died before E7.5. This is attributable to the failure to form the primitive streak mesoderm with no *Brachyury (T)* gene expression, even though ectoderm and visceral endoderm layers appeared normal. However, the homozygous *Nrf1^-/-^* defect was rescued after injection into wild-type blastocysts, and such mutant embryonic stem cells (ESCs) remained to contribute to all cell lineages in examined chimeras 327. Overall, this *Nrf1^-/-^* defect, despite being not cell-autonomous, demonstrates that the unique function of Nrf1 is essentially exerted for the transcriptional expression of a subset of critical genes that are constitutively involved in controlling the mesoderm formation (principally by epithelial mesenchymal transition (EMT) 333 334 335).

#### Targeted disruption of Nrf1’s bZIP domain causes embryonic death of anemia

The second global knock-in mutant of *Nrf1* (i.e., *Nrf1^rPGK-neo^*, by reversely inserting phosphoglycerate kinase-neomycin cassette was into 5‘-end of Nrf1’s bZIP-coding region for targeted disruption of *Nrf1*) resulted in embryonic lethality at mid-late gestation from E13.5 to E18.5 on C57BL/6J blastocyst background 328. The *Nrf1^-/-^* embryos died *in utero* of decreased definitive enucleated red cells and the ensuing anemia, which results from impaired maturation of erythroid progenitors in the microenvironments of the foetal liver, but without increased apoptosis of the haematopoietic cells 328. Despite such a lack of cell autonomy, Nrf1 retains to serve as an essential gene for red cell maturation because none of the compensatory functions were efficiently executed by haematopoiesis-specific NF-E2 p45, Nrf2 and/or other CNC-bZIP factors that are expressed at high levels 336 337 338. Furtherly, the hepatocyte-specific function of Nrf1 in foetal and adult livers is not rescued by the putative compensative function of *Nrf2* 172. This suggests that there may exist certain overlapping functions of two CNC/bZIP factors and their potential redundancy in early embryogenesis and development. This is supportively evidenced by double KO of *Nrf1^-/-^:Nrf2^-/-^* leading to a relatively earlier embryonic death at E10.5, which was attributable to extensively increased apoptosis and severe oxidative stress-induced growth retardation 332.

Then, mechanistic studies revealed that the p53-Noxa-mediated apoptosis was markedly induced by elevated ROS (by measuring hydrogen peroxide and singlet oxygen), which results from severely impaired expression of antioxidant defense genes (e.g. *Mt-1, Gclm, Gclc, Ferritin H, Ho-1, Nqo1*, but not *Sod1/2*), when compared to the individual *Nrf1^-/-^* or *Nrf2^-/-^* 332. This implies that there exists at least a part of the overlapping functions of Nrf1 and Nrf2 in regulating those genes essential for the intracellular redox homeostasis during early embryogenesis, which is determined possibly by both their co-expression patterns 337 338 339 and their amino acid sequence similarity beyond the CNC/bZIP domains 87. In addition, MG132-induced transcriptional expression of the proteasome (PSM) genes was also abrogated in the mouse embryonic fibroblasts (MEFs) of *Nrf1^-/-^* , but not of *Nrf2^-/-^* 340. Lastly, a disparity in such obvious pathophysiological phenotypes between *Nrf1^rPGK-neo^* 328 and *Lcrf1^tm1uab^* 327 is much likely to be attributable to different strategic gene manipulations, because the former *Nrf1^rPGK-neo^* mice also remained to be allowed for varying expression of certain Nrf1 isoforms (e.g., a C-terminally-truncated negative mutant) 326 328 340 341 342.

#### Genetic ablation of Nrf1’s bZIP domain leads to the hepatocyte apoptosis in the late gestation

Additionally, a lethal phenotype of the homozygous *Nrf1^-/-^* embryos was obtained by deleting its 2.2-kb genomic fragment encoding the bZIP domain to yield the *Nrf1^lacZ^* mice 172), which was similar to that of *Nrf1^rPGK-neo^*; their livers were smaller, lighter and hypoplastic, prominently by E13.5-E14.5 328. However, such developmental arrest cannot be interpreted by the impaired erythropoiesis in the foetal liver, because these erythropoietic cells still grew normally *in vitro* and the *Nrf1^-/-^* ESCs were also efficiently contributed to erythroid cells of chimeric mice generated with the wild-type blastocysts 328. Several chimeric embryos were also observed with anemia, which occurred before the detection of cell death in the liver parenchyma 172. From this, it is inferable that the anemia is secondary to the failure to sustain haematopoiesis in the impaired liver microenvironment.

To circumvent the embryonic lethality, alternative chimeric mice were generated by injecting positive *Nrf1^-/-^* ESC clones with 129/Sv background into the C57BL/6J mouse blastocysts where Nrf1 is normally expressed 172. By characterizing these chimeric mice at 8 to 16 weeks of age, it was found that loss of *Nrf1* resulted in an impaired contribution of such *Nrf1^-/-^* mutant ESCs to the adult, rather than foetal, hepatocytes, even though they were still contributable to other tissues (e.g., lung, kidney, muscle and heart) in the adult animals, wherein Nrf1 was highly expressed, if under normal genetic conditions 172. Significantly, although the *Nrf1^-/-^* ESCs were contributable to the foetal liver development at E14.5, the hepatocytes in the chimeric embryos underwent widespread apoptosis in late gestation 172. Further examinations uncovered that the hepatocyte apoptosis resulted from increased oxidative stress and decreased expression of antioxidant genes (e.g., *Gclc, Gclm, Gpx1, Ho-1*, and *Mt-1/2*). Therefore, these demonstrate a cell-autonomous role for Nrf1 in protecting the hepatocytes from apoptosis and also enabling their healthy survival during late foetal development, primarily by maintaining normal redox homeostasis.

### Liver-specific KO of Nrf1 causes spontaneous development of NASH and hepatoma 

Since there existed massive cell death and degeneration in the livers of chimeric embryos derived from the *Nrf1^-/-^* ESCs 172, such chimeric animal models cannot be employed to determine the critical role of Nrf1 in the maturation of hepatocytes. To bypass this obstacle, conditional KO models of *Nrf1* in the mouse livers were created by using a specific Cre-loxP transgenic system, aiming to determine the unique essential function of Nrf1 in adult hepatocytes beyond embryonic development 261 262.

#### Adult hepatocytes-specific deletion of Nrf1’s aa 296-741 results in NASH and hepatoma

Liver-specific KO of *Nrf1* (to delete the fourth exon encoding aa 296-741, i.e., LCR-F1/Nrf1β) in the adult mouse resulted in a typically pathologic phenotype that resembles NASH and hepatic neoplasia, including hepatocellular adenomas and carcinomas, which spontaneously developed as early as four months after birth 261. Before cancer development, the *Nrf1^-/-^* livers also exhibited interrelated steatosis, apoptosis, necrosis, inflammation and fibrosis. In addition to such precancerous lesions, loss of *Nrf1^-/-^* per se may also be directly contributable to tumourigenesis by promoting chromosome mis-segregation 343. The tamoxifen-inducible KO of *Nrf1^-/-^* (from *Nrf1^flox/flox^:Cre-ERT2*) increased the numbers of abnormal nuclei and micronuclei by 3-fold higher than controls. Such *Nrf1^-/-^*-led genetic instability appears to be closely associated with decreased expression of the kinetochore genes *Ndc80, Nuf2*, and *Spc25*, as bad as the spindle assembly checkpoint gene *Sgol1* 343. Together, these suggest a possibility that Nrf1 could function as a tumor repressor in the hepatocytes. 

To gain an insight into the molecular pathological basis for NASH caused by *Nrf1^-/-^* in livers, further experiments revealed that the Nrf1-deficient hepatocytes acquired for increased susceptibility to oxidative stress and relevant damages, along with down-regulation of some ARE-battery genes (e.g. *Gstm3, Gstm6* and *Gstp2*) but up-regulation of *Cyp4A* genes 261. The loss of Nrf1’s function resulted in a significant increase in the intracellular ROS levels, which were at least in part generated by β-oxidation of fatty acid using proliferated microsomal CYP4A (i.e., in the ER). The increased ROS levels are also associated with elevated lipid and other pathogenesis leading to NASH. Similar pathological damages were also observed in the mouse liver of another hepatocyte-specific *Nrf1^-/-^* model 262. Such pathophysiological phenotypes demonstrate that Nrf1 acts as a unique vital player in mediating the expression of critical genes for maintaining intracellular redox and lipid homeostasis in livers. Further experimental evidence revealed that lipid accumulation in *Nrf1^-/-^* livers at five weeks resulted from the up-regulation of 1,500 genes and down-regulation of 1,700 genes 344. Such genes involved in lipid metabolism (e.g., *Lipin1, PGC-1*β, *PPAR*α), amino acid (e.g., methionine) metabolism, the TCA cycle and mitochondrial respiration, as well as 26S proteasomes, were decreased, whereas other genes responsible for the cell cycle and DNA replication were increased, but these Nrf1-target genes are unaffected by loss of Nrf2 or Keap1.

A bulk of ubiquitinated and/or oxidative damaged proteins, besides lipids, was also accumulated in the *Nrf1^-/-^* hepatocytes, leading to the ER stress-associated steatosis 345. This is just attributed to impaired transcription of the PSM genes and increased expression of ER stress response genes (e.g., *Atf4, Atf6, Bip, Chop, Gadd45*β, *Herp*); this was accompanied by phosphorylation of the PERK-eIF2α signaling 345. Further microarray analysis revealed down-regulation of 52 Nrf1-dependent genes (i.e. *Mt1/2, Clrf, Gcn20, Gadd45*γ,* Mfsd3, Pdk4* and *Spp3*) by >= 3-fold. Intriguingly, the adaptive activation of 20 Nrf2-target genes by the single KO of *Nrf1^-/-^* was abolished by another double KO of *Nrf1:Nrf2* 262. This demonstrates that Nrf1 exerts an essential physiological function required for the basal constitutive expression of a subset of cytoprotective responsive genes against endogenous (redox, proteotoxic and lipotoxic) stresses (that trigger the activation of Nrf2). Rather, no significant changes in those prototypic Nrf2-target genes (e.g., *Gclc, Gclm, Gss, Nqo1*) were observed upon acute hepatic loss of Nrf1 in the 3-methylcholanthrene- inducible KO mice (*Nrf1^flox/flox^:Cyp1A1-Cre*), which also led to profound NASH, but without obvious oxidative stress 346. Collectively, these demonstrate that Nrf1 enables to regulate a separate battery of genes, that are distinctive from those regulated by Nrf2.

#### Cholesterol-led NASH is deteriorated by the hepatocytes-specific loss of Nrf1’s bZIP domain

The hepatocyte-specific KO of Nrf1 (to delete a fragment spanning exons 4 and 5 of this gene encoding its DNA-binding domain in the homozygous mice obtained by crossbreeding C57BL/6N-A/a chimeric males with C57BL/6J females) led to the substantial aggravation of cholesterol-led pathological phenotype in the liver that resembles human hepatosteatosis and NASH 347. Such cholesterol-fed *Nrf1^-/-^* mice developed dramatically weighted fatty livers, showing massive lipid accumulation and indications of hepatocyte ballooning and damage, although no increases in their body weights, as compared to wild-type control mice 347. The cholesterol-exposed *Nrf1^-/-^* mice exhibited a substantial rise in the cholesterol esters accounting for more than 50% of total lipid in the livers. Such an elevated presence of cholesterol, cholesterol ester, and cardiolipin, whilst all other classes were unchanged, coincided with a greater ratio of cholesterol to phospholipid in livers. These indicated a critical physiological role of Nrf1 for the hepatocytes in protecting the livers from excessive cholesterol. This notion is further evidenced by subsequent experiments, revealing that Nrf1 controls a transcriptional program for hepatocyte adaptation to cholesterol as a means of averting stress and limiting cholesterol accumulation, although repression of such Nrf1 activity is indeed evoked by cholesterol 347. This repression of Nrf1 by cholesterol is further aggravated by directly sequestrating this CNC-bZIP factor within ER membranes through its cholesterol recognition amino acid consensus (CRAC) motifs 122 275 304 347, such that their connecting (PEST-)degrons can be buried in the lumen and hence escape from its proteolytic processing, to form a negative regulatory feedback circuit on the Nrf1-target genes.

**Figure 12:**
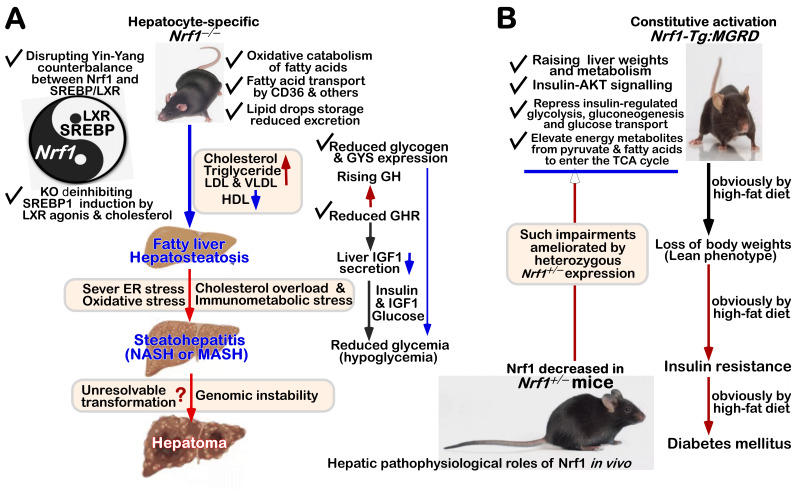
FIGURE 12: Comparison of liver-specific *Nrf1^-/-^* phenotypes with those of the constitutive active *Nrf1-Tg:MGRD* mice. **(A) **Schematic representation of the collective pathophysiological phenotypes of liver-specific *Nrf1^-/-^* mice, which are manifested with spontaneous development of progressive fatty liver diseases, such as hepatosteatosis, nonalcoholic steatohepatitis (NASH) or metabolism-associated steatohepatitis (MASH) and even hepatoma, as also accompanied by reduced glycemia (hypoglycemia) in some cases. Of note, a major pathogenic mechanism accounting for these phenotypes results from disrupting the ‘Yin-Yang’ counterbalance between Nrf1 and SREBP2/LXR 347. **(B)** The constitutive activation of *Nrf1-Tg:MGRD* mice to gain its function also results in a lean phenotype with insulin resistance and even development of diabetes mellitus, which are more obviously induced by high-fat diet 329, but ameliorated by heterozygous *Nrf1^-/-^* mice.

Conversely, Nrf1-deficient livers develop severe pathological problems, because they were relatively refractory to the transcriptional changes induced by a cholesterol challenge, i.e., only within 93 differentially expressed genes (DEGs, 0.91%) out of 10,174 detectable genes, whereas 826 DEGs (8.12%) were measured in cholesterol-fed Nrf1 wild-type mice 347. Further, 790 DEGs identified in the wild-type cases were absent from *Nrf1^-/-^* livers, which were defined as Nrf1-dependent genes regulated by cholesterol, and enriched by gene ontology clustering to the acute inflammatory response, lipid transport and sterol metabolic process 347. Further experimental analysis of *Nrf1^-/-^* livers with cholesterol-led increased levels of H_2_O_2_ and phosphorylated JNKs, revealed that Nrf1 suppresses cholesterol accumulation and stress-triggering inflammation (NASH) by down-regulated *CD36* (and *AbcA1, AbcG1, Gltp, ApoC2, F4/80, C1Q8, Orm2, Saa2*), but also promotes cholesterol excretion by up-regulated *Cyp7a1, Cyp7b1* and *Cyp8b1* (besides *Ces1f* and *Insig1*), when compared to wild-type controls. Notably, induction of *Srebp1c, AbcA1* and* AbcG1* by cholesterol or the liver X receptor (LXR) agonist GW3965 in *Nrf1^-/-^* primary hepatocytes was blunted by ΔNT-Nrf1, which acts as a constitutively active but cholesterol insensitive isoform of Nrf1, but could not rescue Nrf1-deficient livers. This implies that this much likely relates to an interference of the nuclearly-located Nrf1 factor on LXR (and/or SREBP1), which is consistent with the role of LXR as a multifaceted countermeasure against excess cholesterol. Besides, two ER membrane-spanning transcription factors Nrf1 and SREBP2 (**Figure 10**) are also likely co-evolved as a ‘Yin-Yang’ counterbalance whereby SREBP2 promotes cholesterol production, while Nrf1 promotes cholesterol removal (**Figure 12**), to achieve cholesterol homeostasis and stabilize relevant metabolic activity.

#### The pathogenesis of NASH is accelerated by combined deficiency of Nrf1 and Nrf2 in hepatocytes

The reduced growth of *Nrf1*, but not *Nrf2*-deficient mice (with 80% knocked out by floxed Nrf1/Nrf2-encoding alleles at 14 days after infecting a liver-targeting adeno-associated virus expressing Cre recombinase via thyroxine binding globulin promoter) was observed, albeit both groups exhibited normal appearance and behavior 348. By contrast, substantial weight loss and morbidity (e.g., poor grooming and slow movement) were caused by both deficiencies of *Nrf1:Nrf2*; such effects were so severe that 46% (5/11) of males and 27% (3/11) of females did not survive for 28 days 348. This is attributable to increased lipids (i.e., triglyceride and cholesterol) deposited in the livers of combined *Nrf1:Nrf2* deficient mice, along with sex-dependent increases in the plasma cholesterol, low-density lipoprotein (LDL) and very low-density lipoprotein (VLDL) cholesterols in the males but not females, apart from reduced secretion of liver VLDL in *Nrf1*- or *Nrf1:Nrf2*-deficient mice. 

The histopathological phenotype of modestly increased steatosis and inflammation (i.e., steatohepatitis) was manifested in the *Nrf1*, but not *Nrf2*-deficient livers, and their combined deficiency caused a significant phenotype of steatohepatitis, resulting in the hepatocyte ballooning 348. This was accompanied by increased mRNA levels of *chemokine ligand 2* (*ccl2*), TNFα, TGFβ, and *collagens* (*col1a1, col1a2, col3a1*), and also increased plasma alanine transaminase (ALT) in *Nrf1:Nrf2*, or *Nrf1*, but not *Nrf2* deficient livers. The steatohepatitis caused by *Nrf1*-deficiency was also featured by p62-enriched Mallory-Denk bodies, intracellular hyaline inclusion bodies or aggresomes, aside from lipid droplets. Cholesterol crystals, as a hallmark of steatohepatitis and hepatic cholesterol overload 349 350, were determined in *Nrf1* but not *Nrf2*-deficient livers, and significantly increased by combined deficiency of both factors in both males and females 348. Collectively, these demonstrate certain complementary roles of Nrf1 and Nrf2 in guarding against hepatic cholesterol overload, increased triglyceride storage, cholesterol crystallization, and diet-induced steatohepatitis, and the roles are also vital for whole-body viability. Conversely, the combined deficiency of hepatocyte *Nrf1:Nrf2* accelerated the pathogenesis of steatohepatitis, as accompanied by increased cholesterol accumulation and crystallization, altered bile acid metabolism, and decreased biliary cholesterol. 

Such severe steatohepatitis in nutrient-overloaded livers was much more likely ameliorated by combined induction of *Nrf1* and *Nrf2*, as therapeutic co-targets for effectively treating the metabolic associated steatohepatitis (MASH). This notion is further supported by the experimental evidence revealing that the therapeutic effects of the Nrf2-activating drug bardoxolone methyl require Nrf1, as a key contributor, due to their co-regulation of immunometabolic stress defense genes that eliminate cholesterol overload and mitigate oxidative stress damage and inflammation 348. The complementary gene programming of Nrf1 and Nrf2 can thus counteract a pathological progressive continuum of cholesterol-associated fatty liver diseases (including NASH). As such, the modest pathological phenotype caused by deficiency of *Nrf1*, rather than *Nrf2*, cannot be rescued by the latter compensatory increased expression of Nrf2 by ~40% of its mRNA levels in the *Nrf1*-deficient livers 348. This strongly demonstrates that Nrf1 should have to exert an essential role, that is irreplaceable by Nrf2, in the hepatic pathophysiological process (**Figure 12**).

Further RNA-sequencing analysis revealed that those functions related to the synthesis, transport and metabolism of cholesterol and bile acids were inhibited by altered retinoid X receptor (RXR)-dependent pathway activity 348. The RXR-linked control of immunometabolism was disrupted by hepatocytic deficiency of Nrf1 and Nrf2. Their co-target gene-binding activity was also confirmed by ChIP to *txn1* (thioredoxin 1, for redox stress defense), *lonp2* (lon peptidase 2, for peroxisomal proteostasis defense), and *abcg8* (ATP binding cassette subfamily G member 8, for cholesterol excretion); all three were reduced by combined *Nrf1:Nrf2* deficiency to a greater degree than that by single deficiency of *Nrf1* or *Nrf2*. By sharp contrast, Nrf1-specific targets *Psma1* and *Psmc2* were, to similar extents, reduced by deficiency of *Nrf1* or *Nrf1:Nrf2*, whilst Nrf2-target *Ces1g* (carboxylesterase 1G) and *Gstm1* (glutathione S transferase mu 1) in biotransformation and phase 2 detoxification were, to similar extents, reduced by deficiency of *Nrf2* or *Nrf1:Nrf2*. Together, these overlapping and exclusive genome sites enable to which Nrf1 and Nrf2 interact responsible for cognate target gene regulation in the liver.

Combined *Nrf1:Nrf2* deficiency also resulted in greater reduction of *PPAR*α (peroxisome proliferator activator receptor α) and *CAR* (constitutive androstane receptor), two known transcription factors that regulate lipid, bile acid and peroxisome metabolism, as well as liver detoxification and ROS defense, along with reduced *BAAT* (bile acid-coenzyme-A amino acid N-acyltransferase), *AbcG5, PRDX1* (peroxiredoxin 1) and *Cat* (catalase), as compared to single deficient cases 348. Conversely, greater increases in the two known NASH-drivers *IHH*(Indian hedgehog) and *tnfrsf12a* (TNF receptor superfamily member 12A) were caused by combined *Nrf1:Nrf2* deficiency, and also accompanied by increased expression of 4-hydroxynonenal (4HNE)-conjugated proteins, as well as of TAZ (taffazin) and YAP1 (yes-associated protein 1), which drive IHH expression and steatohepatitis 351 352. Collectively, these demonstrate a co-regulatory role for Nrf1 and Nrf2 in hepatobiliary detoxification of cholesterol and bile acids.

Recently, it was further found that significantly reduced levels of HDL (high-density lipoproteins) cholesterol and apolipoprotein A1 (ApoA1) were caused by combined deletion of *Nrf1:Nrf2*, but not by their single deletions 353. This was also accompanied by a decreased capacity of HDL to accept cholesterol undergoing efflux from macrophages and to counteract the TNFα-induced inflammatory effect on endothelial cells. This coincided with substantial alterations in the HDL-resident proteome, which fully correlated with liver gene expression profiles of corresponding proteins (e.g., *ApoA1, ApoA2, ApoA4, ApoE, C3. C9, ApoJ, Cpn2, Gpld1, Itih2*, and *Itih4*); they are responsible for mediating reverse cholesterol transport, reducing inflammation, and abrogating oxidative damage. Their modest changes were also observed from the deficiency of *Nrf1* but not of *Nrf2* 353. Thereby, these revealed a coordinating control of HDL functioning by Nrf1 and Nrf2 to improve hepatoprotective and even atheroprotective actions, in which Nrf1 likely exerts a vital role. 

#### Hepatocyte-specific deficiency of Nrf1 results in reduced glycemia (hypoglycemia)

Albeit as stress-defense transcription factors, both Nrf1 and Nrf2 can also exert their complementary but exclusive regulatory roles in programming genes responsible for hepatocytic glucose metabolism and even systemic glucose homeostasis 354 355 356. Conversely, reduced glycemia (hypoglycemia) was caused by hepatocyte-specific loss of *Nrf1* or *Nrf1:Nrf2*, but not *Nrf2*, in mice (fed with a mild stressful fat-fructose-cholesterol diet for one to three weeks), but this phenotype did not occur in the leptin-deficient mouse model with obesity and diabetes (*leptin^ob/ob^:Nrf1^flox/flox^*) 356. This indicates there exists an Nrf1-supporting defense for hepatocytes to counteract hypoglycemia but not promote hyperglycemia or diabetes. As consistent fully with this, Nrf1 deficiency resulted in reduced levels of hepatic glycogen and glycogen synthase (e.g., *GYS2*) expression, as also accompanied by decreased insulin and insulin-like growth factor-1 (IGF1), and increased growth hormone (GH), with no marked alterations in all other examined glycemia-influencing hormone (e.g., glucagon, ghrelin, cortisol, triiodothyronine, thyroxine, and gastric inhibitory polypeptide) 356. Although the capacity of *Nrf1*-deficient livers to produce glucose via gluconeogenesis appeared to be unaffected 356, glucose starvation-induced rapid death of human *Nrf1*α, but not *Nrf2*-deficient hepatoma cells results from its fatal defects in the redox metabolism reprogramming 354. Altogether, these reveal an essential role of Nrf1 in modulating glucose homeostasis setting for the steady-state euglycemia through the GH-IGF1 signaling axis to the liver glycogen storage (**Figure 12**). In turn, reduced expression of GH receptor (by sensing GH to stimulate liver IGF1 secretion) was contributable to reduced glycemia caused by deficiency of *Nrf1* or *Nrf1:Nrf2*, but not *Nrf2* 356.

### Gain-function of *Nrf1* loses body weights but gains the risk of diabetes with insulin resistance

The physio-pathological function of Nrf1 seems somewhat uninterpretable by the evidence obtained from the above-mentioned loss-of-function mutants of Nrf1 in mice, because they manifested with severe liver dysfunction and metabolism disorders. To bypass this, the gain-of-functional transgenic mice (i.e., *Nrf1-Tg:MGRD*) were thereby created by engineering over-expression of the Nrf1^3xFlag^, which is driven by the MafG-regulated promoter domain 329. Consequently, increased expression of *Nrf1* leads to a loss of body weight in *Nrf1-Tg:MGRD* mice fed with normal and even high-fat diets. Thereafter, the high-fat diet-induced obesity model mice further developed a pathology resembling human diabetes mellitus, also with markedly higher levels of blood glucose after insulin injection 329. Therein, the pancreatic islets of *Nrf1-Tg:MGRD* mice were much larger than those of wild-type mice, as the insulin-positive areas were increased, implying constitutive activation of insulin secretion in *Nrf1-Tg:MGRD* mice leading to systemic insulin resistance. Similar insulin resistance also occurred in the normal-fed *Nrf1-Tg:MGRD* mice, with a positive correlation of increased Nrf1 expression.

The induction of insulin resistance appears to be interpreted by disruption of cell signaling to metabolic perturbation by forced Nrf1 expression; this was evidenced by the supportive findings 329 that: i) insulin-induced phosphorylation of Akt-Ser473 was obviously prevented in the liver and skeletal muscle, but not in white adipose tissues, of *Nrf1-Tg:MGRD* mice, implying that Nrf1 suppresses insulin signaling to Akt phosphorylation; ii) *Nrf1-Tg:MGRD* mice showed increased weights of livers, but not skeletal muscle or white adipose tissues; such increased weights of livers were further augmented by the high-fat diet, with altered hepatic metabolism; iii) both glucose utilization and production in livers were impaired by *Nrf1-Tg:MGRD*’s repressing those insulin-regulated glycolysis-related genes (e.g., *GCK, AldoB, PGK1* and *PK*), gluconeogenesis-related genes (e.g., *Fbp1* and *Pck1*) and glucose transporter 2 (*Glut2*), particularly in the high-fat diet conditions; iv) the entry of glucose into the glycolytic pathway was suppressed by *Nrf1-Tg:MGRD*, leading to marked decreases in the levels of glucose-6-phosphate (G6P) and fructose-6-phosphate (F6P), as accompanied by increased pyruvate, along with reduced lactate, such that the entry of the TCA cycle was increased, with elevated levels of acetyl coenzyme A (CoA), citrate and ATP in the liver of *Nrf1-Tg:MGRD* mice fed by high-fat diets; v) the utilization of hepatic CoA to generate β-hydroxybutyrate was increased from other fuels (i.e., fatty acids) beyond glucose and amino acid catabolism. Lastly, such impaired glucose metabolism was also obviously ameliorated by decreased expression of Nrf1 in heterozygous *Nrf1^+/-^* mice, when compared with wild-type mice, even though both were fed high-fat diets. Collectively, impaired insulin signaling and dysregulated glucose metabolism by constitutive induction of *Nrf1-Tg:MGRD* results in the development of diabetes mellitus triggered by the high fat diet (**Figure 12**). However, except that insulin-induced Akt phosphorylation was repressed by forced expression of Nrf1, no obvious changes in the expression levels of major components of the insulin signaling pathway were determined by microarray analysis of the liver and skeletal muscle of *Nrf1-Tg:MGRD* mice 329. This indicates that insulin resistance is triggered by Nrf1 through a distinct pathway from transcriptional repression of the insulin signaling components.

In addition, a single nucleotide polymorphism (SNP) rs3764400 located in the 5‘-flanking region of the human *Nrf1* gene represents a risk factor for obesity with body mass index (BMI, with a *P* value of 5 x 10^-6^) 357. The reporter gene activity showed that rs3764400, as a regulatory SNP of *Nrf1* expression with a ~2.2-fold higher level, was driven by the risk C allele, when compared to that mediated by its control *T* allele, within the 2.1-kb promoter region upstream from the first transcription start site 329. The major T allele of rs3764400 (5‘-CAGT/CT-3‘ 357) composes a consensus c-Myb-binding sequence 5‘-CAGTT-3‘ (reversed by 5‘-AACNG-3‘) 358, whilst the minor C allele (enabling for induction of *Nrf1* 329) cannot allow c-Myb related factors to bind the altered motif. Thus, it is inferable that c-Myb is much likely to act as an upstream regulator of Nrf1 for transcriptionally controlling glucose metabolism, but its dysfunction leads to increased expression of *Nrf1* contributable to the pathophysiology of obesity and relevant glucose metabolic dysregulation. Moreover, differential regulation of glucose metabolism by Nrf1 and Nrf2 is supported by further experimental evidence revealing that blood glucose levels were increased by gain-of-function of *Nrf1* 329, but decreased by loss-of-function of *Nrf1* but not of *Nrf2* 356, as bad as decreased by gain-of-function of *Nrf2* 359 360.

### Pancreatic loss of Nrf1 causes impaired insulin secretion and glucose metabolism leading to diabetes

The failure of pancreatic β-cells to secrete insulin sufficiently to meet the increasing demand of glucose metabolism is defined as a major contributor to type-2 diabetes with the secondary loss of β-cells. The impaired β-cell function to reduce glucose-stimulated insulin secretion (GSIS) is a critical pathophysiological event of diabetes. Coincidently, β-cell-specific Nrf1-KO mice exhibit severe fasting hyperinsulinemia, as accompanied by reduced GSIS and glucose intolerance 361. The pathophysiological phenotype results from deficiency of almost all Nrf1 isoforms in mouse islets and MIN6 β-cells, leading to a marked increase in basal insulin release with reduced GSIS; this was viewed as a β-cell phenotype reminiscent of the early stage of type-2 diabetes. To our knowledge, hyperinsulinemia has been accepted as a compensatory response to insulin resistance and its prolonged status can also deteriorate induction of insulin resistance and even obesity 362 363. Thereby, the pathological phenotype of pancreatic Nrf1-KO mice is likely to account for ensuing glucose intolerance. 

Further biochemical examinations of *Nrf1*-deficient islets and β-cells revealed substantially increased ratios of both NADPH/NADP and ATP/ADP, which is attributable to enhanced glycolysis and mitochondrial metabolism (e.g., β-oxidation) rather than oxidative phosphorylation 361. The silencing of Nrf1 up-regulated expression of glucose transporter 2 (*Glut2*) and glycolytic proteins, such as lactate dehydrogenase 1 (*Ldh1*), glyceraldehydes 3-phophate dehydrogenase (*Gapdh*), hexokinase 1 (*HK1*), but with down-regulation of glucokinase (*Gck*). Interestingly, such impaired glycolysis and GSIS resulting from the loss of Nrf1’s function can also be rescued by the knockdown of *HK1*. However, no changes in the expression profiling of mitochondrial protein complexes I, II, cytochrome c and ATP synthase F1, as well as mitochondrial biogenesis-related PGC-1α, PGC-1β and nuclear respiratory factor 1 (called aPal^NRF1^ 364) were determined in Nrf1-deficient cells, when compared with wild-type controls 361. Collectively, β-cell-specific depletion of *Nrf1* leads to a shift from the oxidative phosphorylation to aerobic glycolysis for energy, a consequence similar to the Warburg effect in cancer cells 365 366. Furthermore, altered expression levels of p53, phosphorylated AKT and AMPKα were observed in Nrf1-deficient cells 265 355 or in the constitutive active mice of *Nrf1-Tg:MGRD* 329. These imply these signaling molecules are involved in altered glucose metabolism. Altogether with the evidence provided by 367, it is inferable there may exist a couple of metabolic reprogramming switches with signaling responses to endogenous redox stress status in Nrf1-defienct cells.

### Adipose-specific loss of *Nrf1* disrupts its plasticity, adaptive thermogenesis and metabolic homeostasis

Adipocytes possess remarkable adaptive capacity to respond to nutrient excess, fasting or cold exposure, with distinct types of adipocytes for the maintenance of proper metabolic health. Brown adipose tissue (BAT) is a crucial metabolic organ in facultative thermogenesis (acute response), also with great plasticity to respond to long-term cold adaptive thermogenesis. The thermogenic capacity of BAT is dependent on the neurohumoral response to sympathetic stress stimulation 368. Such BAT is distinguishable from white adipose tissue (WAT), just because it is richly innervated by the sympathetic nervous system (SNS, in which norepinephrine is released at the nerve endings activating adrenergic receptors on brown adipocytes) and highly vascularized in brown adipocytes, with several small lipid vacuoles and many large mitochondria 369. In addition to such critical roles in maintaining thermal homeostasis and even energy homeostasis, BAT is involved in plasma triglyceride clearance and glucose homeostasis 370 371. This fact demonstrates an essential functionality of BAT to combat obesity and metabolic diseases (e.g., diabetes and cardiovascular disease). By contrast, WAT is composed of a population of specialized white adipocytes for energy storage (in the form of triacylglycerols) and its mobilization (as fatty acids), and hence, long considered as an inactive tissue that primarily served as a thermal insulation purpose. Lipid and glucose metabolism in white adipocytes also confers a pivotal role to WAT in whole-body homeostasis, particularly energy homeostasis 372. Conversely, dysfunction in white adipocyte metabolism is also viewed as a cardinal event in the development of insulin resistance and associated disorders (e.g., type 2 diabetes mellitus). In such distinct adipose tissues, the specific knockout of Nrf1, as a key thermogenic factor in mice, resulted in different pathophysiological phenotypes 374 375.

#### BAT-specific KO of Nrf1 in mice that cannot adapt to rising thermogenic activity

Conditional deletion of Nrf1 expression was created by using homozygous floxed *Nfe2l1* alleles (coding its DNA-binding domain) mice under the control of the *Ucp1* promoter-driven Cre (to yield *Nfe2l1^ΔBAT^* mice) or *Ind.Nfe2l1^-/-^* mice raised by tamoxifen-inducible *Cre* under another control of the chicken β-actin promoter-enhancer coupled with the cytomegalovirus immediate-early enhancer (*CAGGCre-ER^TM^*) 373 and then subjected to investigating a linkage of cold adaptive response to its target proteasome in BAT. This is due to ERAD to remove those unfolded, damaged or dispensable proteins by the ubiquitin-proteasome system (UPS) in this UPR^ER^, and it was also found, from adipocyte-specific deficiency of IRE1α (encoded by *Ern1*) or XBP1 in mice, that this conserved canonical branch of the UPR^ER^ is dispensable for the BAT-mediated homeostasis 373.

As compared to that at 30°C, cold adaptation caused markedly increased expression of Nrf1 in wild-type BAT, (aside from the minor expression in inguinal WAT). The induction of Nrf1 by exposure to cold in brown adipocytes yielded higher levels of the ER-localized form of Nrf1 protein, as well as its cleaved, transcriptionally active form in BAT. By contrast to wild-type controls, markedly lower expression of Nrf1 at mRNA and protein levels was observed in cold-adapted *Nfe2l1^ΔBAT^* mice, implying thermoneutrality is a state of natural *Nrf1* deficiency 373. Thermogenesis requires cold-inducible proteasomal functionality driven transcriptionally by Nrf1, just because cold adaptation of BAT induces Nrf1 to increase proteasomal activity crucially for maintaining ER homeostasis and cellular integrity, particularly while the cells were placed in a state of high thermogenic activity. Under such thermogenic conditions, BAT-specific deletion of *Nrf1* enabled *Nfe2l1^ΔBAT^* mice in a hyper-ubiquitinated state and hence resulted in ER stress, metainflammation, markedly diminished mitochondrial function and whitening of the BAT. This suggests that Nrf1 is required for the BAT activity to maintain metabolic adaptation, besides ER homeostasis, which was supported by *Nfe2l1^ΔBAT^*-leading hyper-ubiquitinated 228 proteins that were most notably enriched with mitochondria and organelle homeostasis, particularly related to ER proteins and energy metabolism. Thus, Nrf1 in thermogenic fat cells acts as a metabolic guardian, preventing tissue stress and inflammation, independently of BAT differentiation, mass or expandability. 

Mice lacking Nrf1 in brown adipocytes are born normal, but alterations in the BAT of *Nfe2l1^ΔBAT^* develop as a function of cold and age. At thermoneutrality, where BAT activity is minimal, Nrf1 was lowered in BAT, but in this environment neither proteasome inhibition nor genetic deficiency of Nrf1 is more deleterious for the tissue. This indicates that Nrf1-mediated transcriptional programming is required for adaptation to rising thermogenic activity. This is also corroborated by the phenotype of tamoxifen-induced deletion of Nrf1 expression in adult *Ind.Nfe2l1^-/-^* mice 373. The BAT thermogenic activation following cold adaptation or treatment with CL316243, an adipocyte-specific β3-adrenergic agonist 376 at thermoneutrality de facto requires an Nrf1-mediated increased activity of proteasomes to fulfill its metabolic potential, but such proteasomal activity is impaired in obesity. In mouse models of both genetic and dietary obesity, stimulation of proteasomal activity by adenovirus-mediated expression of Nrf1 or the proteasome activator PA28a to enhance proteostasis in BAT resulted in obviously improved insulin sensitivity. Altogether, Nrf1 emerges as a novel guardian of brown adipocyte function, providing enhanced proteometabolic quality control for thermogenic adaptation to cold or to obesity.

Another recent study revealed that adipocyte-specific Nrf1-KO [*Nfe2l1(f)*-KO] mice develop an age-dependent whitening and shrinking of BAT, with signatures of the down-regulated proteasome, impaired mitochondrial function, reduced thermogenesis, pro-inflammation and elevated regulatory cell death 377. This is attributable to the fact that deficiency of Nrf1 in brown adipocytes predominantly caused down-regulated expression of lipolytic genes (*PNPLA2, ATGL, HSL, MGL, PLIN1*), decelerating lipolysis so that BAT cannot fuel thermogenesis and thus leading to hypertrophy of brown adipocytes (enriched with larger lipid droplets and swollen mitochondria), inflammation related to regulatory cell death, and consequently cold intolerance. Further single-nucleus RNA-sequencing of BAT unraveled that deficiency of Nrf1 caused significant transcriptomic reprogramming, and led to aberrant expression of a variety of genes involved in thermogenesis (*Ucp1*), lipid metabolism (e.g., *Prdm16, Elovl3, Cpt1b, Ppar*α), mitochondrial (respiratory) stress (*Cox8b, Cox7a*1, PGC1α COXIV, NDUFS4, FIS1,OPA1), inflammatory response (*Adgre1, Ifng, IL1b, Cd68, F4/80*), and regulatory cell death (*Casp1, Nlrp3, Pycard*), besides proteasome, in distinct subpopulations of brown adipocytes 377. Overall, these demonstrate that Nrf1 functions as a key transcription factor determining the thermogenesis, subsequently cell fate and heterogeneity of BAT in mice by controlling their lipidometabolic and proteometabolic homeostasis.

#### Adipocyte-specific KO of Nrf1 disrupts WAT plasticity and metabolic homeostasis

*Nfe2l1(f)*-KO mice were generated by crossing mice bearing a floxed Nfe2l1 allele 262 with mice expressing another adipocyte-specific Cre recombinase under the control of adiponectin gene promoter (*Adipoq-Cre*) 374. Then, it was found that the *Nfe2l1(f)*-KO mice exhibited abnormal fat distribution, with dramatically reduced mass of subcutaneous adipose tissues, but slightly increased mass of gonadal WAT. Importantly, Nrf1-deficient mice also displayed glucose intolerance, insulin resistance, adipocyte hypertrophy, and severe adipose inflammation 374. Furtherly, mechanistic studies revealed that deficiency of Nrf1 caused significantly diminished expression levels of a set of genes involved in adipogenesis, lipogenesis, lipolysis and general adipocyte function in inguinal WAT, but was accompanied by dramatically increased expression of macrophage markers and inflammatory response genes, such as *Adgre1* (adhesion G protein-coupled receptor E1), *Cd68, Ifng* (interferon γ), *IL1b* (interleukin 1β), as well as pyroptosis-related genes, e.g., *Casp1* (caspase1), *Nlrp3* (NLR family pyrin domain containing 3), and *Pycard* (apoptosis-associated speck-like protein containing a CARD 374. Notably, the expression of adipogenic genes (*Pparg2* and *Cebpb*) was significantly increased in the gonadal WAT of *Nfe2l1(f)*-KO mice, but no marked changes in the mitochondrion-related *Ppargc1a* and *Cox8b* were observed when compared to controls. Collectively, these demonstrate that the deficiency of Nrf1 results in aberrant expression of genes related to lipolysis in WAT, leading to adipocyte hypertrophy followed by inflammation, pyroptosis, and even insulin resistance. Therefore, this finding revealed a vital role for Nrf1 in regulating adipose tissue plasticity and energy homeostasis.

An in-depth examination of the stromal vascular fraction isolated from the WAT of *Nfe2l1(f)*-KO mice revealed augmented adipogenesis, along with elevated expression of adipogenic markers and lipid accumulation 378. The enhanced and accelerated adipogenic differentiation was further corroborated by stable knockdown of Nrf1 in 3T3-L1 pre-adipocytes. Conversely, such adipogenesis induced by dexamethasone-methylisobutylxanthine-insulin (DMI) was substantially attenuated by forced overexpression of Nrf1α, but not any of its shorter isoforms, in 3T3-L1 cells 378. Mechanistic investigation revealed that Nrf1α negatively regulates the transcription of peroxisome proliferator-activated receptor γ (*PPAR*γ), particularly PPARγ2 (as a master regulator of adipogenesis), as well as *Cebp*α,* Cebp*δ,* aP2, CD36* and *Glut4*. Altogether, these indicate that Nrf1α is much likely to act as a critical negative regulator of adipogenesis by suppressing PPARγ, whilst Nrf1β may play a contrary role in basal expression of PPARγ.

The crucial roles of Nrf1 constitutively in adipocytes were further evaluated by treatment of juvenile *Nfe2l1(f)*-KO mice with CL316243, a β3 adrenergic agonist to promote the effect of lipolysis on adipose inflammation 379. The 4-week-old juvenile adipocyte-specific Nrf1-KO mice displayed a normal fat distribution, but exhibited reduced fasting plasma glycerol levels and elevated adipocyte hypertrophy and macrophage infiltration in inguinal and gonadal WAT, when compared to adult mice. This is likely to result from decreased expression of multiple lipolytic genes and reduced lipolytic activity in WAT of *Nfe2l1(f)*-KO mice 379. Their altered expression of inflammation- and pyroptosis-related genes, as well as macrophage infiltration in the WAT, was dramatically alleviated following CL316243 treatment for seven days. However, the phenotype of *Nfe2l1(f)*-KO mice was aggravated after treatment for three weeks with rosiglitazone (a thiazolidinedione agonist of PPARγ, applied for the treatment of type 2 diabetes mellitus by stimulating genes in favor of storage of triglycerides), as accompanied by increased expression of those genes related to inflammation and pyroptosis in their shrunk WAT 380. Collectively, these highlight a notion that, since Nrf1 plays a fundamental regulator of the lipolytic gene expression and defends relevant metainflammation, it may be, thereby, considered as a potential therapeutic target to improve adipose plasticity and lipid homeostasis, as well as whole-body energy homeostasis.

### Skeletal myocyte-specific KO of Nrf1 results in an oxidative lean phenotype with muscle remodeling

Skeletal myocyte-specific *Nrf1-mKO* mice were generated by crossing the floxed-Nfe2l1 mice 373 with mice expressing *Acta1-Cre* (skeletal muscle-specific Cre recombinase under the control of the actin-α1 promoter) 381. The mKO mice displayed lowered body weights, which were predominantly caused by slightly lower lean mass, but not fat mass. A prominent reduction of the gastrocnemius muscle in size and weight was also associated with lower grip strength in *Nrf1-mKO* mice compared to wild-type controls 381. A major phenotypic change in the gastrocnemius muscle of *Nrf1-mKO* mice unraveled a shift towards a more oxidative profile. Such phenotypic switch towards more oxidative fibers was also supported by analyzing myosin heavy chain gene expression, as indicated by higher levels of slow-twitch *Myh7* and lower levels of fast-twitch *Myh2*. Of note, the perinatal myosin heavy chains *Mhy3* and *Mhy8* were markedly higher in *Nrf1-mKO* mice, implying the presence of regenerative muscle fibers. An insight into the muscle ultrastructure by transmission electron microscopy revealed that the loss of Nrf1 leads to the aberrant myofibrillar organization in muscle tissues of *Nrf1-mKO* mice, where its mitochondria seemed bulged and disrupted, with less coenzyme Q:cytochrome c reductase, a component of the oxidative phosphorylation Complex-III 381. Less respiration after addition of the ADP and succinate, as bad as reduced maximal respiration capacity, was detected in isolated muscle fibers of *Nrf1-mKO* mice, when compared to wild-type controls. Collectively, these demonstrate a fiber-type switch from fast to slow-twitch fibers, with aberrant mitochondrial bioenergetics in the gastrocnemius muscle of *Nrf1-mKO* mice.

Knockdown of Nrf1 by silencing siRNA led to lower levels of proteasomal activity and subunit gene expression (*Psma1, Psmb1*) in differentiated C2C12 cells, but with higher levels of ubiquitylated proteins after proteasome inhibition by bortezomib 381. Similarly, genetic deletion of myocytic Nrf1 resulted in markedly lower proteasome subunit gene expression in *Nrf1-mKO* muscle tissues, so that the ex vivo proteasomal activity was blunted, leading to markedly higher levels of ubiquitylation 381. Rather, trypsin-like activity remained higher in tissue from *Nrf1-mKO* mice, possibly indicating a dynamic remodeling of proteasomal activity independently of Nrf1. Overall, these demonstrate the importance of myocyte Nrf1 for proteasomal function and UPS in skeletal muscles. This is further evidenced by proteomic hyperubiquitylation (of 2832 differentially expressed) in *Nrf1-mKO* muscles, which highlighted energy metabolism and filament organization as major pathways 381. RNA-seq analysis of soleus and gastrocnemius muscles from matched *Nrf1-mKO* mice uncovered the imperative role of Nrf1 in energy metabolism and the UPS, and also pointed towards its impact on respiration, ATP metabolic process, and muscle cell differentiation 381. Metabolomics of the gastrocnemius muscle revealed a strong difference in metabolites related to glucose metabolism, the Warburg effect and anti-oxidative characteristics. Therefore, the critical role of Nrf1 for UPS and proteostasis in skeletal muscle dictates muscle function and energy metabolism, but the loss of Nrf1 causes a profound phenotypic change in the gastrocnemius muscle, whilst the impact on soleus was rather mild.

By a linear regression modeling of the relationship between energy expenditure and body weight, it was found that energy expenditure was higher in the *Nrf1-mKO* mice, which displayed more food intake to fuel their elevated energy expenditure, but no evident differences in physical activity, as compared to wild-type controls 381. This indicates that systemic energy metabolism for animal whole body is regulated by myocyte Nrf1 *in vivo*. Conversely, loss of myocytic Nrf1’s function protects the mice from body-weight gain and metabolic imbalance caused by high-fat diets, because mice lacking Nrf1 displayed hormetic energy metabolism and resistance to high fat diet-induced obesity, and were associated with a lean phenotype and muscle fiber type switching 381. Distinct metabolic conditions of obesity were analyzed by a multi-omics approach to be associated with recalibration of the UPS in muscle. Overall, these define an adaptive role for the fine-tuning proteasome towards UPS governed by Nrf1 in the remolding of muscle proteome and its biological function.

### Cardiomyocyte-specific KO of *Nrf1* prevents neonatal heart regeneration and repair 

The cardiomyocyte-specific *Nrf1-cKO* mice (generated by crossbreeding *Nrf1^fl/fl^* mice with α*MHC-Cre* transgenic mice) exhibited no aberrations in cardiac morphology or function at 2-month age 382. But, following myocardial infarction (MI), *Nrf1-cKO* hearts showed decreased cardiac function (measured as fractional shortening, ejection fraction, and myocardial wall motion), but with increased fibrotic scarring. This is due to a greater myocardial loss at 1-day post MI, and markedly decreased proliferation of cardiomyocytes at 3-day post MI in the *Nrf1-cKO* hearts underlying the impaired heart regeneration, as accompanied by notably reduced proteasomal activity 382, albeit with an enhanced inducible subpopulation of cardiomyocytes that respond to injury by apoptosis and hypertrophy. During heart regeneration, the oxidative phosphorylation (OXPHOS) pathway was downregulated in wild-type cells, but upregulated (to inhibit cardiomyocyte proliferation) in *Nrf1-cKO* hearts, leading to a significant increase of ROS, along with upregulated expression of two cardiac stress markers *Nppa* and *Nppb* in the *Nrf1-cKO* MI hearts 382. By contrast, downregulated genes in *Nrf1-cKO* hearts were enriched with actin filament-based processes, the PI3K-AKT and Wnt signaling pathways, aside from the known Nrf1-target genes (*Hmox1* and *Psmd1*). Such a loss of Nrf1 prevented neonatal cardiomyocytes from initiating the transcriptional response required for heart regeneration.

After ischemia/reperfusion injury, the mice pre-injected with the Nrf1-expressing adeno-associated viral vector (AAV9-Nrf1) showed significantly reduced infarct area (from 52% to 40%) and also improved their cardiac function. Additional increases in diastolic and systolic volumes of the left ventricle were observed in control MI hearts, but profoundly attenuated in AAV9-Nrf1 treated mice, which show reduced cardiac dilation and remodeling, as well as reduced fibrotic scarring 382. These indicate that overexpression of Nrf1 *in vivo* confers a cardoprotective effect in adult hearts following ischemic injury. This is also supported by further experimental evidence obtained from the neonatal rat ventricular myocytes (NRVMs) overexpressing Nrf1, revealing a consistent protective effect of this factor against all examined cardiotoxins (e.g., H_2_O_2_, peroxynitrite, doxorubicin, elastin). Notably, such protective effect of Nrf1 was much greater than that of Nrf2 in response to all these cardiotoxin treatments 382, though the latter Nrf2 was also viewed as a master regulator to confer cardioprotection 383. This fact highlights an important role of Nrf1 as a key regulator of cellular stress in the heart. Fully consistent with this, significantly increased proteasome-mediated proteolyses, such as proteasome protein catabolism and ERAD pathways, but specifically down-regulated ECM organization, were determined in NRVMs overexpressing Nrf1, but not Nrf2, but both shared with antioxidant and anti-inflammatory responses. To clarify potential redundant functions between Nrf1 and Nrf2, cardiomyocyte-specific *Nrf2-cKO* or *Nrf1:Nrf2*-dKO mice were generated 382. Consequently, the expression levels of proteasome (*Psma1, Psmd1*, and *Psmb3*) and antioxidant target genes (*Hmox1, Sod1*, and *Cat*) were reduced to greater degrees in *Nrf1-cKO*, when compared to *Nrf2-cKO* hearts, but importantly not further reduced in the *Nrf1:Nrf2*-dKO hearts, implying that Nrf1 plays a predominant role in regulating the expression of these genes in the heart.

By analysis of human heart ventricles from 431 donors (age 20–79) in the Genotype Tissue Expression (GTEx) database, varying expression levels (differing by over 70-fold) of Nrf1 were revealed among individuals, with a significant decline in the elderly demographic (age 60–79) 382. In these samples, 1167 genes were identified by co-expression analysis as being highly correlated with Nrf1 (Spearman correlation coefficient > 0.8, adjusted p-val < 0.00001). Apart from known Nrf1-targets *PSMC1, PSMD7, SOD1* and *CAT*, Nrf1 co-expressed genes are clustered with key metabolic and energy production processes in the hearts (e.g., fatty acid degradation, electron transport chain, mitochondrial organization, and muscle contraction) and known Nrf1 downstream pathways (ubiquitination, proteasome degradation, and autophagy). From these, it is inferable that the degree of Nrf1 expression correlates with the metabolic and contractile properties of the heart and, hence likely affects cardiac function. Conversely, a significant reduction of Nrf1 transcript levels in heart ventricles from three independent patient cohorts with heart failure (n = 28) was revealed by further analysis of published datasets 382. Doxorubicin-induced cardiomyopathy in cancer patients carries a poor prognosis and also is frequently fatal. However, overexpressing Nrf1 enables human induced pluripotent stem cell-derived cardiomyocytes (hPSC-CMs) to be sufficiently protected from doxorubicin-induced cardiotoxicity (and other cardiotoxins). Altogether, these unravel that the adaptive responsive mechanism mediated by Nrf1’s enabling proteasome activation and redox homeostatic balance is required for neonatal heart regeneration and also confers cardioprotection in the adult heart. Therefore, it is plausible that re-activating this mechanism in the adult heart could represent a potential therapeutic approach for cardiac repair.

### Bone-specific deficiency of *Nrf1* impairs osteoclast differentiation and bone formation

A mechanistic insight into osteoblast differentiation induced by the redox inducer vitamin C (i.e., ascorbic acid) 384 385 revealed a significant inducible increase in the expression of osterix (Osx), as a key osteoblast-specific transcription factor that was principally regulated by Nrf1-binding to its ARE sequence, in impaired bone marrow stromal cells 386. The mutant cells were originally derived from the spontaneous fracture mice caused by lacking the *gulonolactone oxidase* gene and hence leading to a deficiency of vitamin C 387. Albeit all three Nrf members have a potential to bind the ARE sequence in the *Osx* promoter, its expression induced by Nrf1 was 50- and 500-folds higher than those done by Nrf2 and Nrf3, respectively; the latter two CNC-bZIP factors were also unaffected in vitamin C-treated bone marrow stromal cells 386. From these, it is thereby inferable that Nrf1 is essential for transcriptional regulation of the *Osx*-mediated gene expression profiling, which is further supported by knockdown of *Nrf1*, showing decreased *Osx* expression, impaired osteoblast differentiation and mineralized nodules formation from bone marrow stromal cells 386. By sharp contrast, Nrf2 was considered to negatively regulate the differentiation of osteoblasts and chondrocytes 388 389.

The *in vivo* physiological function of Nrf1 in regulating bone formation was further determined by using osteoblast-specific KO mice generated by crossbreeding *Nrf1^flox/flox^* mice 390 with transgenic mice bearing *Col1*α*2-iCre* (in which Cre recombinase is under the control of the regulatory sequence of *collagen-1*α*2 (Col1*α*2*)) 391. The osteoblast-specific Nrf1-KO mice showed obvious reductions in bone size, peak bone mass, trabecular number and mechanical strength, also as accompanied by decreased expression of *Osx*, reduced cell differentiation, and impaired bone formation. Intriguingly, deficiency of *Nrf1* enabled *Nrf2* to be up-regulated by 40% but also allowed *Nrf3* and *MafF* to be down-regulated by 60% and 50%, respectively 391, implying there exists a coordinated control mechanism amongst them. However, it should be noticed that a possible complementary pathway may be controlled by Nrf1 and Nrf3, because their transcript levels were obviously decreased upon exposure of both wild-type and *Nrf3^-/-^* mice to butylated hydroxytoluene 392, and their glycoproteins were co-localized and integrated within and around the ER in a similar membrane-topological folding fashion 275 305 393 394. 

KO of all isoforms of *Nrf1*’s transcripts specifically in themyeloid lineage in *Nfe2l1(M)*-KO mice (yielded by crossing mice bearing the floxed *Nfe2l1* allele with mice expressing *LysM-Cre*) caused the increased activity of osteoclasts, decreased bone mass and worsening of osteoporosis induced by ovariectomy and aging 395. However, no significant effects of Nrf1’s loss on the osteoblast and osteocytes were observed in *Nfe2l1(M)*-KO mice. Further investigations of the bone marrow cells and RAW 264.7 macrophages revealed that deficiency of *Nrf1* leads to accelerated and elevated osteoclast differentiation, which is attributable to enhanced accumulation of ROS in the early stage of osteoclast differentiation and expression of *Nfatc1*α (encoding nuclear factor of activated T cells, cytoplasmic, calcineurin-dependent 1α) 395. Such Nrf1-regulated osteoclast differentiation and the transcription expression of multiple antioxidant genes, as well as *Nfatc1*α were isoform-specific. The full-length Nrf1α likely functions as accelerators of osteoclast differentiation with induction of *Nfatc1*α and antioxidant genes, whilst Nrf1β serves as a brake controlling the accelerator effect of Nrf1α in check. This indicates differential and even opposing roles of multiple distinct Nrf1 isoforms in the osteoclast genesis and differentiation, bone remodeling and metabolism homeostasis, to provide an understanding of the pathogenesis of various bone disorders including osteoporosis and arthritis.

It was, in undifferentiated odontoblasts, found that the physical interaction of Nrf1 with C/EBPβ through their bZIP domains, concerning the binding efficiency depending on its phosphorylation by PKA at Ser^569^ (i.e. Ser^599^ situated on the boundary between the Neh6L and CNC domains in human TCF11) directs the specificity of the CNC-bZIP function to repress the basal constitutive expression of the *DSPP* (*dentin sialophosphoprotein*) gene encoding two specific markers *DPP* (dentin phosphophoryn) and *DSP* (dentin sialoprotein) 396. Conversely, the loss of such interaction between Nrf1 and C/EBPβ, in fully differentiated odontoblasts, caused an increase in the transcriptional expression of *DSPP* to yield DPP and DSP. These demonstrate a coordinated control of odontoblast differentiation by both bZIP factors to switch *DSPP* on or off.

### Brain-specific KO of *Nrf1* leads to the pathogenesis of neurodegenerative diseases

Nrf1 expression is also found in neuronal systems, and its evident function in central nervous system has been proven in a few studies 390 397 398. Only two groups have been working on the neuronal tissue-specific KO of Nrf1 *in vivo*. A neuronal-specific Nrf1 KO was achieved using Nestin as a marker for neural stem cells in the Cre-lox system 397 398. The resulting *Nrf1*-deficient mice displayed a notable phenotype, showing growth retardation starting at seven days postnatal and mortality by three weeks. Behavioral abnormalities, such as motor ataxia and feeding difficulties, were evident, along with hindlimb clasping reflex, indicative of neurodegenerative conditions. Interestingly, other similar phenotypes are also observed in small Maf-deficient mice (i.e., *MafG^-/-^*:*MafK^-/-^*) 399 400, suggesting the importance of cooperation between Nrf1 and small Maf proteins in the neuronal functions. Histological analysis highlighted neuronal degeneration in the hippocampus CA3 region and spinal cord. Notably, Nrf1-deficient neurons exhibited severe oxidative stress in the spinal cord, with ubiquitinated protein accumulation in the cortex's pyramidal neurons, other brain regions, and the spinal cord. Nestin expression is seen in beyond neural stem cells 401. Therefore, more specific drivers of Cre are requested to ensure the role of Nrf1 in the neuronal system. Nevertheless, Nrf1 KO by using the CaMK2cre system has demonstrated that loss of Nrf1 in the brain resulted in an accumulation of ubiquitinated proteins and reduced expression of proteasomal subunits, including *PsmB6* 390. Atrophy in the forebrain and decreased cortex and hippocampus thickness were observed, with significant neuronal loss and bind-limb clasping reflexes at three to four months, suggesting an essential involvement of Nrf1 deficiency in neurodegenerative pathogenesis, highlighting its causal role in neurodegeneration.

The loss of Nrf1 in the brain, as demonstrated by the CaMK2Cre-mediated deletion, has unveiled significant implications: neurodegeneration and impaired proteasome function due to the down-regulation of various proteasomal genes, whereas the NestinCre-mediated Nrf1 deletion exhibits similar phenotypes without reduced expression of proteasome genes. Although the NestinCre-mediated Nrf1 deletion exhibits ubiquitinated protein accumulation, only Nrf1’s loss via CaMK2Cre-mediated gene deletion showed reduced proteasome gene expression, possibly due to differences in the mouse models. The NestinCre model entails the global neuronal Nrf1 knockout, whereas CaMK2Cre specifically targets the cortex and hippocampus. Additionally, while CaMK2Cre demonstrates neural damage via apoptosis without oxidative stress, no evidence of neural cell apoptosis was also observed in the NestinCre model. More importantly, the NestinCre-mediated knockout of *Nrf1* unveiled a loss of ubiquitin-specific proteases (USPs), with USP9x identified as one of the Nrf1 target genes 398. Because USPs play crucial roles in regulating neural activities, for instance, the presence of a mutation in USP14 in an ataxia mouse model leads to severe tremors and premature death 402. Additionally, the reduction of Usp9x promotes the degradation of Survival Motor Neuron protein, thereby inducing improvement in Spinal Muscular Atrophy 403. USP9x expression is notably high in specific brain regions, including layer V of the neocortex, certain hippocampal subfields, and Purkinje cells in the cerebellum of adult mice 404. Higher expression is found in the CA3 region than the CA1 region in hippocampal pyramidal cells, suggesting co-localization with Nrf1. Notably, the knockdown of *USP9x* induces the formation of toxic α-synuclein inclusions USP9X in SH-SY5Y cells upon proteolytic inhibition 405, indicating its role for neuroprotection, but its dysfunction may trigger Parkinson’s disease. Therefore, the reduction in USP9x expression resulting from Nrf1 loss may lead to the accumulation of polyubiquitinated proteins, with implications for neurodegenerative pathogenesis processes. Yet, further in-depth molecular assessments are warranted to identify the specific proteins accumulating in a ubiquitinated state upon the loss of Nrf1’s function in the neuronal system. 

In addition, it should be also noted that no single KO of all other CNC-bZIP factors (i.e., *Nfe2p45, Nrf2, Nrf3, Bach1* and *Bach2*) rather than *Nrf1* gave rise to any apparent neuronal phenotypes 323 406 407 408 409. From this, it is inferable that Nrf1 is just the only candidate within the CNC-bZIP family responsible for neuronal homeostasis 398. Nrf1 does contribute to the development of the central nervous system after birth, because this was evidenced by conditional KO of *Nrf1* in brains 390 397, although global KO of the gene did not cause any apparent neuronal deficits in mouse embryonic stages before death 172 326.

## UNIQUELY DIFFERENTIATED YET INTEGRATED ROLES OF NRF1 AND NRF2 IN GOVERNING CELL HOMEOSTASIS DURING DISTINCT LIFE PROCESSES

From the eco-evo-devo view, Nrf1 is a highly conserved fossil-like indispensable CNC-bZIP transcription factor with ancient properties, whilst the relative younger Nrf2 is inferable to arise from the ancient gene differentiation and hence emerges in the later evolved vertebrates. As two major principal CNC-bZIP factors in mammalians, Nrf1 and Nrf2 often seem to resemble two entangled ‘Yin-Yang’ quanta, which comply with a dialectic law of the unity of opposites in biosciences (**Figures 3** and **11A**). Such allelopathic potentials of Nrf1 and Nrf2 are sufficiently evoked for their coordinated control of adaptive responsive protective mechanisms to maintain cell homeostasis and organ integrity within certain presetting physiological thresholds. This notion is evidenced by those supportive experiments by comparison of the combined *Nrf1:Nrf2*-deficient versus their respective single deficient mice 332 348. Therefore, it is of crucial importance to gain insights into the uniquely differentiated, yet integrated, roles of Nrf1 and Nrf2 in governing robust physiological homeostasis and healthy survival during distinct life processes.

### Distinct regulation of Nrf1 and Nrf2 in redox responsive signaling against cancer development

**Figure 13:**
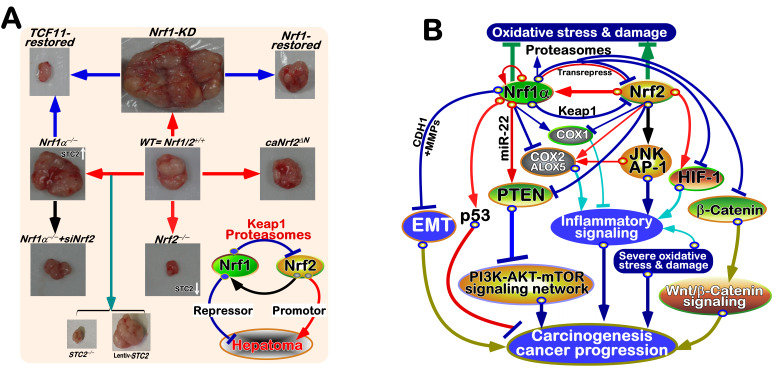
FIGURE 13: Inter-regulation of between Nrf1 and Nrf2 in the opposing control of cancer progression. **(A) **Different phenotypes of xenograft tumor-bearing model mice, which were inoculated with each of those indicated mutant or restored cell lines, as compared with their progenitor wild-type (WT, Nrf1/2^+/+^) human hepatoma HepG2 cells. These tumors were obtained from those relevant references 123,265,413,414. Of sharp note, Nrf1 acts as a potent tumor-repressor, whereas Nrf2 serves rather as a potential tumor-promotor, although both CNC-bZIP factors are inter-regulated with each other. **(B)** The tumor-promoting effect of Nrf2 is tightly confined by the robust expression of Nrf1 through its negative regulator Keap1 and Nrf1-target proteasome-mediated degradation pathway, albeit Nrf1 is transcriptionally controlled by Nrf2. Such inter-regulatory roles between Nrf1 and Nrf2 in oppositely and also coordinately governing multiple signaling networks accounting for the strict control of cancer development and progression, in addition to mediating antioxidant, detoxification and cytoprection against oxidative stress and concomitant damages.

The above-described distinct tissue-specific *Nrf1^-/-^* mice are manifested with certain typical pathologies, each of which resembles human non-alcoholic steatohepatitis and hepatoma 261,262, type-2 diabetes mellitus 361 and neurodegenerative diseases 390 397. These facts demonstrate that mouse Nrf1 (and/or its derived isoforms) fulfills an indispensable function in regulating critical target genes responsible for maintaining robust physiological development and growth under normal homeostatic conditions. By contrast, *Nrf2^-/-^* mice all are viable and fertile, without typical pathological phenotypes 118 323, implying it is not essentially required for normal development and growth. As such, Nrf2 has still been generally accepted as a master regulator of *ARE*-battery gene expression 116 410. This is based on the observation that *Nrf2^-/-^* mice were more susceptible than wild-type mice to chemical carcinogens 325, and also induction of Nrf2 (to activate *ARE*-driven genes) has been recognized as a potential chemopreventive and therapeutic target against carcinogenesis and its malignant progression 116 175 411. However, the hyperactive Nrf2 activity is also contrarily reconsidered as a potent oncogenic driver with those relevant hallmarks of cancer, because of its tumor-promoting effects exerted *bona fide* on carcinogenesis, cancer progression, metastasis, and even drug resistance to therapy 265 412. Such ‘double-edged sword’ effects of Nrf2 on cancer prevention and progression have led us to take into account seriously how the opposing activity of this factor should be tightly confined by Nrf1 (as deciphered in **Figure 13**).

#### Distinct regulation of Nrf1 and Nrf2 with Keap1-proteasomal and p62-autophagic disposing systems 

A major role of Nrf2, as a master redox regulon of an antioxidant transcription factor that is activated in adaptive hormesis response to oxidative stress, facilitates the maintenance of cellular redox homeostasis, particularly upon being threatened by the oxygenated environments 4 64 111 176 415 416. In the absence of redox stress, the transcriptional activity of Nrf2 is rigorously confined by its negative regulator Keap1, as a redox sensing adaptor, that sequesters this CNC-bZIP factor in the cytoplasm (by binding its Neh2 domain) and enables its ubiquitination by the Cul3-Rbx1 E3 cullin-RING ligase (CRL3) complex, before being degraded by the proteasome-mediated protein disposal system. However, no similar negative effects of Keap1 appeared to be exerted on Nrf1, even though the putative Keap1-binding Neh2L domain is also represented in Nrf1 303. This is attributable to the fact that this Neh2L domain is translocationally buried in the lumen of ER, such that Nrf1 is hard to gain access to the extra-ER subcellular Keap1 275 304. Once the Neh2L domain of Nrf1 is retrotranslocated across ER membranes to enter the cytoplasmic compartments, it is allowed for binding Keap1 and stabilized by this redox-sensing adaptor, which regulates Nrf1 distinctively from Nrf2 417, but such stabilization of Nrf1 by Keap1 is hardly validated in our experimental settings 418 419. In turn, it was, to our surprise, found that specific KO of human Nrf1α (i.e., *Nrf1*α*^-/-^* with its long isoform TCF11) led to the substantial diminishment of Keap1 in HepG2 cells, while human *Nrf2^-/-^* (with a genomic deletion of its transactivation domains) caused an obvious increase in basal Keap1 expression, when compared with wild-type controls 265 419. This finding demonstrates a bidirectional positive and negative regulatory profile of Keap1 by Nrf1α and Nrf2, respectively. Such positive and negative regulation of Keap1 by Nrf1α and Nrf2 is likely related to its binding partner p62-driven autophagy and its subsequent lysosomal degradation of this relatively long-lived Keap1 420 421 422 423. The p62 (also called sequestosome 1, SQSTM1) is transcriptionally induced upon oxidative stress by Nrf2 by direct binding to the ARE sequence in the p62 promoter 424. Once p62 is accumulated, which also occurrs when autophagy is impaired, the increased p62 enables direct binding to Keap1 and also targets it to the autophagy-mediated lysosomal degradation, resulting in a reduced proteasomal turnover of Nrf2 via such a feed forward loop within chronic redox stress signaling. By contrast, this autophagy adaptor p62 expression was basally enhanced in *Nrf1*α*^-/-^* cells, implying that it is negatively regulated by Nrf1α 420, but the detailed mechanisms remain to be elucidated.

The autophagy and ubiquitin-proteasome systems are two primary cellular pathways of misfolded or damaged protein degradation to maintain intracellular protein homeostasis (proteostasis). When the proteasome is dysfunctional, cells compensate for so impaired protein clearance by activating selective autophagy to eliminate ubiquitinated or damaged protein aggregates. In studying both cross-talking mechanisms, it was found that a rapid, dramatic and selective induction of *GABARAPL1* (but not other autophagy genes) and *p62* (bridging ubiquitinated proteins with GABARAPL1 on autophagosomes) was caused by proteasome inhibitor treatment for 4 h, to promote cell survival before autophagy activation 425. Conversely, knockdown of p62 or GABARAPL1 reduced cell survival upon proteasome inhibition. Such transcriptional induction of p62 by Nrf1 enables enhanced survival primarily by sequestering ubiquitinated proteins in nuclear inclusions and perinuclear aggresomes 425, albeit simultaneously accompanied by induced transcriptional expression of proteasome genes (i.e., proteasome bounce-back response 340 426). The transcriptional activation of aggrephagy by Nrf1 in the response to proteasomal dysfunction is further corroborated by autophagy-related *p62/SQSTM1* and *GABARAPL1* (an ATG8 family gene) directly targeted by this CNC-bZIP factor 427. Interestingly, Nrf1 was also identified to be indispensable for the formation of p62-positive puncta and their co-localization with ULK1 and TBK1; both kinases may activate p62 via phosphorylation, because knockdown of Nrf1 caused substantially reduced phosphorylation of p62 at Ser403 427. Such selective upregulation of GABARAPL1 by Nrf1 also facilitates induced clearance of ubiquitinated proteins, particularly whilst proteasome is impaired 427. Rather, a prolonged treatment of proteasome inhibitor for 20 h, these cells activated autophagy and expression of most autophagy genes possibly by an Nrf1-independent mechanism 425, although this CNC-bZIP factor is recently further confirmed to regulate similar proteotoxic stress-induced autophagy 428.

The conserved proteasome bounce-back response was originally observed in *S. cerevisiae* 429 430 431, where chemical or genetic inhibition of proteasome activity induces new proteasome synthesis promoted by the stress-regulated transcription factor RPN4 (with its DNA-binding C2H2 zinc-finger zipper domain ^461^RHQNTIHAKRKIVFRCSECIKILGSEGYQKTFSRLDALTRHISKHEDLSLEQRQEVTK^520^), so to ensure that the proteasome capability is matched to changing proteolytic requirements. Another similar transcriptional feedback loop was also found to exist between the proteasome and SKN-1 in *C. elegans* 432, revealing activation of SKN-1 by proteasomal dysfunction is tied to the protein-degradation machinery of the cells and also produces a selective oxidative (proteotoxic) stress response. The increased expression of 20S proteasomes was further validated to be de facto mediated by SKN-1 and CncC in both worms and flies, respectively 433. Such compensatory recovery of proteasome activity induced by limited proteasomal inhibition was also observed in mammals 434 and identified to be predominantly activated by Nrf1 (and its long isoform TCF11, but not Nrf2) via an ERAD-dependent feedback loop 340 426. Under non-inducing conditions, Nrf1/TCF11 within and around ER membranes is targeted to the ERAD requiring E3 ubiquitin ligase Hrd1 and the AAA ATPase p97. Upon exposure to a lower dose of proteasome inhibitors, this causes an accumulation of oxidant-damaged proteins and then promotes the nuclear translocation of Nrf1/TCF11 from the ER, before permitting activation of proteasome gene expression by binding to AREs in their promoter regions. The Nrf1-driven transcriptional feedback loop regulating all those proteasomal subunits and their co-factors, as well as p97, was further confirmed in other groups 435,436. Overall, Nrf1/TCF11, rather than Nrf2, serves as a key essential regulator for activation of the highly conserved proteasome bounce-back response for 26S proteasome formation to compensate for its reduced proteolytic activity, and mediate its innate cytoprotective response to counteract proteotoxic stress caused by proteasome inhibition.

The autophagy-lysosome pathway activity is enhanced by graded proteasome dysfunction caused by a mutant of RPN10 in *C. elegans* 437, in which enhanced resistance to aggregation prone proteins depends on autophagy genes atg-13, atg-16.2, lgg-1, bec-1 and prmt-1, though the animals are particularly sensitive to the inhibition of lysosome activity via either RNAi or chemical means. Such moderate proteasome dysfunction can be leveraged to improve proteostasis capacity, and organismal healthy survival and longevity through the activation of compensatory mechanisms regulated by SKN-1A/Nrf1 (and ELT-2/GATA), that mediates increased expression of the genes encoding proteasome subunits (as a conserved bounce-back response), as well as those mediating anti-oxidative and heat-stress adaptive responses 437 438. The proteasome regulation by SKN-1A/Nrf1 also enables innate immune responses to be kept in check in a tissue-specific way against natural pathogens of the *C. elegans*. Conversely, constitutive expression of immune response programs against pathogens was triggered by the loss-of-function mutants of SKN-1A and its activating enzymes DDI1 and PNG1, leading to proteasome inhibition 438.

In addition, the ER-anchored ubiquitin-specific protease USP19 acts as a novel mechanistic modulator of Nrf1, but not Nrf2, which directly interacts with Nrf1 near the ER and also topologically functions as a deubiquitinating enzyme to remove ubiquitin moieties from this protein, thereby enabling Nrf1 to be rescued from the putative ubiquitin-directed ERAD pathway 317. In turn, the transcriptional expression of endogenous USP19 and its promoter-driven reporter genes is differentially regulated by Nrf2, as well as by Nrf1, at distinct layers within a complex hierarchical regulatory network. Recently, it was discovered that Keap1 is a crucial multifunctional player in governing and/or maintaining robust proteostasis, because it cannot only contribute to the ubiquitin-mediated proteasomal degradation system by interacting with so many ubiquitination enzymes (including Cul 1 to 5) along with 26S proteasomal core and regulatory subunits, but also conversely enables for a novel contribution to relevant protein stability (e.g., Smad2/3) by an additional deubiquitination system 419.

#### Inter-regulation of Nrf1 and Nrf2 with differential redox signaling to cancer metabolism 

The aberrant accumulation of hyperactive Nrf2 was found to result from specific KO of human *Nrf1*α*^-/-^* HepG2 cells 265, which is attributable to substantially diminished expression of its negative regulator Keap1 and Nrf1-target proteasome dysfunction. Although Nrf2 is hyperactive, the malignance of *Nrf1*α*^-/-^*-leading xenograft tumor and metastasis were enhanced 265 266. Similarly, marked results were also obtained from the knockdown of all Nrf1 isoforms (*Nrf1-KD*) 413. Conversely, knockdown of Nrf2 caused *Nrf1*α*^-/-^*-derived tumor to be suppressed to a similar extent to wild-type controls (**Figure 13A**). By sharp contrast, the inactive Nrf2 enabled *Nrf2^-/-^*-leading tumor to be dramatically repressed and almost completely abolished in the model xenograft animals 265, but the constitutive active Nrf2 (*caNrf2*) expression did not result in a significant change in *caNrf2*-derived tumor, when compared to wild-type controls (**Figure 13A**). These observations demonstrate that Nrf2 is much more likely to act as a tumor promoter, whilst Nrf1 serves as a tumor repressor. This was evidenced by further experiments revealing that the remarkably tumor-repressing effects on hepatocellular carcinoma were convincingly corroborated by restored expression of ectopic Nrf1 or TCF11 factors 123. Therefore, it is inferable that the tumor-promoting effect of Nrf2 is stringently confined by Nrf1 as an indispensably braking control of Keap1 and proteasome functioning in distinct adaptive responses. Besides, the transcription expression of *Nrf2* was also shown to be inhibited by Nrf1, because its basal and inducible mRNA expression levels were up-regulated to varying extents in the tissue-specific *Nrf1^-/-^* mice 348 391, as well as in human *Nrf1*α*^-/-^* hepatoma cells 89 439 440 441. In turn, substantially reduced transcription of *Nrf1* was caused by *Nrf2^-/-^* deficiency, implying that Nrf1 is positively regulated by Nrf2, and also by itself as revealed by its promotor-driven reporter assays 265. Overall, such inter-regulation between Nrf1 and Nrf2 occurs at distinct levels from their gene regulatory transcription to post-synthetic protein disposal. This is also further corroborated by the *in vivo* mouse model expressing the Nrf1-MafG heterodimer, revealing that Nrf1 has the potential to activate canonical Nrf2 target cytoprotective genes when strongly induced 442.

Of crucial importance is severe endogenous oxidative stress caused by loss of Nrf1’s function, which cannot be compensated by hyperactive Nrf2 accumulated in the deteriorated *Nrf1*α*^-/-^* hepatoma cells 89 115 263 354 439 440. Similarly, oxidative stress was also observed in *Nrf1^-/-^* mouse model systems 172 261 326 397, and further aggravated by a combined deficiency of *Nrf1* and *Nrf2*, leading to fatal defects with typical pathologies 332 348. These demonstrate that Nrf1 acts as an indispensable determinon for robust redox homeostasis, albeit Nrf2 is accepted as a master regulon of antioxidant, detoxification and cytoprotective genes in this process 111 116. As such, the silencing of Nrf2 can rescue glucose deprivation-induced rapid death of *Nrf1*α*^-/-^* cells, because severe endogenous oxidative stress arising by its aberrant redox metabolism was ameliorated by knockdown of Nrf2, as a similar rescue was obtained from catalase 354. This fact indicates that the existing endogenous oxidative stress in *Nrf1*α*^-/-^* hapatoma cells is further augmented by hyperactive Nrf2, particularly upon glucose starvation, implying a possibly Nrf2-dependent (e.g., KLF9) pathway to stimulate increased production of ROS, as described by Zucker, *et al*. 443. Such switching of redox signaling by key modular molecules (e.g., GPX, PRDX) dictates cell fate decision by Nrf2-mediated dual opposing responses for adaptation or maladaptation, specifically when Nrf1 is lost. 

**Figure 14:**
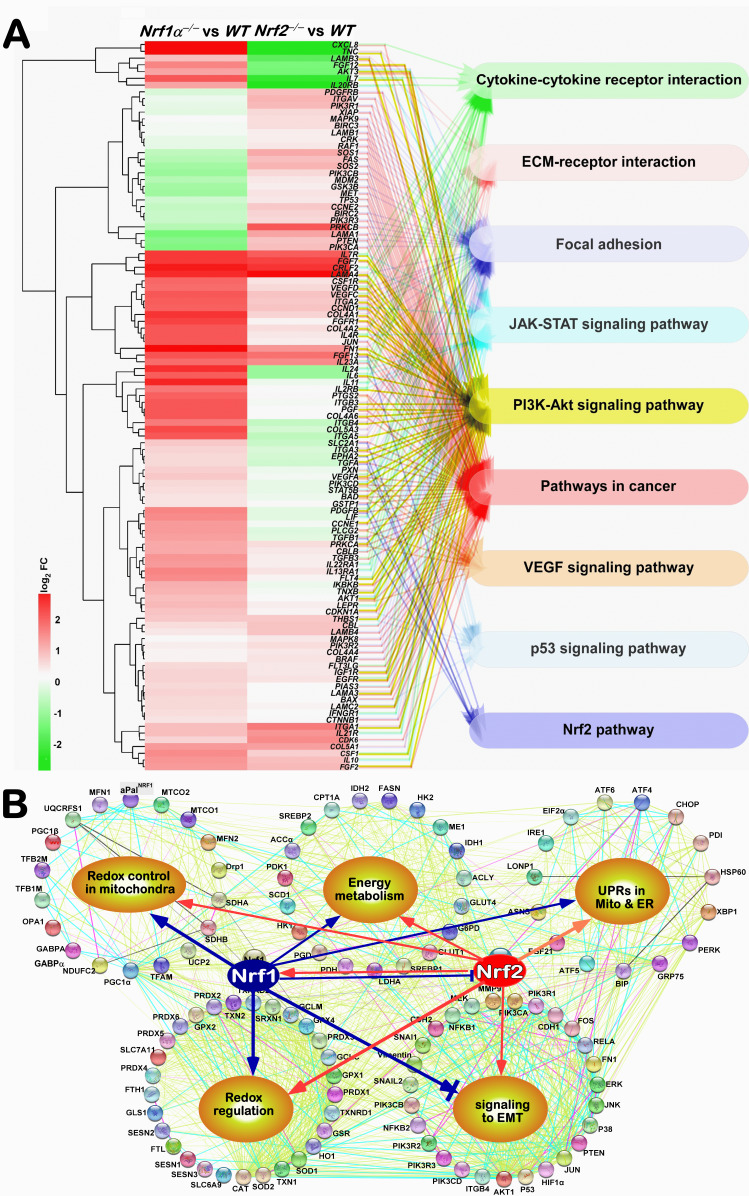
FIGURE 14: Differential but integral roles of Nrf1 and Nrf2 in regulating responsive genes. **(A) **A transcriptomic analysis of *Nrf1α^-/-^* and *Nrf2^-/-^* cell lines by comparison to wild-type (WT) cells. These differential expression genes (DEGs) were subjected to clustering their functional annotations into distinct signaling pathways, principally responsible for governing cancer development and progression. **(B)** The inter-regulatory roles of both Nrf1 and Nrf2 in uniquely yet coordinately controlling of redox signaling, energy metabolism and other target gene networks as indicated herein. These graphs with the relevant big data analyses were adapted from 115,123.

To defend against a vast variety of challenges from changing environments, an evolutionally selected set of antioxidants, detoxification and cytoprotective systems are predominantly regulated by Nrf1 and Nrf2 for their coordinated redox control to maintain cell homeostasis and organ integrity during healthy survival. Upon loss of full-length Nrf1α causes a dramatic increase in intracellular ROS level and oxidative damage in the resulting *Nrf1*α*^-/-^* cells, and this increase was not eliminated by drastically elevated Nrf2, although the antioxidant systems were also substantially enhanced by hyperactive Nrf2 115. Further experiments revealed that the increased ROS production by *Nrf1*α*^-/-^* resulted from a remarked impairment in the mitochondrial oxidative respiratory chain and its gene expression profiling regulated by two nuclear respiratory factors (called αPal^NRF1^ and GABP^NRF2^, which are non-homologous nuclearly-controlled transcription factors (Supplemental Figures S8 and 9) 115 364 418. In addition to the intrinsic antioxidant capacity of cells, aerburg effect) by aberrantly-elevated Nrf2, so as to partially relieve the energy demands of *Nrf1*α*^-/-^* cells, but heavily aggravate its mitochondrial stress (to yield an evident UPR^mito^, which is similar to, but different from, UPR^ER^ 89 115). Besides, the generation of ROS was also differentially regulated by Nrf1 and Nrf2 via the miR-195 and/or mIR-497-mediated UCP2 pathways 115. Upon glucose starvation of *Nrf1*α*^-/-^* cells, the altered gluconeogenesis pathway was greatly aggravated, also as accompanied by a weakened pentose phosphate pathway, as bad as dysfunction of serine-to-glutathione synthesis, leading to accumulation of ROS and severe oxidative damages, such that the intracellular reduced equivalents (i.e., GSH, NADPH, TRX) were exhausted 354. Thereby, it is inferable that glucose starvation leads to acute death of *Nrf1*α*^-/-^* , rather than *Nrf2^-/-^*, hepatoma cells resulting from its fatal defects in the redox metabolism reprogramming. This is also likely attributed to distinct requirements of Nrf1 and Nrf2 for regulating the constructive and inducible expression of those key genes involved in redox metabolic reprogramming in cancer development and malignance (**Figure 14**) 115 264.

#### Distinct regulation of Nrf1 and Nrf2 in cancer metabolism reprogramming

Of striking note, Nrf1 can serve as a dual sensor and regulator of glucose homeostasis, because glycosylation of Nrf1α enables it to sense the energy state 335. Dysfunction of this energy sensor leads to glucose metabolism reprogramming, so to markedly aggravating the Warburg effect in Nrf1-silenced hepatoma cells, as accompanied by the resulting mitochondrial damages. Such glucose reprogrammed effects are driven primarily by uncontrollable signaling mediated by AMP-activated protein kinase (AMPK) in Nrf1-deficient cells, because this CNC-bZIP factor negatively regulates this nutrient-sensing master kinase by direct physical interacting ‘placeholder’ 355. Of crucial importance, recent studies further revealed that the loss of Nrf1’ function also leads to lipid metabolism disorders, along with severe accumulation of ROS and lipids as deposited in lipid droplets of *Nrf1*α*^-/-^* cells 263 264. This is attributable to that the cellular lipid synthesis pathway was up-regulated by the JNK-Nrf2-AP1 signaling, whilst its lipid decomposition pathway was down-regulated by the nuclear receptor PPAR-PGC1 signaling. By sharp contrast, knockout of *Nrf2^-/-^* decreased lipid synthesis and uptake capacity. This indicates that Nrf1 and Nrf2 contribute to significant differences in the cellular lipid metabolism profiles and relevant pathological responses. Further studies uncovered that lipid uptake and ensuing deposition in *Nrf1*α*^-/-^* cells resulted from CD36 up-regulation by activating the PI3K-AKT-mTOR signaling pathway, consequently leading to an aberrantly activated inflammatory response 263 264. More interestingly, the yield of lipid droplets in *Nrf1*α*^-/-^* cells was strikingly alleviated by 2-bromopalmitate 263. This effect was also accompanied by substantially abolished expression of the endogenous CD36 and critical inflammatory cytokines. This finding provides a potential strategy for cancer prevention and treatment by precision targeting of Nrf1, Nrf2 or both. 

Metabolic reprogramming is accepted as a central hallmark of cancer and plays a pivotal role in malignant tumor occurrence, metastasis and even drug resistance 444 445. Thereby, it is inferable from the aforementioned findings that Nrf1 and Nrf2 are differentially and integrally required for the multifaceted crosstalk between redox responsive signaling and metabolic pathways to coordinately control a vast variety of the relevant signaling molecules and metabolic enzymes involved in cancer metabolism, aiming to sustaining robust redox homeostasis and metabolism homeostasis (metabostasis, including glucose, lipid, cholesterol and protein homeostasis. The in-depth insights unraveled that genetic deletion of Nrf1 and Nrf2 resulted in distinct metabolism reprogramming in human hepatoma cells 264. That is, the loss of Nrf1α de facto led to enhanced glycolysis, reduced mitochondrial oxygen consumption, enhanced gluconeogenesis and activated pentose phosphate pathway in the hepatocellular carcinoma cells. By contrast, the loss of Nrf2 attenuated the glycolysis and gluconeogenesis pathways, but without any significant effects on the pentose phosphatepathway. Such KO of *Nrf1*α*^-/-^* de facto caused fat deposition and increased amino acid synthesis and transport, especially serine synthesis 264, whereas *Nrf2^-/-^* deficiency did not cause fat deposition, but attenuated amino acid synthesis and transport. Such distinct metabolic programming between *Nrf1*α*^-/-^* and *Nrf2^-/-^* was further revealed to result from substantial activation of the PI3K-AKT-mTOR signaling pathway triggered by loss of Nrf1, leading to increased expression of those critical genes for the glucose uptake, glycolysis, the pentose phosphate pathway, and the *de novo* lipid synthesis, whereas deficiency of Nrf2 resulted in the opposite phenomenon by blocking this PI3K-AKT-mTOR pathway 264. This signaling downstream transcription factors HIF1 and SREBP1/2 were further corroborated as two key players in such distinct metabolism reprogramming of between *Nrf1*α*^-/-^* and *Nrf2^-/-^* cells, just as described previously by 446 447. Of note, *Nrf1*α*^-/-^*-enhanced activation of the HIF1 signaling led to increased expression of the glycolysis rate-limiting hexokinases HK1/2 and glucose transporters (e.g., SLC2A1, also called Glut1) 264 354 355. 

In addition to the significantly increased uptake of lipids by CD36 263 347, the loss of *Nrf1^-/-^* resulted in enhanced *de novo* lipid synthesis by activated SREBP1/2 through the PI3K-AKT-mTOR pathway 264. Of crucial importance, Nrf1 acts as a direct ER membrane sensor critical for cholesterol and lipid homeostasis through SREBP1/2 and LXR 347. Such cholesterol-dependent homeostasis can also further finely tune the regulation of very long chain sphingolipid synthesis to maintain plasma membrane lipid homeostasis and cell integrity 448. Together, these indicate the Yin-Yang relationship between Nrf1 and SREBP1/2 for sustaining cholesterol and lipid homeostasis. Another recent report by Xu’s group showed activity of SREBPs is inhibited by promoting degradation of SREBP-cleavage activating protein (SCAP, a canonic cholesterol sensor) through the ubiquitin E3 ligase RNF5-dependent proteasomal pathway, the ligase is recruited to a direct target of Nrf1, i.e., the ER-resident transmembrane TMEM33 449. The TMEM33 serves as a downstream effector of pyruvate kinase isoform 2 (PKM2), which coordinates together with p97/VCP to tightly control the selective processing of Nrf1 and SREBPs, and their bidirectional regulatory capability to dictate lipid metabolism and homeostasis 449. Because Nrf1 and SREBP1 manifest distinct topogenetic behaviors around membranes 114 [114], both should be endowed with their disparate topovectorial spatiotemporal partitioning to exert their respective unique biological functionality, which occurs only after being dislocated from the ER and Golgi into the nucleus to gain access to cognate target genes (**Figure 10**). These demonstrate that Nrf1 is indeed contributable to the negative regulation of lipid metabolism and homeostasis through the PKM2/p97-Nrf1-TMEM33-RNF5-SCAP-SREBPs axis. Collectively, these together with an additional experimental report 319 have convincingly confirmed that Nrf1 is not a direct target of SREBP1, albeit both are involved in the rapamycin-responsive signaling networks, and this resulting point cannot thereby support the relevant view of Manning’s work 450.

### Unique and overlapping roles of Nrf1 and Nrf2 in integrating multi-hierarchical signaling networks to genetic adaptive reprogramming

The considerable lines of the hitherto accumulating experimental evidence (including those described above) have unveiled the unique and overlapping roles of Nrf1 and Nrf2, as well as their differentially positive and negative regulatory effects, all of which are integrated in elaborately governing cellular homeostasis and organ integrity and tightly persevering their robust status within certainly presetting physio-pathological threshold ranges. Such robust intrinsic status should be determined by unifying all relevant endogenous molecular-cellular-organismal networks, but also shaped by switching their physio-pathological functionality 179 451. Therein, Nrf1 plays a predominant, indispensable role, albeit Nrf2 also acts as a master regulator, in determining such robust physio-pathological states. This notion is solidly based on the gene-targeting experimental evidence revealing those unique pathophysiological phenotypes of Nrf1 but not Nrf2, as evinced in those distinct tissue-specific knockout mice (see section "Unique pathophysiological phenotypes of Nrf1 that are distinctive from Nrf2"), as well by their specific knockout cell lines from the human hepatoma (**Figure 13A**). As such, double KO of both *Nrf1* and *Nrf2* in mice also results in worsened pathological phenotypes, as compared to those of the single *Nrf1* KO mice 332 348. From these, it is inferable that such unique and overlapping biological roles of Nrf1 and Nrf2 are fulfilled by integrating all multi-hierarchical signaling networks into genetic adaptive reprogramming.

By analysis of the chromatin immunoprecipitation (ChIP)-sequencing combined with RNA-sequencing data, 31 differential expression genes (DEGs) were identified as co-targets of Nrf1, Nrf2 and Nrf3 (i.e., doxycycline-inducible FLAG-tagged Nrf1^ΔN1-121^, Nrf2 and Nrf3^ΔN1-173^ in the human osteosarcoma U2OS cell lines) 117. Among them, 18 genes were upregulated, plus nine genes down-regulated, by all three factors, apart from only four genes differentially regulated by at least one of their three. Additionally, 84, 84, and 22 genes were defined to be specifically up- or down-regulated by Nrf1, Nrf2, and Nrf3, respectively 117. By scrutinizing those ARE-containing ChIP peaks, it was unraveled that Nrf1 prefers its strictly binding to AREs flanked by AT-rich regions (e.g. to drive metabolism genes), but in contrast, Nrf2 prefers more loosely binding to canonical AREs adjoined by GC-rich regions 117. Furthermore, another overexpressing system of FLAG-tagged Nrf1^ΔN1-84^, Nrf2 and Nrf3^ΔN1-112^ in HEK293T cells were subjected to RNA-sequencing-based transcriptomics combined with quantitative proteomics to delineate their overlapping and differential genetic programs mediated by these three factors 452. Nrf1-specific targets were identified to include several chaperones (e.g., *HSPA4, HSPA8, HSPA9, DNAJC1, DNAJA2*), the chaperonin TCP complex (e.g., *CCT2, CCT5, CCT8*), and a set of genes for the heat shock response besides the proteasomal bounce-back response 452. This implies a crucial role of Nrf1 in cellular protein quality control by enhanced protein folding and increased proteasomal degradation to augment the proteostasis-based repair. However, it should be noted that these N-terminally truncated Nrf1 and Nrf3 were likely dubbed as two Cinderella artifacts of this CNC-bZIP family 453. Of importance, by evaluating the molecular function of the tethered Nrf1-MafG heterodimer by the mouse models, it was further unraveled that this heterodimer can *in vivo* activate the transcriptional expression of proteasome subunits and the proteostatic stress response genes (involved in ERAD, chaperone and ubiquitin-mediated degradation pathways) by specifically binding to ARE-related sequences in the proximity of these genes 442.

**Figure 15:**
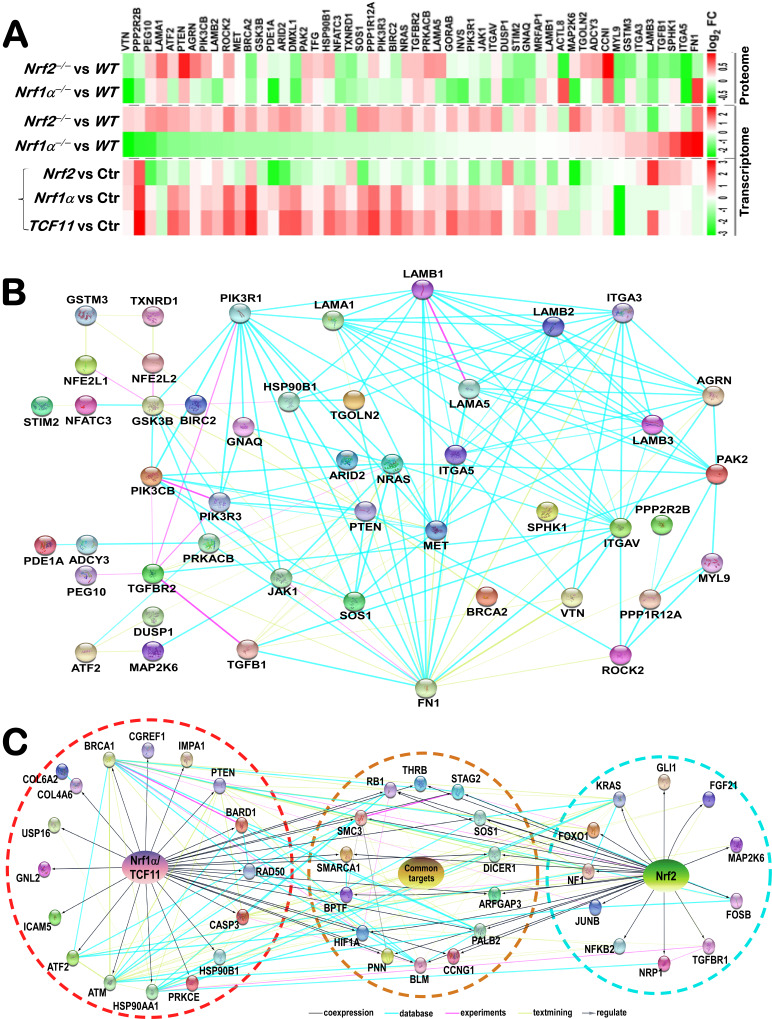
FIGURE 15: Distinctive or even opposing regulation of target genes by Nrf1/TCF11 and Nrf2. **(A) **Combined analysis of transcriptome and proteome by sequencing of two distinct cell model systems as indicated. Firstly, transcriptomic sequencing of *Nrf1α^-/-^* and *Nrf2^-/-^* cell lines, as well as the tetracycline-inducible HEK293 cell system stably expressing Nrf1, TCF11 or Nrf2, when compared to their corresponding controls. Secondly, proteome analysis of *Nrf1α^-/-^* and *Nrf2^-/-^* cell lines vs their control cells. The results revealed that differential or even opposing expression genes were regulated specifically by either Nrf1/TCF11 or Nrf2. **(B)** Such a specific gene regulatory network was bi-directionally governed by Nrf1/TCF11 and Nrf2. **(C)** Schematic representation of Nrf1/TCF11- or Nrf2-specific gene networks, along with their shared co-target genes, which were further selected as described previously 123.

It is noteworthy that the unique transmembrane-topogenetic behavior of Nrf1 makes its functions more complicated than Nrf2, such that alternative splicing of its transcript and the selective processing of its protein are enabled to yield distinct lengths of isoforms (e.g., TCF11, Nrf1α, Nrf1β, Nrf1γ) 123 178. The tetracycline-inducible stable expression of these isoforms, together with TCF11^ΔN2-156^ and Nrf2, were allowed on the base of the Flp-In T-Rex HEK293T system. The transcriptomic analysis revealed that, albeit Nrf1α and TCF11 have similar yet different regulatory profiles, both contribute basically to the positive regulation of their co-targets, which are disparate from those regulated by Nrf2 (**Figures 15C** and S10), whereas the mutant TCF11^ΔN2-156^ appears to resemble Nrf2 with the largely consistent structure and function 123 (**Figures 6C** and S10C). The disparity in such genes regulated by Nrf1 and Nrf2, along with those of TCF11, Nrf1α, Nrf1β and Nrf1γ, was validated by scrutinizing comprehensive functional annotation of their specific and/or common target genes (Supplemental Figure S10), as well as by the combined RNA-sequencing analysis of *Nrf1*α*^-/-^* and *Nrf2^-/-^* cell lines versus wild-type controls 123. Notably, Nrf1α/TCF11-specific genes are focused on nutrient uptake, cellular metabolism, protein folding, sorting and degradation, along with DNA replication and repair, whilst Nrf2-specific genes were heavily weighted in the developmental process 123 [123]. However, the opposing regulatory genes of 108 by Nrf1 and Nrf2 were clustered into pathways in cancer, including the PI3K-AKT, p53, VEGF and JAK-STAT signaling, and ECM-receptor interacting EMT process, aside from antioxidant and inflammatory responses (**Figure 14A**). From them, 52 genes were further selected by combined transcriptomic and proteomic analysis of Nrf1/2-indicated stably expressing and deficient cell lines (**Figure 15A**), to build a gene regulatory network (**Figure 15B**). A part of such distinct genes, by mapping with metabolome analysis (Supplemental Figure S11), were denoted on the cellular sphingolipid, inositol phosphate, glutathione and purine metabolisms, as well on the phosphatidylinositol (e.g., PI3K-AKT) signaling system. 

The above-described finding is consistent with our previously reported results, revealing the opposing effects of Nrf1 and Nrf2 on PTEN (phosphatase and tensin homologue) 265, which negatively regulate the intracellular levels of phosphatidylinositol-3,4,5-trisphosphate (PI3) and functions as an upstream tumor suppressor by negatively regulating PI3K-AKT-mTOR signaling (**Figure 13B**). Such negative and positive regulation of PTEN by Nrf1 and Nrf2, respectively, on the PI3K-AKT-mTOR signaling towards HIF1, AMPK and SREBP, led to an adaptive reprogramming of glucose and lipid metabolisms, along with certain amino acid metabolism reprogramming 263 264 354 355. In addition to PTEN, similarly opposing regulatory effects of Nrf1 and Nrf2 were executed on the p53 signaling 265 414, the WNT-β-catenin signaling pathway and the EMT process 266 413. Furthermore, Nrf1 and Nrf2 also contribute bi-directionally to the coordinated control of arachidonic acid metabolism enzymes COX1, COX2 and ALOX5 to confine the inflammatory stimulation (**Figure 13B**) 265. Once loss of *Nrf1*α*^-/-^*, uncontrollable inflammatory response was spontaneously aroused, leading to NF-κB signaling activation and increased expression of inflammatory cytokines, and accompanied by Nrf2-leading JNK-JUN (AP-1)-mediated stress response, along with aberrant accumulation of ROS and lipids 115 263 265. The EMT process of *Nrf1*α*^-/-^* cells was further activated by those putative ROS-stimulated signaling pathways via MAPK, HIF1α, NF-κB, PI3K, and AKT, all players involved in cancer development and progression. As a collective consequence, the inflammatory malignant transformation into carcinogenesis and cancer progression should take place in the *Nrf1*-deficient cases, in which hyperactive Nrf2 acts as a potent tumor promotor, because malgrowth of the *Nrf1*α*^-/-^* -driven tumor in xenograft model mice was significantly suppressed by silencing of Nrf2 265.

**Figure 16:**
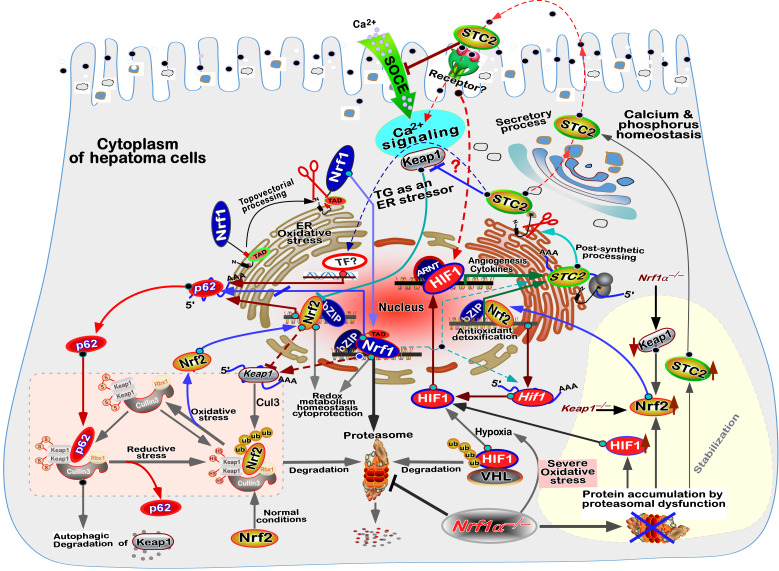
FIGURE 16: A model proposed for a better understanding of these key transcription factor inter-regulatory networks. Differential but integral regulation of Nrf1, Nrf2 and its target HIF1 signaling controls the biomarker STC2’s role in mediating distinctive phenotypes between *Nrf1α^-/-^* and *Nrf2^-/-^*. The distinction in their pathophysiological phenotype status is determined by such a robust endogenous molecular-cellular network. Conversely, STC2 can also serve as a potent feedback regulator of Nrf2 through Keap1 and/or p62. These critical protein expression levels are also tightly monitored by Nrf1-target proteasome and/or autophagy-lysosomal disposing systems, in addition to its transcriptional responsive reprogramming. For detailed explanations, see the relevant text and the cited reference 414.

To gain insights into the mechanisms underlying such distinct pathologic phenotypes of between *Nrf1*α*^-/-^* and *Nrf2^-/-^*, the transcriptome data from liver cancer in the TCGA database were also mined to establish a prognostic model and calculate predicted risk scores for each cell line. The results revealed that KO of *Nrf1*α markedly increased the risk score in liver cancer, while the risk score was reduced by knockout of *Nrf2* 414. In the prognostic model, stanniocalcin 2 (STC2), as a potential biomarker expressed highly in hepatocellular carcinoma tissues with a reduction in the overall survival ratio of those patients, was significantly upregulated in *Nrf1*α-deficient cells, but strikingly downregulated in *Nrf2*-deficient cells 414. The negative regulation of STC2 by Nrf1α is dependent on Nrf2 and HIF1α (as a direct upstream regulator of the STC2 transcription 454 455). Rather, the positive regulation of STC2 by Nrf2 may also be direct, independent of HIF1α (**Figure 16**). In turn, STC2 may regulate Nrf2 via a putative calcium-triggering Keap1-antagonized signaling pathway so to form a positive feedback regulatory circuit. Further studies unraveled that STC2, like Nrf2, functions as a dominant tumor-promoter, because STC2-leading increases in clonogenicity of hepatoma cells and malgrowth of relevant xenograft tumors were almost completely abolished in *STC2^-/-^* cells 414. This indicates that STC2 is also likely paved as a potential therapeutic target for liver cancer, albeit as a diagnostic marker.

### Differential yet integral roles of Nrf1 and Nrf2 in cellular homeostatic and adaptive responses

**Figure 17:**
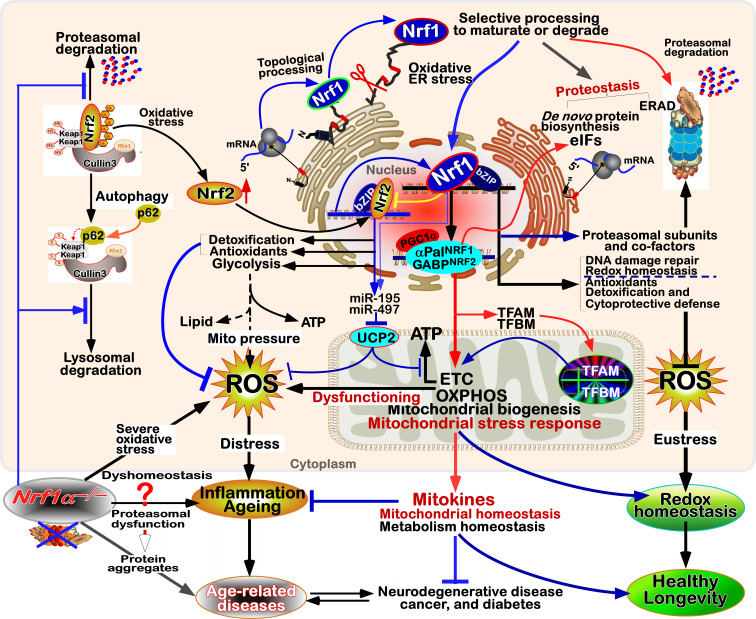
FIGURE 17: A proposed model for a better understanding of the major mechanism by which Nrf1 controls cell homeostasis. The membrane-tethered Nrf1 factor is selectively processed and hence activated to determine the cellular redox and energy metabolism homeostasis, as well as mitochondrial biogenesis, respiratory chain and its homeostatic integrity. Of importance, Nrf1 cannot only govern two key nuclear respiratory factors (αPal^NRF1^ and GABP^NRF2^) essentially required for mitochondrial function and homeostasis (see Supplemental Figures S8, S9 and S12), but also finely monitor the mitochondrial stress response by its protein quality control (i.e., UPR^mito^). Such unique biological functions of Nrf1 are distinguishable from those exerted by Nrf2, because the protein stability of Nrf2 and its activity are tightly confined by Nrf1. However, once the unique functions of Nrf1 are lost in its deficient cells or organs, the resulting dyshomeostasis of redox, glycose, lipid and protein metabolism, as bad as mitochondrial dysfunction with severe endogenous oxidative stress, could ultimately lead to unresolvable inflammation and malignant transformation into cancer, even rapid aging (e.g., non-infectious inflammatory ageing) and age-related neurodegenerative diseases. This graph was partially adapted from [REF]. In addition, it should be noticed that two non-homologous nuclear respiratory factors (also abbreviated often as NRF1/2) were confused with another symbols Nrf1/2 abbreviated from the nuclear factor, erythroid 2-related factor 1/2, and thus designated αPal^NRF1^ and GABP^NRF2^ (see Supplemental Figure S8, as discussed in references 364,418).

Upon exposure of cellular life to its homeostasis-threatening stress, particularly redox stress, a series of intrinsic anti-redox cytoprotective mechanisms (that have been established and developing in its eco-evo-devo possess of life histories) will be roused and activated to defend against such challenging stress in order to give rise to adaptive and homeostatic responses, to promote cellular resilience and maintain its physiological homeostasis. In such responses (i.e., CHR and CSR, as defined by Kültz 124), Nrf1 plays essential, predominant, and indispensable roles for CHR, whilst Nrf2 is involved in, but not essential for, the CHR, even although it is required for the cellular stress-adaptive response (CSR). This notion is concluded based on their distinct time-dependent responses driven by inducibly expressing Nrf1 and Nrf2 with disparate half-lives (**Figures 6** and S2) and a house of the hitherto accumulated experimental evidence (as described above in the relevant sections). As such, although Nrf1/TCF11 exerts an irreplaceable and pivotal role in governing the CHR, Nrf2 in handling such a rapid emergency response and CSR also provides an invaluable way of ‘buying time’ for the transition to the lagging CHR mediated by Nrf1/TCF11 insomuch as to coordinately cope with redox stress threatening cell homeostasis and organ integrity of life systems (**Figure 17**).

Once stimulated by stress, besides direct reactions, an acute emergence response is rapidly roused by sensing such stress signal transduction to redistribution of oxygen, nutrients and energy, followed by short-term responsive metabolism reprogramming and ensuing long-term adaptive genetic reprogramming to restore its original normal homeostasis or gain an adaptive or even maladaptive tolerance. This depends on the severity of such cellular stress and overall capacity of its intrinsic cytoprotective responses (**Figures 5** and **6**). In these cellular homeostatic and adaptive responses, both Nrf1 and Nrf2 fulfill their differential, yet integral, physiobiological roles by governing all relevant multi-hierarchical cellular-molecular signaling networks, in distinct topovectorial and right tempospatial ways, to shape adaptive metabolic and genetic reprogramming (**Figures 14B, 17** and S12).

The cellular redox homeostasis is determined by balancing between production of all redox equivalents (*R^o^_p_*) and their elimination (*R^o^_e_*) by relevant anti-redox defense system, as formulated by Δ*H* = *H_S_* - *H_0_* = ∫[*R^o^_p_* - *R^o^_e_*]×*k*, (in which *H_0_* and *H_S_* represent basal redox homeostatic and stress-stimulated redox robust states, respectively). A considerable number of substantial researches, as aforementioned, have revealed that differential yet integral roles of both Nrf1 and Nrf2, in governing production of all redox equivalents and their elimination, are exerted just as two entangled ‘Yin-Yang’ defensors to coordinately regulate the anti-redox and cytoprotective transcriptional responses insomuch as to maintain and perpetuate robust redox homeostasis within a certain presetting threshold range (**Figures 3** and **11A**). Therein, Nrf1 has been identified to act as a robust indispensable determinon for cellular redox homeostasis (and mitochondrial homeostasis) in particular normal physiological conditions, whereas Nrf2, as a master regulon, plays a versatile role in this process, specifically mediating adaptive hormestic effects induced by stress. Of note, the loss of Nrf1’s function leads to severe oxidative stress with obvious pathological phenotypes, but also was not compensated by hyperactive Nrf2 in *Nrf1*-deficient mice and cell lines 115 265 332 348. Such being the case, the permanently hyperactive Nrf2 is much more likely to give rise to another reductive stress 416 440. Altogether, these may enable an additional novelty maladaptive redox homeostatic status to be ultimately created, as a collective consequence, leading to the pathogenesis of cancer and other degenerative diseases.

By brown adipocyte-specific Nrf1 KO mice that cannot acquire cold adaptation by rising thermogenic activity, Nrf1 was identified as a master regulator of thermogenesis by regulating the transcriptional expression of UCP1 373. Another uncoupling protein UCP2 was differentially regulated by Nrf1 and Nrf2 via miR-195/497 115. This implies that Nrf1 and Nrf2 have differential and even opposing or combinational roles in governing cellular energy metabolism homeostasis (i.e., *I_ntake_* - *E_xpenditure_* = *S_torage_*), as evidenced experimentally by gene-manipulated model mice and cell lines 263 264 344 347 348 354. For instance, the energy uptake was controlled by Nrf1-driven transcriptional expression of glucose transporters (e.g., Glut1/2) 264 354 and lipid transporter (CD36) 263 347. The energy storage utilizing fat mass and glycogen was also affected by the presence or absence of Nrf1. The reduced glycemia in hepatocyte-specific *Nrf1^-/-^* mice resulted from reduced levels of the hepatic glycogen and its glycogen synthase *GYS2*, together with decreased insulin and IGF1 356. Intriguingly, two similar yet different lean phenotypes with lowered body weights but not fat mass were manifested in skeletal myocyte-specific *Nrf1^-/-^* mice 381 and also in its constitutive active *Nrf1-Tg:MGRD* mice 329. However, a large amount of fat mass is markedly stored in those liver-specific *Nrf1^-/-^* mice 261 347 348 and cell lines 263 264. The energy expenditure was also altered by adaptive reprogramming of cellular glucose and lipid metabolisms, controlled oppositely by Nrf1 and Nrf2, through the PTEN-controlled PI3K-AKT-mTOR signaling cascades toward HIF1/AMPK and SREBP1/2-mediated transcription responses, respectively 264. Besides, Nrf1 displays as a direct energy sensor to negatively regulate AMPK, a master kinase regulator of glucose metabolism reprogramming 355. Overall, these demonstrate that Nrf1 can also function as a robust determining factor for energy metabolism homeostasis (including glucose, lipid, and nutrient homeostasis), in addition to the aforementioned redox homeostasis and proteostasis.

In mediating adaptive transcriptional responses, Nrf1 and Nrf2 are indeed differentially and integrally activated upon stimulation by distinct types of cell stress, e.g., *tert*-butylhydroquinone (tBHQ, a pro-oxidative stressor) 439, dithiothreitol (DTT, a reductive stressor) 440, two classic ER stressors tunicamycin (TU) 89 and thapsigargin (TG) 265 414, as well as cisplatin (a platinum-based alkylating agent type of the DNA-damaging anticancer drug) 441, besides limited proteasomal inhibitors and other agents as reviewed previously 87 167 173 174 315. In addition to antioxidant, detoxifying and cytoprotective responses, Nrf1 was also identified as a vital and irreplaceable player in mediating mitochondrial stress response (i.e., UPR^mito^) by the ATF4/5-CHOP signaling 115, as well in governing this organelle biogenesis and its homeostasis by two nuclear respiratory factors (αPal^Nrf1^ and GABP^Nrf2^) and another two mitochondrial transcription factors (TFAM and TFBM) (**Figures 17**, S8, S9 and S12). Importantly, it was discovered that Nrf1, rather than Nrf2, is essentially required for DNA damage repair response through the H2AX-XPC pathway 441, but hyperactive Nrf2 in the *Nrf1*α*^-/-^* cells manifests a strong correlation with its chemoresistance to cisplatin, albeit their mechanistic details remain elusive.

## ACTIVATION OF NRF1 IS EVOKED BY DISTINCT TOPOVECTORIAL REGULATORY MECHANISMS

### Activation of Nrf1 is dictated by its topogenetic folding and retro-translocation across membranes 

**Figure 18:**
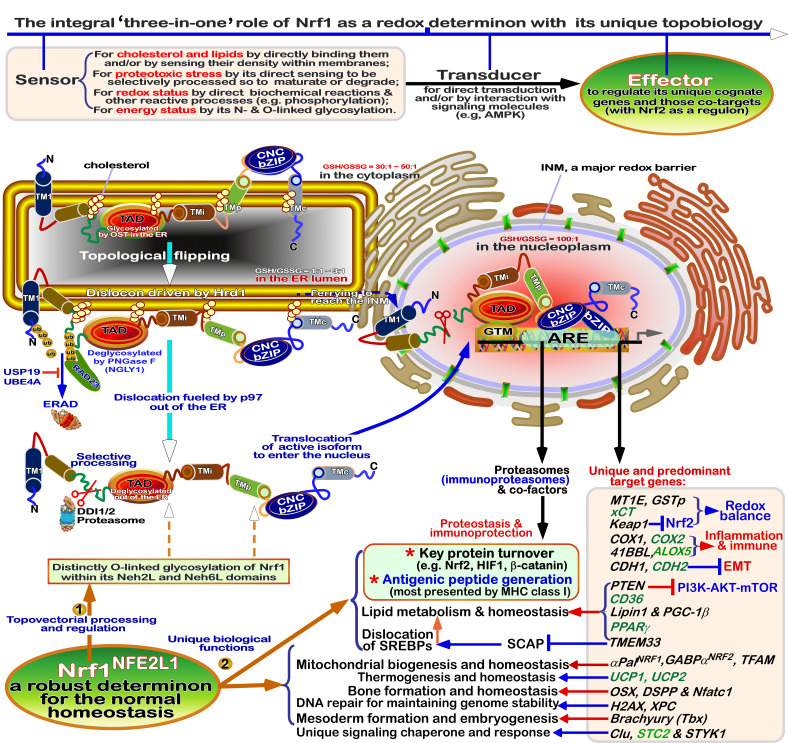
FIGURE 18: Nrf1 acts as a robust determinon for cell homeostasis by integrating its ‘three-in-one’ roles in topobiology. The **upper panel** shows that Nrf1 serves, by integrating its ‘three-in-one’ role, as a specific triplet of the direct sensor (for redox, energy, cholesterol and proteotoxic changes), signaling transductor and effector in the stress defense response to being challenged from changing environments. The **lower left panel** shows that the unique topogenetic folding of Nrf1 and its dynamic retrotranslocation repositioning across ER membranes dictate its topovectorial regulation by specific modification and selective processing to a mature active CNC-bZIP factor and other shorter isoforms, before regulating a particular subset of target genes, portion of which, as enlisted in the **lower right panel** (down-regulated genes in green, up-regulated genes in black), are responsible for fulfilling their unique biophysiological functions as deciphered herein.

As illustrated in **Figure 18**, the membrane-bound Nrf1 transcription factor is integrally anchored within and around the ER and hence conditionally sequestered by the membranes, because its TADs (including AD1, NST and AD2) are co-translationally positioned in the ER luminal side, whereas its DNA-binding CNC-bZIP domains are resided on the cytoplasmic side 112 203 275 304 305. Subsequently, the retro-translocational repositioning and selective proteolytic processing of the full-length Nrf1α (including its transcripts, as described in section "Unique topogenetics of Nrf1 and its dynamic dislocation across membranes to the nucleus before regulating target genes") can enable it to give rise to various lengths of its polypeptide isoforms with differential or even opposing functions, e.g., a mature N-terminally-truncated Nrf1^ΔN^ (and TCF11^ΔN^), relatively lowered active Nrf1β, along with a negative dominant Nrf1γ (**Figures 6C** and S1A) 123 178 307 312. Such unique topovectoral regulation of Nrf1 can also dictate specific post-synthetic modifications of this CNC-bZIP factor and its transcriptional activity to fulfill its unique physiobiological functions (as aforementioned in sections "Unique pathophysiological phenotypes of Nrf1 that are distinctive from Nrf2" and "Uniquely differentiated yet integrated roles of Nrf1 and Nrf2 in governing cell homeostasis during distinct life processes") 122 275 304 307 312. 

Such being the case, the membrane-topological regulation of Nrf1 by Hrd1/p97-driven retro-translocation of its glycosylated NST-adjoining TADs from the ER luminal to cytoplasmic sides of membranes 122 313 435 is finely tuned by the membrane lipid patterning, particularly within the ordered microdomains (composed primarily of cholesterol and sphingomyelin including ceramide). This is attributable to the presence of at least five cholesterol recognition amino acid consensus (CRAC) motifs within Nrf1, enabling this CNC-bZIP protein to sense changes in membrane constitutive cholesterol density and be also tightly anchored in those cholesterol/ceramide-enriched microdomains 122 347. Thus, it is inferable that dynamic topogenetic folding and subsequent retro-translocation of Nrf1 along with those functional domains being repartitioned, followed by its selective proteolytic processing to yield distinct isoforms, could all be confined by overloading cholesterol and/or ceramide in distinct topovectorial processes. However, the detailed mechanisms by which cholesterol and/or ceramide monitor such topobiological regulation of Nrf1 by the luminal-to-cytosolic retro-translocation of its glycosylated NST-adjoining regions (to become a real functional transactivation domain) remain to be elucidated, albeit the sphingomyelin metabolism is significantly affected by the loss of Nrf1’s function (Supplemental Figure S11).

### Activation of Nrf1 depends on its specific modification and selective processing

Firstly, activation of Nrf1 is induced by TU 89 456, which is generally accepted as a classic ER stressor, because it acts as a specific inhibitor of oligosaccharyl transferases (OSTs) catalyzing N-linked glycosylation of ER-resident proteins and thus results in unfolded or misfolded proteotoxic stress. Such induction of Nrf1 by TU is corroborated to yield enhanced expression of its non-glycosylated protein and facilitate its proteolytic processing and its ensuing dislocation from the ER to enter the nucleus before regulating the target gene. Increased transcriptional expression of Nrf1 at its mRNA levels is much likely activated by TU-stimulated PERK-Nrf2 and IRE1-XBP1 signaling pathways, as well as by Nrf1 per se 89 265 457 458. However, it should be noted that such TU-stimulated effects on Nrf1 were misinterpreted by a misleading pathway 341 (with a suspected attention paid by PUBPEER, Supplemental Figure S13), and also further misled by another short commentary on Nrf1 unleashed from the ER 459, based on the wrongly designed Nrf1’s topogenetic experiments 435 [435]. 

Secondly, activation of Nrf1 arises by its deglycosylation catalyzed by peptide: N-glycosidase (PNG-1, encoded by NGLY1), which occurs only after its ER luminal-resident N-glycosylated NST-adjoining TADs are dynamically retro-translocated across membranes to enter the cytoplasmic side 122 203 275 304 307 313. In addition to the removal of N-linked glycan from Nrf1, the deglycosylation of this factor by NGLY1 leads to the amino acid sequence reediting of its glycosylated asparagines to deglycosylated aspartates to potentiate its acidic transactivation capacity 275. This was convincingly corroborated by additional two laboratories working on Nrf1/Nfe2l1 and Skn-1 306 308. Conversely, N-glycosylation of Nrf1 and its transactivation activity are significantly suppressed by NGLY1-specific inhibitors 307 460 or its genetic loss of such function 461 462 463 464. Upon blockage of Nrf1’s deglycosylation, its glycosylated proteins are thus accumulated and abnormally ubiquitinated by the sugar-recognizing ubiquitin ligase SCF^FBS2^ in *NGLY1*-deficient cells, resulting in the retention of aberrantly modified Nrf1 in the cytoplasmic subcellular compartments before being subjected to its proteolytic degradation 465, this is accompanied by proteasome dysfunction and proteotoxic stress. Of importance, the *NGLY1* mutant patients are manifested with a congenital disorder of deglycosylation 464 466, leading to a congenital autosomal recessive disorder 467 and even death of adrenal insufficiency 462. Like brain-specific *Nrf1^-/-^* pathological phenotypes, *Ngly1^-/-^* animals develop neurodegenerative phenotypes and pathological abnormalities in the peripheral and central nervous systems 468. Similar to hepatocyte-specific *Nrf1^-/-^* cases, liver-specific deletion of *Ngly1* causes abnormal nuclear morphology and lipid metabolism, particularly under food stress 463. Collectively, these pathological phenotypes of *Ngly1*-deficient patients and animal models all result predominantly from the defective function of Nrf1, with a consequence of impaired proteostasis, accompanying proteotoxic stress-induced cell death, and down-regulated genes (e.g., *GCLC* and *GCLM*) responsible for glutathione synthesis, specifically in lymphoblastoid cells 469.

Thirdly, activation of Nrf1 is conferred by selective proteolytic processing of this CNC-bZIP factor by cytosolic proteases DDI1/2 or even activity-limited proteasome to yield a mature N-terminally-truncated active form Nrf1^ΔN^ (and TCF11^ΔN^) (**Figures 6C, 17** and **18**) 122 314 321 470. Of note, mammalian DDI2 is conserved with, but distinct from its yeast orthologous DDI1 in its structure and Function 471, which originally acts as a shuttling factor for proteasomes 318. For instance, DDI1/2 is indeed required for the removal of replication termination factor 2 (RTF2) from stalled forks by the proteasomal degradation pathway to maintain genome integrity 472. However, DDI1/2 contains a highly conserved retroviral protease domain that influences the directly binding of ubiquitinated proteins by its ubiquitin-like domain and proteasomal degradation 318. Thereby, selective proteolytic activation of Nrf1 by DDI1/2 or its turnover by proteasomes is dictated by its ER-trafficking configurations in distinct topovectorial phases 316. Such protease activity of DDI2 is required for controlling embryonic development and inflammation by Nrf1/TCF11 473, because of *DDI2^-/-^* mouse embryonic lethality at E12.5 with severe developmental failure, which resulted from insufficient proteasome expression with proteotoxic stress, and imbalance between innate immune and autoinflammatory responses. Furtherly, the activation of Nrf1 signaling towards the ubiquitin proteasome system by DDI2, together with its anti-redox response, also protects cells from ferroptosis 474 475. In the ‘bounce-back’ response of Nrf1 to sublethal proteasomal inhibitors, it is subjected to the selective proteolytic processing by DDI1/2 to yield an active CNC-bZIP factor, with its reduced protein degradation by activity-limited proteasomes 122 313 426. This is accompanied by enhanced protein expression of Nrf2 in such conditions, which enables the transcriptional expression of Nrf1 and Nrf1-target proteasomes to be augmented, albeit it is not a direct upstream regulator of the transcriptional expression of proteasomes.

Fourthly, Nrf1 is stabilized, to facilitate its transactivation activity to promote proteasome gene expression, by deubiquitination of Nrf1 by USP19 317, USP7 476 and/or UPS15 477 (which can also negatively regulate Nrf2 by deubiquitination of Keap1 478). Such deubiquitination of Nrf1 does not only rescue it from being targeted for the ERAD pathway, but also favors its subsequent selective proteolytic processing to yield an active CNC-bZIP factor that transactivates its cognate genes (e.g., proteasome and chaperones). Of note, it should also be noted that Hrd1 cannot only serve as a critical E3 ubiquitin ligase for ubiquitination of Nrf1/TCF11 313 426 479, but also functions as an essential retrotranslocon for this CNC-bZIP factor 480 481 482. Rather, whether or how the retro-translocation of Nrf1 in its ER-trafficking is affected by its deubiquitinating enzymes remains to be further elucidated.

### The transcriptional activation of Nrf1 in the defense response to distinct types of stress

In addition to directly sensing cholesterol, energy and proteotoxic stress (as described above), the membrane-tethered Nrf1 can also directly sense redox changes in between the most oxidizing ER lumen and relatively reducing extra-ER subcellular cytoplasmic and nuclear compartments by itself thiol-active groups, particularly at Cys^341^ and Cys^360^ residues (situated in its glycosylated NST domain and its DNA-binding basic region, respectively) 440. Such distinct types of stress are directly sensed and then transformed to various sorts of cellular signals, and also directly transduced to this effector of Nrf1 (albeit with an aide of Nrf2) in mediating the transcriptional response to regulate cognate target genes. That is, such integrated ‘three-in-one’ roles of Nrf1 are manifested as a highly-efficient triplet of specific sensor, transducer and/or effector in diverse stress defense responses (as illustrated in **Figure 18**, upper panel). This is dictated predominantly by the unique topogenetic folding of Nrf1 and its topovectorial regulation by dynamically retro-translocated repositioning of this CNC-bZIP factor from the ER lumen across membranes to enter the extra-ER compartments, before dislocating the nucleus, whereupon it regulates the transcriptional expression of a unique subset of antioxidant, detoxification and cytoprotective genes (**Figure 18**, lower right panel). 

In turn, the transcriptional activation of Nrf1 to enhance its mRNA expression levels is indeed triggered in such redox stress conditions, which is also regulated by Pitx2/3 and αPal^NRF1^ 418 483, Nrf2 and itself 265, as well as by other transcription factors rather than SREBP1/2 89 319. In addition, the transcriptional activation of Nrf1 is monitored by its heterodimerizing partners (e.g., sMafs, ATF4, Jun), as reviewed previously 87, and also by several transcriptional co-factors, e.g., CtBP2 (C-terminal binding protein 2) 484 and HCF-1 (host cell factor 1) 310 311.

## CONCLUDING REMARKS AND FUTURE WORK

From the holistic eco-evo-devo perspective, we have listed a house of evidence revealing that Nrf1 is a living fossil’s transmembrane transcription factor, that is closer than the water-soluble Nrf2, to their commonly-shared ancient orthologues (e.g., Nach, Cnc, Skn-1) emerged from distinct evolutionary stages of life histories. Such highly-conserved Nrf1 acts as a robust indispensable determinon, whilst Nrf2 serves as a versatile chameleon-like master regulon, in maintaining the cellular redox, energy and metabolism homeostasis and organ integrity within certainly presetting homeodynamic ranges. Conversely, the loss of such unique functions of Nrf1 in distinct tissues leads to obvious pathophysiological phenotypes, which are distinctive or even absent from the loss of Nrf2. Such distinctions between Nrf1 and Nrf2 are attributable to the unique topogenetic folding of Nrf1 and dynamic topovectorial repositioning of this CNC-bZIP protein by Hrd1/p97-driven retrotranslocation across ER membranes to dislocate the nucleus before transcriptional regulation of cognate target genes (**Figures 10** and **18**). In distinct topovectorial processes, Nrf1 is further endowed with specific reversible modifications (e.g., N-linked or O-linked glycosylation and deglycosylation, ubiuqintination and deubiuqintination) and selective proteolytic processing by cytosolic proteases DDI1/2 and/or limited proteasomes to yield an active N-terminally-truncated CNC-bZIP factor, with several shorter isoforms with distinctive or even opposing functions. Collectively, such uniquely differentiated yet integrated roles of both Nrf1 and Nrf2 are fulfilled in coordinately governing cell homeostasis and organ integrity during distinct life processes, in which both are manifested with their allelopathic potentials to virtually resemble two entangled ‘Yin-Yang’ quanta abiding by a dialectic law of the unity of opposites (**Figures 3, 11** and **17**).

Of crucial importance, the full-length Nrf1α is endowed to execute its essential physiobiological functions by integrating its ‘three-in-one’ roles, evinced as a specific triplet of the direct sensor (for redox, energy, cholesterol, and proteotoxic changes), signaling transducer and also effector in the stress defense response to being challenged from changing oxygenated environments (**Figure 18**). Such highly-efficient activation of Nrf1α is evoked by distinct topovectorial regulatory and processing mechanisms to mediate transcription responses by a subset of antioxidant, detoxification and cytoprotective genes against those inflammatory and degenerative diseases (e.g., cancer, ageing and aging-related neurodegenerative diseases). Therefore, this provides a potential strategy for chemoprevention and treatment of cancer and other degenerative diseases to be paved by precision targeting of Nrf1 alone or in combination with Nrf2 167 174. However, in this area there remains to address the following open questions: i) how the full-length mRNA transcript of *Nrf1* from its single gene is selectively processed to generate distinct lengths of isoforms; ii) whether the yield of distinct lengths of *Nrf1*’s transcripts is monitored by alternative 5‘- and/or 3‘ non-coding regions within its gene locus; iii) what mechanisms account for alternative translation of Nrf1 to enable for a selective yield of distinct length of polypeptide forms, and iv) what about distinct Nrf1 isoform-specific physio-pathological functions exerted *in vivo*? 

In addition, it should also be noticed that it was not identified which isoforms of Nrf1/Nfe2l1 exactly elicited another unexpected tumor-promoting role in very few of recently-reported, but not-yet-confirmed, cases 483,484,485. The putative promotion of triple-negative breast cancer was viewed to arise by Nrf1-mediating proteasomal cytoprotective response to resist against the proteasomal inhibitor treatments 485 and/or Nrf1-driving enhanced expression of programmed death ligand 1 (PD-L1) for its immune evasion 486. Ferroptosis of oral squamous cell carcinoma was also restrained by Nrf1-target holiday junction recognition protein (HJURP), leading to the cancer progression 487. As such being the cases, these tentative and preliminary investigations also need to be further substantiated by elaborate mechanistic studies. Nrf1 is also essentially required for antitumor immune response to block immune evasion by mediating the transcriptional expression of TNFSF9/41BBL (i.e., a tumor necrosis factor superfamily transmembrane cytokine that functions as a bidirectional signal transducer and another ligand for the costimulatory receptor 41BB in T lymphocytes 488). Conversely, the dysfunction of Nrf1 in either *NGLY1*- or *DDI2*-deficient cells and animals leads to activation of those immune-related genes by both cGAS-STING and MDA5-MAVS pathways, resulting in immune dyshomeostasis accompanied by inflammatory response 473 489. Similarly imbalanced immune activation and inflammation were unveiled in experimental autoimmune encephalomyelitis, which may be caused by a combination of proteasomal subunit displacement and reduced Nrf1 expression 490. More interestingly, the transcriptional expression of immunoproteasome (i-20S) and its activators PA28αβ (which function predominantly in the antigen presentation and regulation by γ-interferon, rather than in protein degradation) was substantially suppressed in the proteasomal inhibitor-stimulated ‘bound-back’ response mediated by Nrf1 436, even though not a canonical ARE sequence exists in these gene promoters. Altogether, such debating results presage a good start for this topic to explore the potential roles for Nrf1/Nfe2l1 in the immune homeostasis along with anti-tumor immune and anti-inflammatory responses.

Moreover, differential yet integral roles of Nrf1 and Nrf2 in redox regulation of physio-pathological functions determine distinct robust steady-states (i.e., health, subhealth, pathogenesis, and disease), that may quantitatively be stratified by extents of redox stress and anti-redox defense responses relying on the base of the ‘zero theory’ (at *P_0_*). Such distinct robust states are likely demarcated through different mechanisms accounting for homeostasis, morphostasis, homeorhesis, eustasis, allostasis and cacostasis (Δ*H* = *H_S_* - *H_0_*), along with relevant coding rules (e.g., redox code, stress-coping code, and topogenetic code), all selected by the nature eco-evo-devo process. Since the stress and redox stress should be parsed in terms of real scientific principles of physics and chemistry, oxidative or reductive stress with anti-redox responses can be determined by redox biochemical ontology in the development of cancer as a unicellular ‘oncoprotists’-like life form, but not as a simple disease only. This definition is just based on the law of Darwinian evolutionary dynamics of life from the holistic eco-evo-devo perspective 38. The robust state is further dictated by multi-hierarchical molecular-cellular-organismal signaling cascades towards metabolic networks, key gene regulatory profiling and their reprogramming 179. All these combine so to comprise a large complex endogenous ‘tensor’ network, which can be mathematically modeled with stochastic effects 491 492 and further precisely quantified by a set of equations for network dynamics, with nonequilibrium potential function approaches 493 494. For example, as two key transcription factors in governing cellular energy metabolic processes and homeostasis, Nrf1 and Nrf2 consist of a minimum regulatory network (**Figure 13B**). Hence, from a quantitative and dynamical perspective, a mathematical modeling can be set up. Of note, this minimum network is de facto embedded in a large complex network which can be viewed from two sides. It is a part of the large regulatory and signal transduction network, such as those extensively studied as endogenous networks 179 180, because in this review we have discussed this aspect reasonably well. Another one is its control of metabolic networks. One of us had discoursed in 2005 on its dynamics with various approaches 491, where a set of considerations were discussed. Then, we discussed how to handle realistic experiments view examples such as in the more controlled systems 495. After a series of efforts, the quantitative modeling was summarized 493. The dynamics of the corresponding endogenous network and metabolic network should be studied together, because biologically we know they influence each other, as explored in a combined study from the modeling side 496.

## ADDITION OF THE ESSENTIAL NOTIONS ON ‘GRAND REDOX-UNIFYING THEORY OF LIFE’ WITH MURBUM CONCEPT

**Figure 19:**
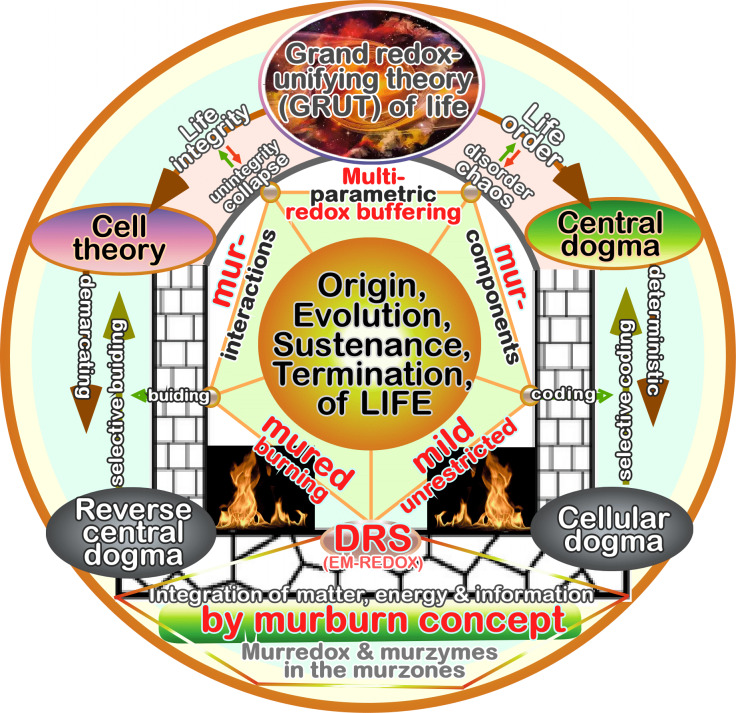
FIGURE 19: The Grand Redox-unifying Theory (GRUT) of life integrated with murburn concept and other coding rules. Similarly in physics, the GRUT of life kingdoms is established on the base of murburn concept. The ‘mur’ was, at five distinct layers, represented by: i) ‘mured burning; ii) mild unrestricted burning; iii) mur-components (including reactive molecules, unbound-ions and radicals) and/or radiations (transiently and/or incidentally generated) that comprise the redox system; iv) Stochastic molecule-unbound ion-radical interactions (i.e., mur-interactions) and v) multi-parametric redox buffering reactions, relevant mur-modifications and defensing responses to murredox stress. Herein, diffusive reactive species (DRS, including all reactive molecules, ions and radicals) are not commonly accepted as a series of deleterious villains, albeit they were early stigmatically perceived as chaotic damaging agents (and/or byproducts), in normal physiological and aberrant pathological states. Conversely, DRS serve de facto to function as versatile elixirs of life in all organisms that network almost all various bio-physicochemical, metabolic and physiological routines, particularly because they are obligate to be required for aerobic respiration and photosynthesis, xenobiotic metabolism and drug detoxification, and immune defensing system. In murburn processes, murzymes govern the generation of DRS and its functioning, modulation and even elimination within dynamic ecological microenvironments (i.e., murzones) of life’s origin and evolution. Such murburn-based GRUT is universal and fundamental accounting for all life forms. They, together with deterministic central dogma, extended cellular dogma, cell theory, and ‘reverse central dogma’, all are delved to gain a better understanding of life’s origin and evolution and its healthy sustenance with normal steady-state physiology and disease development. These are accompanied by a vast variety of coordinated/unified cellular physio-pathological functioning of life, in distinct vertices of bioenergetics (cellular powering and thermogenesis), coherence, homeostasis, electro-mechanics mobility, and sensing for signaling responses (PCHEMS) by electrophysiology (EM), redox biochemistry and quantum biology. Part of this figure adapted from 498,499,504.

The ‘murburn concept’, introduced by Indian biochemist Manoj, K.M. 497 498 499, represents a novel paradigm shift in understanding redox-integrated bio-physico-chemical sciences and its uniquely leading physiological roles ubiquitously implicated in all cellular life processes, including its origin, evolution and healthy sustenance with robust homeostatic physiology, as well as in even disease pathology leading to determination of life. As rooted in the interplay of diffusive reactive species (DRS, along with other mur-components), stochastic molecular reactions and interactions, and multi-parametric redox buffering (and relevant responses to redox stress), this framework challenges the traditional enzyme-centric models. Of note, those four key terms, i.e., murburn, murzyme, murzone, and murredox are integrated into electron-, proton- or moiety-transferred, redox-unified bio-physico-chemical mechanisms during life process. Here, we further review how this murburn concept bridges gaps from those existing theories (e.g., Cell Theory, Central Dogma and extended Cellular Dogma) to culminating in such a ‘Grand Redox-Unifying Theory’ (GRUT, based on physics’ unified field theory 500 501) accounting for all life kingdoms 502 503 (as deciphered in **Figure 19**).

Overall, the murburn-based GRUT redefines the emergence and evolution of all life forms by self-organized stochastic selection and variation of Nature from the primordial inanimate chemical worlds towards the animate metabolic world (of pre/pro-cells or LUCA) until the present-day ecological environments. This is also accompanied by autonomous sustenance of life in all organisms by self-regulated homeostasis and plasticity. Thereby, the intact topoform of life is maintained (and perpetuated) at a robust homeostasis status of normal physiological functioning determined by those coding principles. The long-term deterministic steady-state process is also accompanied by a series of dynamic nonlinear equilibria of all transient stochastic reactive and/or interactive bio-physico-chemical (murburn) processes, with varying extents of certain non-equilibrium fluctuations e.g., of between the integrity of life process by its topogenetically-ordered patterning activity and even its chaotic collapsibility.

### A paradigm shift by murburn concept bridging physics, chemistry, biology and even societal systems

The ultimate quest of life sciences is to unify the principles governing almost all those phenomena from quantum fluctuations to ecosystems, and from the origin of life to disease evolution, and even senescent death. In traditional molecular biology, the ‘Central Dogma’ of (DANN→ RNA→ protein) devised by Francis Crick 505 and the ‘structure determines function’ paradigm have long dominated life sciences for nearly a century. However, critical limitations of these frameworks have been gradually unveiled by the hitherto accumulating evidence from the origins-of-life chemistry, redox biology, and systems theory, and remain unresolved by the Cellular Dogma proposed by Quake 506. This is just owing to their failure to explain the structure-function decoupling with phenotype plasticity, the nonlinear inheritance of epigenetic memory (i.e., epigenetic dominance), membrane-mediated topogenetics with reverse self-regulation (e.g., by mur-modifications), DRS redox-driven (prebiotic paradox) origin and evolution of life, and/or societal system incompatibility. 

As deciphered in **Figure 19**, the GRUT of life as another revolutionary cross-scale framework is proposed on the base of murburn concept functioning as a stochastic fundamental principle, by integrating DRS-mediated nonlinear redox dynamics, topogenetics with incrementing information theory, and the ‘Reverse Central Dogma’ (as partially described in the aforementioned sections). Under this GRUT perspective, it is important to note that DRS-mediated murredox reactions (and modifications) particularly during the redox-driven life-historical processes, governed by putative ‘Reverse Central Dogma (information → function → structure, as coincided with the law of incrementing functional information 58 and also formalized by the Darwin-Ao equation with the evolutionary law of natural selection 38, represent the universal driver of life’s emergence, evolution, and even societal analogies. 

By integrating experimental data from prebiotic chemistry, ensuing topological genetics and epigenetics, along with cross-disciplinary systems analysis, it is inferable that the 3.8-billion-year trajectory of life is fundamentally a redox-programmed information topology. In such long life histories, DRS-leading networks encode spatiotemporal orders and coherent patterns through redox potentials (and/or redox signaling), guide molecular self-organization altogether, and extend their cognate inherent biologics to socioeconomic dynamics. Thereby, the murburn-based GRUT does not only redefine its essence of life, as ‘a self-sustaining redox-leading information continuum’, but also establish a unified principal framework bridging physics, chemistry, biology, bioeconomics and even social sciences. This arises just from DRS-redox drives of life’s origin to topogenetic information-function-structure evolution.

### Redox (and murredox) dynamics as life’s primordial engine to topogenetic building

In simulated alkaline hydrothermal vents (H_₂_/CO_₂_/Fe^³⁺^), DRS (i.e., H_2_S and HS• radicals) catalyzes acetyl-CoA formation via reductive carbonylation at 10^8^ M^⁻¹^s^⁻¹^ 507 and primitive amino acid synthesis 508. Such mon-enzymatic proto-metabolism appears to surpass enzymatic rates constrained by diffusion limits. The prebiotic Lipid-peptide vesicles self-assemble under redox-led *Eh* gradients, achieving proton motive force (Δ*p *= -120 mV) and ATP synthesis (3×10^5^ molecules/vesicle/hr) prior to genetic systems 509 510 511. In DRS-coherent networking, redox bistability was manifested by the fundamental Fe^²⁺^/Fe^³⁺^ redox cycles with a periodicity driving pH and *Eh* oscillations in prebiotic micro-compartments, enabling synchronization of proto-metabolic pathways 512 513. The CoQH• semiquinone radicals in mitochondrial Complex III oscillate at 10 Hz, matching ATP synthase rotation to optimize energy coupling 514 515. These processes are all explicable by DRS-based murburn concept 498 499 516 517 518.

By molecular self-assembly rules, lipid vesicle morphology to be spherical or tubular is dictated by Fe^²⁺^/Fe^³⁺^-modulated interfacial tension (γ = γ_₀_ + αΔ*Eh*, α = 0.3 mN/m·mV^⁻¹^) 519 520 521. Microtubule curvature (κ) responds to cysteine redox state: κ = κ_₀_ + β(^[GSH]^/_[GSSG]_), β = 0.02 nm^⁻¹^ per log unit 510 522 523 524. Similarly, zebrafish embryogenesis establishes ROS gradients (H_₂_O_₂_: 50 nM anterior ↔ 200 nM posterior), oxidizing VegT mRNA-binding proteins to pattern body axes 525 526 527. Plant root stem cell niches are demarcated by peroxisomal O_₂_^·⁻^ gradients, overriding classical morphogen diffusion models 528 529. Overall, self-organized morphogenesis arising from primitive lipid-peptide vesicles to diverse morphological topoforms of life is selected and evolved by redox dynamics to specify their presetting topogenetics spatial codes.

### The ‘Reverse Central Dogma’ validated empirically from the redox proto-membranes to genes

In addition to the definition of ‘Reverse Central Dogma’ (ancestral peptide minimotif → nucleotide template i.e., early RNA/DANN→ DNA, as described in the section "The peptide minimotifs evolving from primitive templates to specific DNA-binding transcription factors"), a bulk of empirical evidence has revealed coevolution of those primordial peptides and proto-membrane lipids during life’s origin to give rise to the LUCA. For instance, the H^⁺^-conductive vesicles (serving as a proto-membrane redox channel) were constructed by coevolution of C10 fatty acid-glycine lipopeptides under *Eh* = -200 mV, with the proton flux (*J_H^⁺^_*) obeying *J_H^⁺^_* = *J_max_* (1-e^-ΔEh/V0^), *V_0_* = 50 mV) 93 510 530 531 532. Mn^³⁺^-catalyzed peptide templating achieves 85% fidelity via redox-stabilized β-sheet interfaces following *F* = 1 – e^-k[Mn^⁴⁺^]t^, *k* = 0.03 μM^⁻¹^s^⁻¹^ 83 271 533 534 535 536. Such limited fidelity of peptide minimotifs has led plausibly to a vast variety of biological diversity in the Earth's biosphere.

The first nucleic acid strand could emerge via redox-dependent optimization 271 536 537 538. In this process, the RNA-peptide synergy was revealed by the experiment showing that archaeal RNase P activity requires redox cycling of Cys residues in RPP21 peptide; Cys→ Ser mutation reduces tRNA processing efficiency by 90% 539 540 541 542. The Fe-S clusters in DNA methyltransferases (DNMT3A) modulate methylation kinetics: *k_meth_* = *k_0_*[Fe_₃_S_₄_]^⁺^/(1 + [O_₂_]/*K_d_*), *K_d_* = 2 µM 543 544. Such redox epigenetic inheritance was also supported by reducing DNMT3A’s Fe-S cluster upon Hypoxia (*p*O_₂_ < 5 mmHg), hence lowering methylation rates by 40% and inducing heritable metabolic adaptations.

The ‘Reverse Central Dogma’ is supportively corroborated by the Darwin-Ao equation unifying DRS-leading redox information evolutionary dynamics [*dI/dt* = ηΔ*G*σ_τ_ ln (1 + ρ_DRS_/ρ_0_)], as formalized on the base of evolutionary law of natural selection 38. The ‘I’ letter represents system information (bits); η, redox-to-information efficiency (0.15–0.35 empirically); Δ*G*, free energy gradient (kJ/mol); σ_τ_, topological selection pressure (function of spatial code complexity); ρ_DRS_/ρ_0,_ concentrations of DRS normalized to its baseline levels. This was, in microbial evolution, validated by experimentally showing that reducing Δ*G* (of glucose oxidation) from -2870 to -1500 kJ/mol decreases SNP accumulation of *E. coli* by 60%, confirming I ∝ Δ*G* 545 546 547. Conversely, SOD overexpression (to reduce O_₂_^·⁻^ by 90%) lowers antibiotic resistance mutations by 75%, validating I ∝ ln(ρ_DRS_) 545 547 548 549. As for carcinogenic heterogeneity (*H_cr_*), it is found that tumor edge ([O_₂_^·⁻^] = 120 nM) exhibits 3× higher genomic diversity (*H_cr_* = 0.35) than its core region ([O_₂_^·⁻^] = 45 nM, *H* = 0.12), aligning with ρ_DRS_ dependence 550 551 552 553.

DRS-leading thermodynamics and information are integrated to enable free energy principle and Shannon entropy to be revisited. This is owing to traditional sequence entropy ignoring redox-driven epigenetic dynamics. Mathematical modeling shows DNA methylation entropy (Δ*S_m_*) correlates with DRS flux, as Δ*S_m_* = *k_B_* ln(1 + ρ^DRS^/ρ_0_) 554 555 556. While Friston’s model describes homeostasis via free energy minimization, it neglects how DRS converts Δ*G* into heritable information. For instance, *E. coli* enables to use cytochrome c-generated O_₂_^·⁻^ to oxidize OmpR, activating porin genes under starvation, as a direct redox-to-genetic signal 557 558 559.

### The redox-informational analogues existing in societal systems

The economic metabolism is driven by its monetary electron transport chain (mETC). This is exemplified by growth of gross domestic products (GDP, as an analogous to ATP synthesis rate) 560 561, which correlates with currency velocity (≈7.2/yr), possibly mirroring the mitochondrial CoQH• oscillation. Thereby, it is inferable that the 2008-year financial crisis could also be alluded as a ‘metabolic collapse’, in which its M1 multiplier drop (1.7→ 0.8) appears to parallel mitochondrial depolarization (Δψ: -150 → -30 mV), demonstrating a highly conserved energy-information coupling 562 563. The cultural epigenetics can be manifested by social media memory. For instance, viral tweets require >10^⁴^ retweets (as a DRS-similar threshold) to trigger H3K4me3-like algorithmic prioritization, decaying with *t_₁/₂_* = 3–5 days 564 565 566 567. Similarly, if the ‘Like’ function is alluded as a DNMT3A analogue, removing ‘Facebook likes’ reduces meme persistence by 75%, mimicking DNMT3A inhibition 568 569.

### The DRS-leading redox-mediated edge-of-chaos homeostasis selected from life’s origin

The murburn-based GRUT posits that life's homeostasis resides at the homeodynamic ‘edge of chaos’, balancing stochasticity and determinism (as deciphered in **Figure 19**). This is owing to: i) instantaneous stochastic adaptation to generation of DRS (e.g., O_₂_^·⁻^, ^·^OH) by quantum tunnelling (probability 10^⁻⁶^/e-transfer) that induces picosecond-scale conformational flickering in biomolecules, validated by single-molecule FRET (smFRET) 570 571 572 573; ii) long-term deterministic encoding for DRS to covalently modify targets (e.g., by cysteine thiol oxidation) so as to activate MAPK/NF-κB pathways with signal intensities to match redox potentials (*E^°'^*), ensuring thermodynamic precision 574 575 576; iii) membrane-constrained dissipative structures, in which plasma membranes restrict diffusion of DRS (e.g., half-life of O_₂_^·⁻^ < 1 ms), maintaining certain redox gradients and localising stochastic perturbations to prevent systemic collapse 577 578 579 580. Collectively, the steady-state homeostasis is deemed as a redox-governing continuum.

From the murburn-based GRUT perspective, abiogenesis of life originates from UV-driven DRS generation in hydrothermal vents that might have also allowed amino acid peptides and ribonucleotides to be synthesized via formamide radical reactions 271 507 508. In proto-cells, DRS was concentrated and thus compartmentalized in the lipid murzones, fostering polymerization and replication 103 498 579. A dynamic murredox equilibrium to maintain certain redox potential (*Eh*) was finely tuned via a series of anti-redox feedback mechanisms between production of DRS (by murzymes, e.g., NADPH oxidase) and its scavenging (e.g., by SOD). Thus, relevant disease paradigms are inferably exemplified by neurodegeneration and aging, that correlate with murredox dysregulation, implicating mitochondrial DRS disequilibrium. Overall, such redox-unifying principle holds across distinctive scales from cellular to ecosystem levels (in which coral-algal symbiosis relies on murredox signaling via ROS exchanges 581 582), and can be even applied for astrobiological implications (extraterrestrial life detection could prioritize environments conducive to murburn chemistry (similar to that in the Enceladus’ subsurface ocean 583 584).

### Five dimensional codes of DRS-redox dynamics that comprise core mechanisms of GRUT

The murburn-based GRUT of life is inferable to mandate at least five distinct dimensional codes of DRS-redox dynamics. Firstly, ‘quantum code’ could be established and evolved by its tunnelling effects and chaotic dynamics, leading to nonlinear spatiotemporal distributions of DRS. In mitochondrial electron leakage, quantum tunnelling at Complex I's Q-site causes electron escape (probability of 10^⁻³^/ETC), amplified by cytochrome c oxidase allostery to generate respiratory oscillations 585 586 587,588. During superoxide burst phase transitions, neutrophil NOX2 activity triggers a positive feedback (at [H_₂_O_₂_] > 5 μM), with critical thresholds obeying the Ising model phase transitions (critical exponent β ≈ 0.325), revealing quantum on/off switching in immunity 589 590 591 592.

Secondly, ‘topological space code’ could be established originally from the membrane fractal architecture to signal-focusing membrane fractals (*D*≈2.32) to optimize DRS-redox signaling efficiency, driving self-organization and self-regulation. This is exemplified by lipid raft-mediated signal focusing on sphingomyelin microdomains (*d* ≈20 nm), in which NOX4 is enriched, tripling O_₂_^·⁻^ production and correlating with cancer stem cell drug resistance (r=0.78) 593 594 595. Interestingly, membrane curvature-modulated redox waves existing in mitochondrial cristae (*r*≈ 30 nm) enable to enhance CoQ10 diffusion, boosting DRS generation by 50% and driving ATP synthesis (with a hill coefficient *n*=2.1) 596 597 598 599 600.

Thirdly, ‘metabolic code’ may also be established and improved through compartmentalized redox potentials and chaos. This is manifested by dynamically (re)partitioning and/or (re)positioning of reductive equivalents (e.g., NADPH, GSH) across subcellular compartments (i.e., murzone). Such compartmentalized redox gradients of cytosol (*E^°'^*≈ -320 mV) and mitochondrial matrix (*E^°'^*≈-360 mV) are maintained via malate-aspartate shuttles, but upon its collapse (lipid peroxides > 50 μM), ferroptosis is triggered 601 602. Chaotic metabolic oscillations are embodied by glycolytic flux-ROS interactions exhibiting Lorenz attractor dynamics (Lyapunov exponent λ≈0.9), with cancer Warburg effects representing chaos-driven steady-state shifts 603 604 605 606.

Fourthly, ‘time code’ serves as a coding rule mandated by evolutionary timing with multi-scale rhythms. Here, it is crucial important to note that DRS redox dynamics can encode discrepant temporal programmes across scales. i) Ultrafast (ns-ms): mitochondrial O_₂_^·⁻^ bursts (≈10^⁶^/s) synchronize with electron quantum tunnelling events (with a phase error <1%) 517 607 608. Ii) Physiological scale (s-hr): NAD^⁺^/NADH oscillations couple with H_₂_O_₂_ peaks (at Zeitgeber time 12) to entrain circadian clocks by Sirt1-BMAL1/CLOCK pathways 609 610 611 612. Iii) Evolutionary scale (days-eons): The catalase gene duplications mirror the Earth's oxygenation history (*R^²^*=0.93), demonstrating redox adaptation over geological time 613 614 615 616.

Fifthly, ‘murzyme code’ is deciphered at five distinct layers (in the legend of **Figure 19**), predominantly serving DRS-driven catalytic reactors and energy transduction. Hemeproteins, flavoproteins and other murnburn-relevant factors were defined as ‘murzymes’ 516 579, forming DRS-driven ‘nuclear reactor’-like systems. Just by the electron transfer, cytochrome c oxidase achieves >90% energy efficiency via the ‘Fe-S’-cluster quantum tunnelling, surpassing classical enzyme limits (≈40%) 580. In the mitochondrial O_₂_^·⁻^ burst-moded intermediate generation, flavoproteins (e.g., NADPH oxidase) produce O_₂_^·⁻^ possibly at 10^⁶^/s rates, akin to ‘nuclear fission chain’ reactions, enabling rapid stress response 592 617. The Fe-S clusters at hydrothermal vents catalyze CO_₂_ to yield acetate, with lipid vesicles forming proton gradients 37 271 507 618. This validates ‘metabolism-first’ that reverses the Central Dogma models in DRS-driven origin of life. 

Redox reshaping of Cell Theory occurs by DRS-driven evolution via membrane topogenetics. This is supported by the fact that archaeal membrane adaptation is acquired by the sulfur ester bonds in thermophilic archaea that reduce GSH consumption by 60%, enabling survival in extreme environments 619 620 621. Mitochondrial ancestors released O_₂_^·⁻^, leading to 3-fold activation of the eukaryotic host NF-κB pathways, so to drive endosymbiosis 622 623. Importantly, redox programming of cognitive function also couples with DRS dynamics. Synaptic quantum coherence is networked by O_₂_^·⁻^ bursts (≈40 Hz) from hippocampal CA1 neurons, so to enhancing NMDA receptor opening (by 70%) and long-term potentiation (LTP) 624 625. Consciousness at the edge of chaos is obtained by prefrontal H_₂_O_₂_ fluctuations (15%) to maintain cortical criticality (critical exponent β≈0.5) via GABAergic regulation, underpinning creative thoughts 626 627 628. Thereby, DRS-driven programming systems to bioenergetic powering (thermogenesis), coherence, homeostasis, electro-mechanic mobility, and sensing to signaling responses (PCHEMS) are precision-selected by accurately integrating redox biochemistry, electrophysiology and quantum biology from life’s origin (by biological intelligence) to cognitive emergence and evolution.

### Several key terminologies about murburn concept

The murburn (from mur + burn) concept posits that DRS including all transient reactive molecules, unbound ions and radicals with proton quanta and/or radiations, generated via mild, unrestricted redox ‘burning’ reactions, drive these processes through stochastic, even membrane-independent, interactions and modifications. This paradigm shift necessitates a critical re-interpretation of redox biology’s foundational principles 497, because anomalies such as DRS effects, nonlinear thermodynamics and kinetics, and system-wide redox buffering remain inadequately explained, before erstwhile redox reactions (particularly central to energy transduction and homeostasis) had long been interpreted by classic enzyme-substrate specificity and compartmentalized electron transport chains.

As redox mediators beyond canonical enzymology, murzymes are referred to as biomolecules (proteins, lipids, or nucleic acids) and other factors involved in the murburn reactions and interactions that facilitate DRS generation, modulation, utilization or termination, along with no formation of stable substrate complexes 517 579 580. Its three key features include, i) DRS catalysis: hemoproteins like hemoglobin act as murzymes by releasing Fe^²⁺^/Fe^³⁺^ ions to catalyze Fenton reactions 571 629; ii) Redox buffering: thioredoxin systems regulate •NO levels via thiol-disulfide exchanges, independent of its active-site binding 42 630 631; iii) Evolutionary implications: primordial murzymes could have preceded those highly specialized enzymes, functioning as versatile redox "hubs" in prebiotic environments 499 504 631.

The spatial dynamics of redox activity driven by DRS is manifested by murzones, as specified microdomains in which DRS transiently reach to the peak of its concentration gradients, enabling redox (burning) reactions in spite of the bulk-phase instability. This is exemplified by, i) Membrane interfaces: the lipid bilayers’ hydrophobic regions stabilize radicals (e.g., lipid peroxidation chains) 570 579 632; ii) Cellular and macromolecular crowding in the cytosol to enhance radical recombination rates 633 634 635; iii) Photochemical activation: chloroplast thylakoids utilize light to generate O_₂_^·⁻^ in the murzones, driving photosynthetic electron transport 579 600 636 637.

The systems-wide homeostasis is finely tuned by murredox, denoting the integration of all murburn processes into global redox buffering and signaling responsive networks. It is inferable to explain those phenomena, such as: i) Stress resilience: glutathione’s role in scavenging DRS is amplified by murzonal •H_₂_O_₂_ diffusion 498; ii) Metabolic flexibility: cancer cellular Warburg effect may stem from murredox adaptations to hypoxic environments 606 638; iii) Cross-kingdom’s conservation: similar redox programming patterns in all existing extremophiles and eukaryotes demonstrate universal murredox principles 499 504.

### Challenges and future directions in the murburn-based GRUT

A great challenge of technological frontiers is to develop real-time single-DRS imaging. Recently, plasmonic SERS (surface-enhanced Raman scattering) nanoprobes achieve a high sensitivity ( of ~10^⁻¹⁸^ M) for real-time O_₂_^·⁻^ tracking in the mitochondrial cristae 639 640. The decryption of topological code is also another huge challenge. Graph neural networks (GNNs) are subjected to mapping of all those protein-folding trajectories to redox potential landscapes with self-organized building blocks, predicting topovectorial spatial coding rules.

For philosophical implications, this murburn-based GRUT has transcended the Cartesian dualism, proposing ‘*I oxidize, therefore I am*’—as a monist view where all intelligence, consciousness, society, and life itself (as shown in **Figure 19**) are manifestations of self-referential redox-information flows in real essences. Thereby, it is inferable that each of life forms appears to be just a masterpiece of natural selection, as perfectly written by ‘DRS-lettered Redox settings’ as a universal *lingua franca* accounting for all live beings. In such redox-driven life’ language system, DRS are the most active, powerful, primitive, fundamental, and radical 'elementary letters and/or core keywords', involved ubiquitously in a vast variety of stochastic (short-term) and/or deterministic (long-term) programming by multidimensional spatiotemporal operations to exert their leading physiological functions of life (e.g., PCHEMS). 

In summary on the GRUT, each life is selectively established and grown as a continuum of redox-programmed information topologies, governed by universal principles from the prebiotic chemistry to socioeconomic dynamics. By replacing the Central Dogma’s linear hierarchy with such a redox-informational framework, the murburn-based GRUT provides a predictive power across scales—from nature-designing synthetic cells to present-day modeling economic resilience. As we enter the era of redox omics and topological bioengineering, this paradigm promises to unify discrete fractured (e.g., evolutional biological) landscapes of life sciences (to all relevance’s), much as the standard model unified fundamental physics. Altogether, GRUT enables to exist as a natural evolutionary selective, fundamental, constitutional principle accounting for all cellular life forms.

## AUTHOR CONTRIBUTION

Y.Z. designed this work within several novel conceptualized terms, analyzed all relevant data, prepared all figures with cartoons, and wrote and revised the paper. M.W. collected and analyzed the data measured by transcriptome, proteome, and metabolome, and prepared the relevant figures with networks, whereas Y(-ping) Z. had done bioinformatics analysis and made the phylogenetic tree with structural cartoons. C.L. and W.S. wrote the section about co-evolutionary histories of life with its ambient redox environments and made related figures. P.A. has provided an invaluable discussion about theoretical physics with its Darwinian evolution law and network dynamics. H.T. wrote and revised the section about neuroscience. X.C. and Z.Z collected relevant bioinformatics data, edited this paper, and polished the English language. Lastly, all these co-authors have read and agreed to the published version of the manuscript.

## CONFLICT OF INTEREST

The authors declare no conflict of interest.

## SUPPLEMENTAL MATERIAL 

Click here for supplemental data file.

All supplemental data for this article are available online at https://www.cell-stress.com/researcharticles/2025a-zhang-cell-stress/
