# Supplementary material for: Nrf1 acts as a highly-conserved determinon for maintaining robust redox homeostasis in the eco-evo-devo process of life histories
Source: Cell Stress. 2025 Jul 7;9:65–142. doi: 10.15698/cst2025.07.306 (PMC12285602; doi:10.15698/cst2025.07.306)
Supplement: Supplementary file 1 [file ces-09-065-s01.pdf]

Supplemental Material

Nrf1 acts as a highly-conserved determinon for maintaining robust redox homeostasis in the eco-evo-devo process of life histories

Yiguo Zhang[1,2,*], Xi Chen[1], Meng Wang[1], Yuping Zhu[1,3], Wei Shi[4,5,6], Chao Li[4,5,6], Zhengwen Zhang[7], Hiroaki Taniguchi[8,9] and Ping Ao[10]

[1] The Laboratory of Cell Biochemistry and Topogenetic Regulation, College of Bioengineering and Faculty of Medical Sciences, Chongqing University, No. 174 Shazheng Street, Shapingba District, Chongqing 400044, China. [2] School of Life and Health Sciences, Fuyao University of Science and Technology, No. 104 Wisdom Avenue, Nanyu Town, Minhou County High-Tech District, Fuzhou 350109, Fujian, China. [3] School of Basic Medicine, Guizhou Medical University, No. 6 Ankang Avenue, GUI 'an New District, Guizhou 561113, China. [4] State Key Laboratory of Oil and Gas Reservoir Geology and Exploitation & Institute of Sedimentary Geology, Chengdu University of Technology, Chengdu 610059, China. [5] Key Laboratory of Deep-time Geography and Environment Reconstruction and Applications of Ministry of Natural Resources, Chengdu University of Technology, Chengdu 610059, China. [6] International Center for Sedimentary Geochemistry and Biogeochemistry Research, Chengdu University of Technology, Chengdu 610059, China. [7] Laboratory of Neuroscience, Institute of Cognitive Neuroscience and School of Pharmacy, University College London, 29-39 Brunswick Square, London WC1N 1AX, England, United Kingdom. [8] Department of Experimental Embryology, Institute of Genetics and Animal Biotechnology, Polish Academy of Sciences, 05-552 Jastrzebiec, Poland. [9] African Genome Center, Mohammed VI Polytechnic University (UM6P), Ben Guerir 43150, Morocco. [10] College of Biomedical Engineering, Sichuan University, Chengdu, Sichuan 610044, China.

⋆ Corresponding Author:
Yiguo Zhang, E-mail: eaglezhang@fyust.edu.cn or yiguozhang@cqu.edu.cn

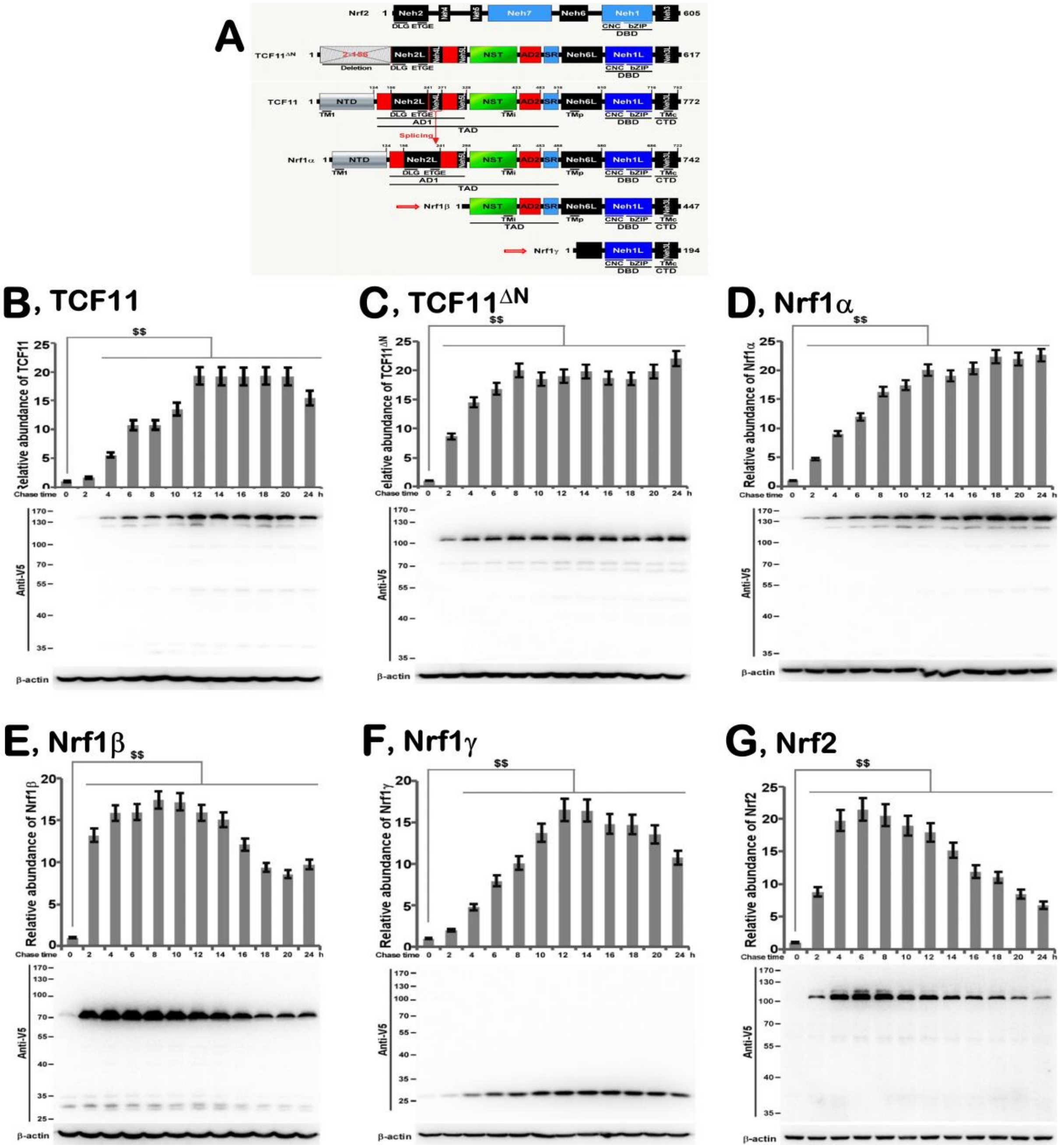

Supplemental FIGURE S1 ● Distinct isoforms of Nrf1/TCF11, along with and Nrf2, which were inducibly expressed in the tetracycline-inducible HEK293 cell system, that had been treated with tetracycline for indicated lengths of time.

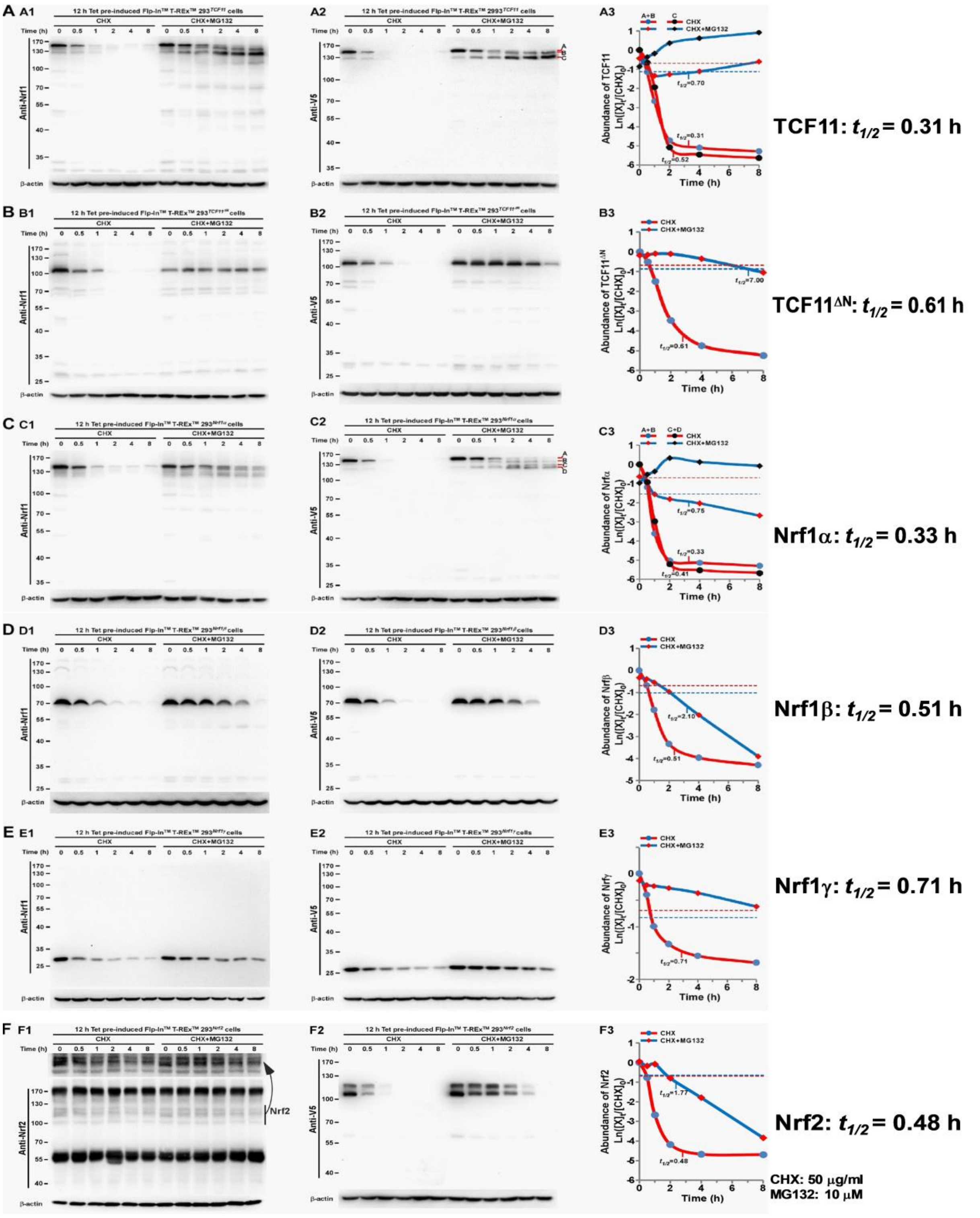

Supplemental FIGURE S2 ● The protein stability of distinct Nrf1/TCF11 isoforms along with and Nrf2, which were estimated by their half-lives in the tetracycline-inducible HEK293 cell system that was pretreated with tetracycline for12 h and then treated with CHX alone or plus MG132 for distinct lengths of chase time. The resulting data were shown graphically before calculating their half-lives.

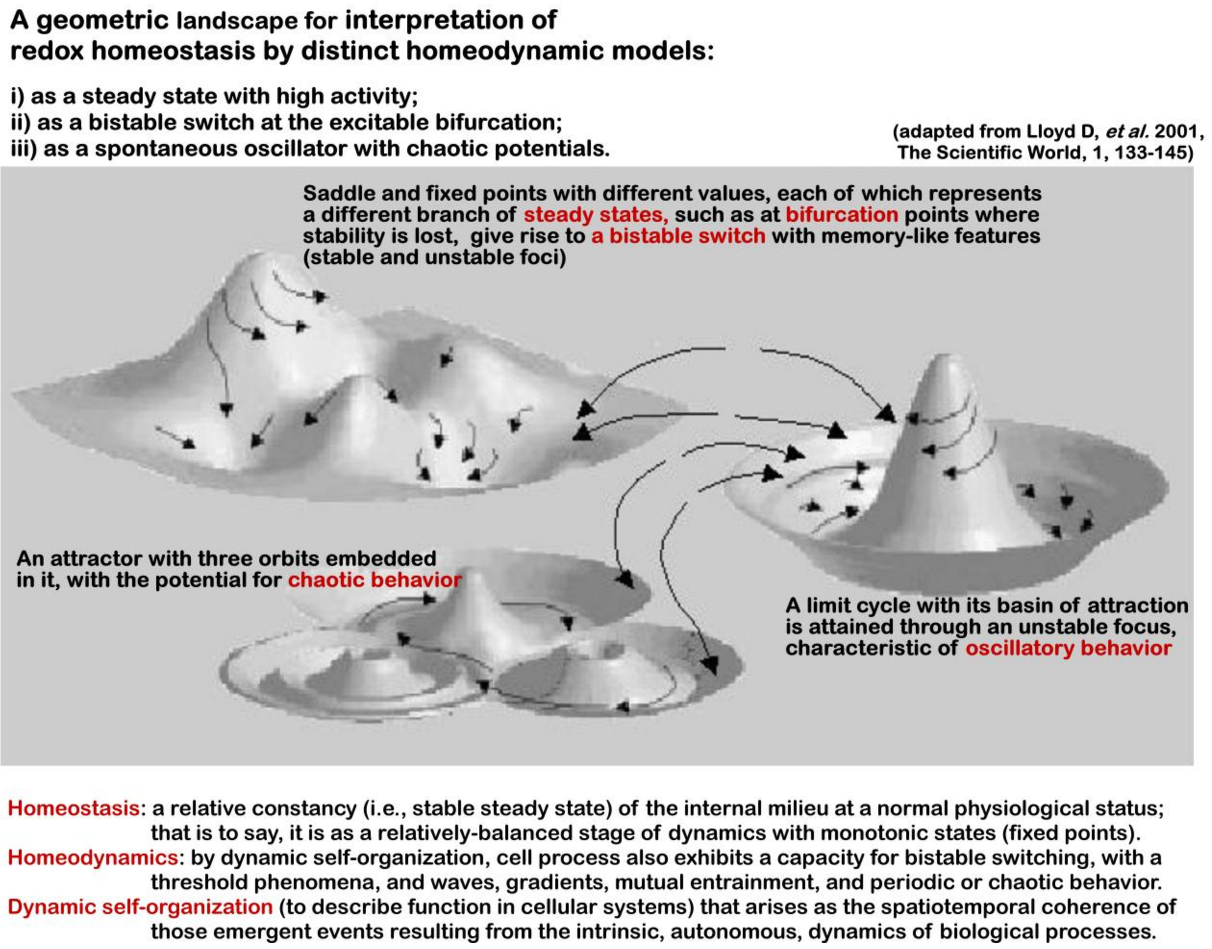

Supplemental FIGURE S3 ● A geometric landscape for interpretation of redox homeostasis by distinct homeodynamic models, which was adapted from Lloyd D, et al 2001 [182].

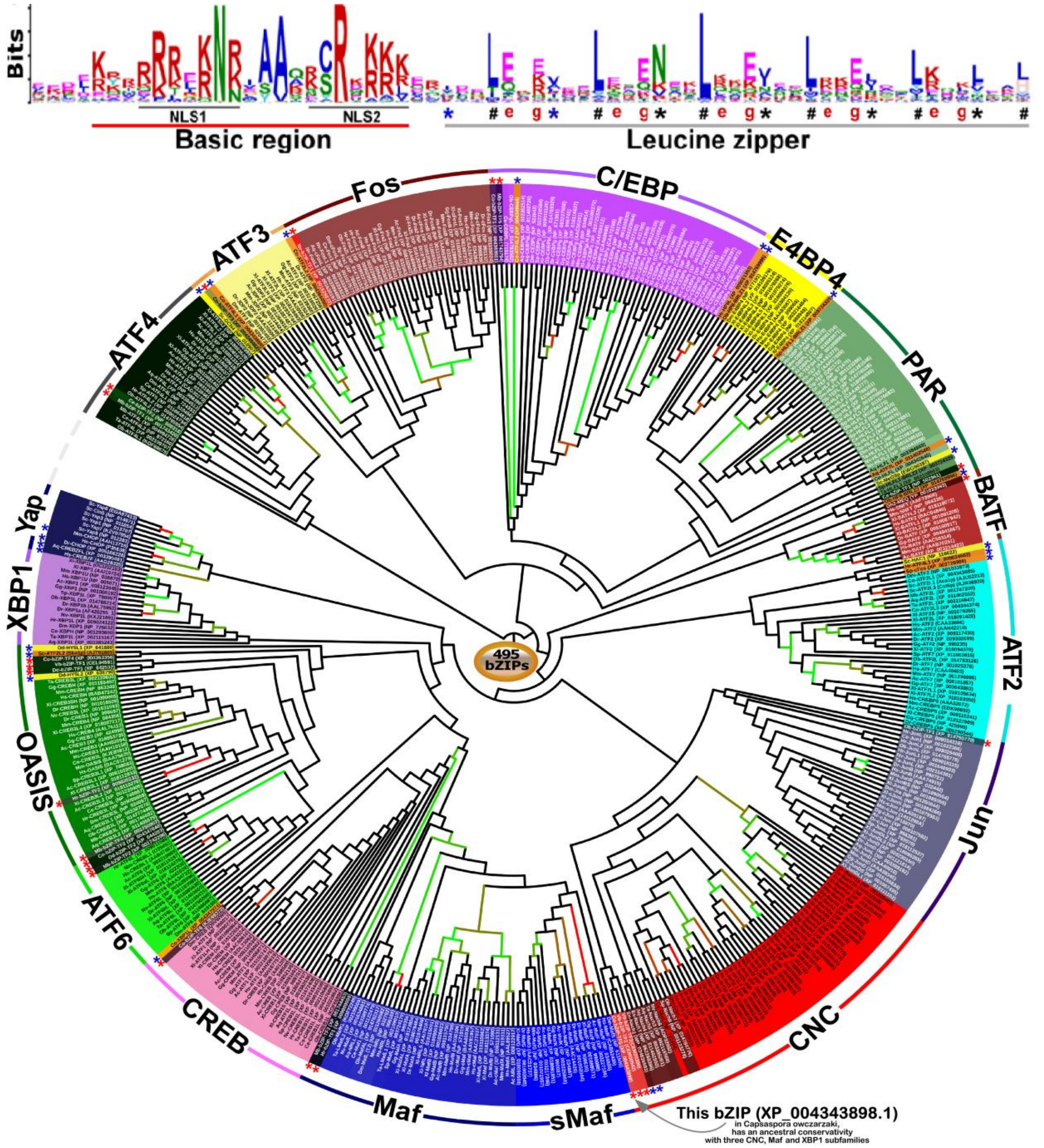

Supplemental FIGURE S4 ● The neighbor-joining phylogenetic tree of 495 bZIP factors selected from distinct species of life and viruses.

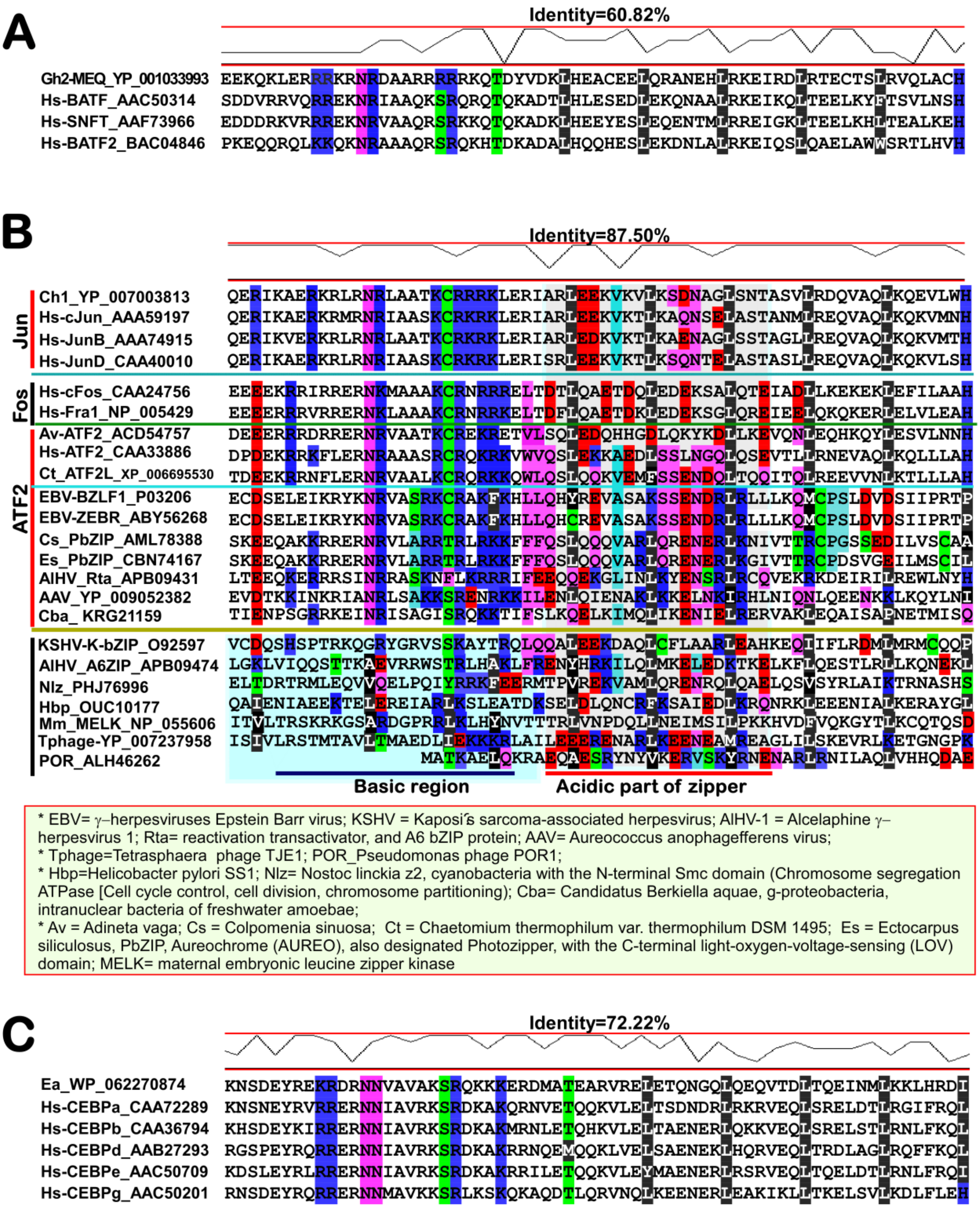

Supplemental FIGURE S5 ● Alignment of multiple amino acid sequences covering the core DNA-binding domains of those early emerged bZIP factors, along with viral and bacterial bZIP factors.

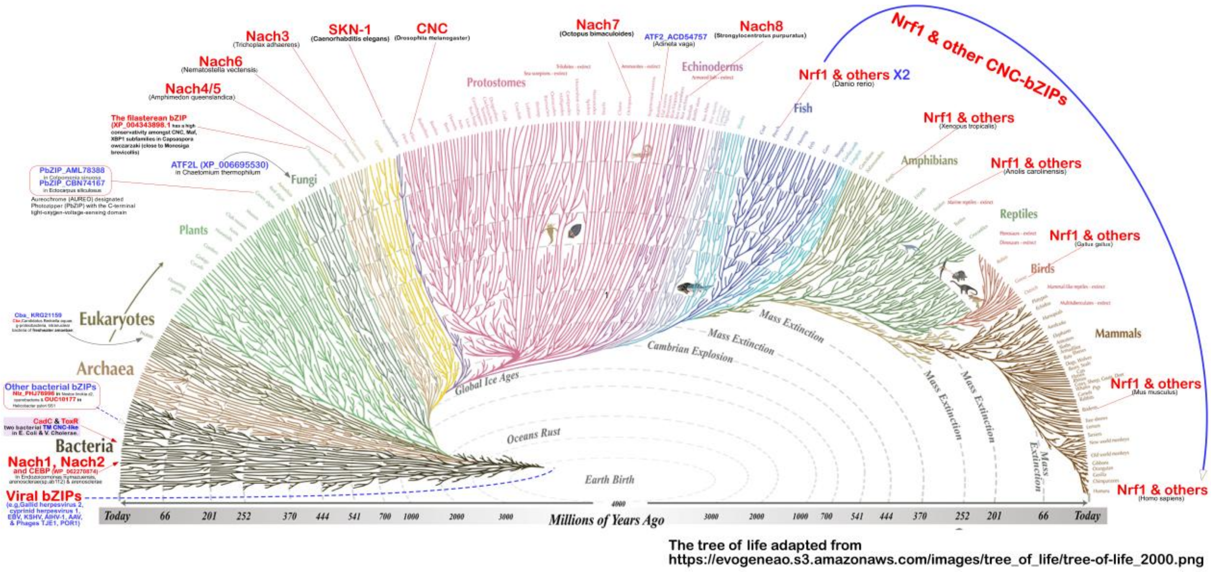

Supplemental FIGURE S6 ● All indicated CNC-bZIP factors and relevant homologues are marked on the evolutionary tree of life (adapted from online as indicated).

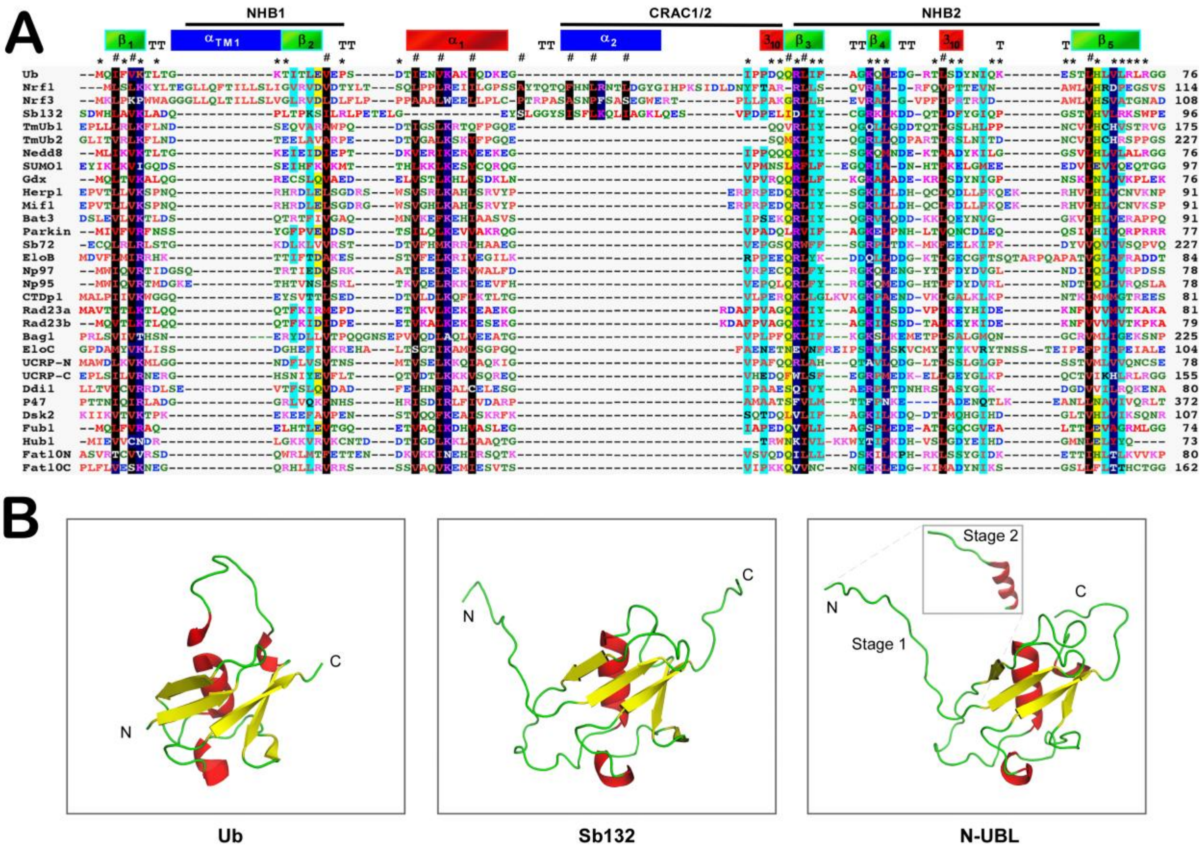

Supplemental FIGURE S7 ● Alignment of amino acid sequences from the N-terminal domains of Nrf1 and Nrf3, with all those indicated ubiquitin-like (UBL) proteins or UBL-containing proteins (A), along with structural modeling of N-UBL in Nrf1 being built (B).

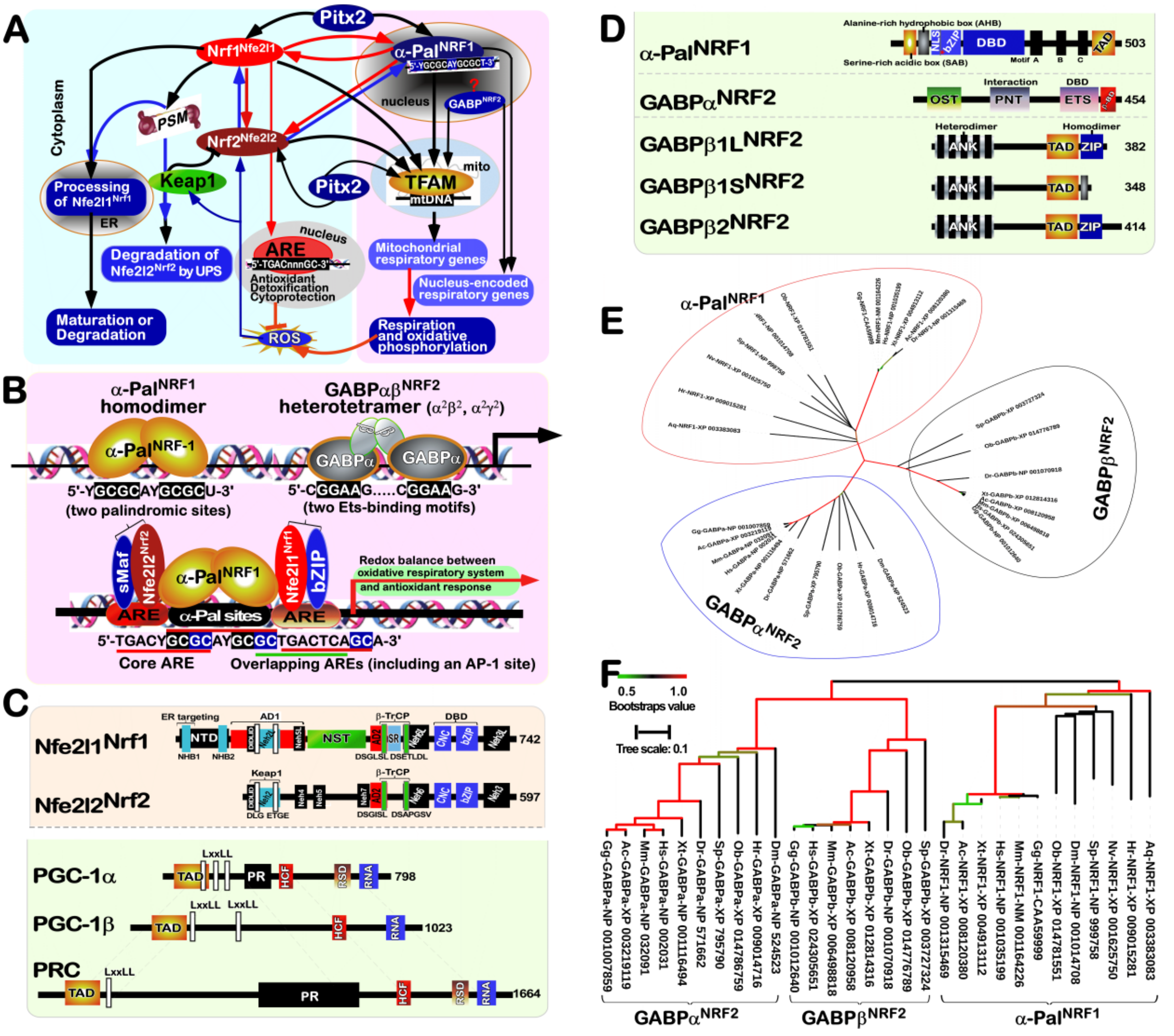

Supplemental FIGURE S8 ● Antioxidant Nrf1/2 are totally different from nuclear respiratory factors (also abbreviated often as NRF-1/2). (A) A proposed model to give a better understanding of inter-regulation between two major antioxidant factors Nrf1Nfe2l1 and Nrf2Nfe2l2 with additional two nuclear respiratory factors (also designated as αPal$^{\mathrm{NRF1}}$ and GABP$^{\mathrm{NRF2}}$, herein) in coordinately governing the mitochondrial biogenesis and functional homeostasis. (B) Schematic representation of directly DNA-binding of αPal$^{\mathrm{NRF1}}$ and GABP$^{\mathrm{NRF2}}$, alongside with Nrf1$^{\mathrm{Nfe2l1}}$ and Nrf2$^{\mathrm{Nfe2l2}}$, to their respective consensus sequences. (C-D) Structural comparison of Nrf1$^{\mathrm{Nfe2l1}}$, Nrf2$^{\mathrm{Nfe2l2}}$, αPal$^{\mathrm{NRF1}}$, GABP$^{\mathrm{NRF2}}$, and their co-factors PGC-1α, PGC-1β and PRC, all with distinct functional domains as indicated. (E, F) Two types of the neighbor-joining phylogenetic tree, revealing no homology amongst αPal$^{\mathrm{NRF1}}$, GABPα$^{\mathrm{NRF2}}$ and GABPβ$^{\mathrm{NRF2}}$.

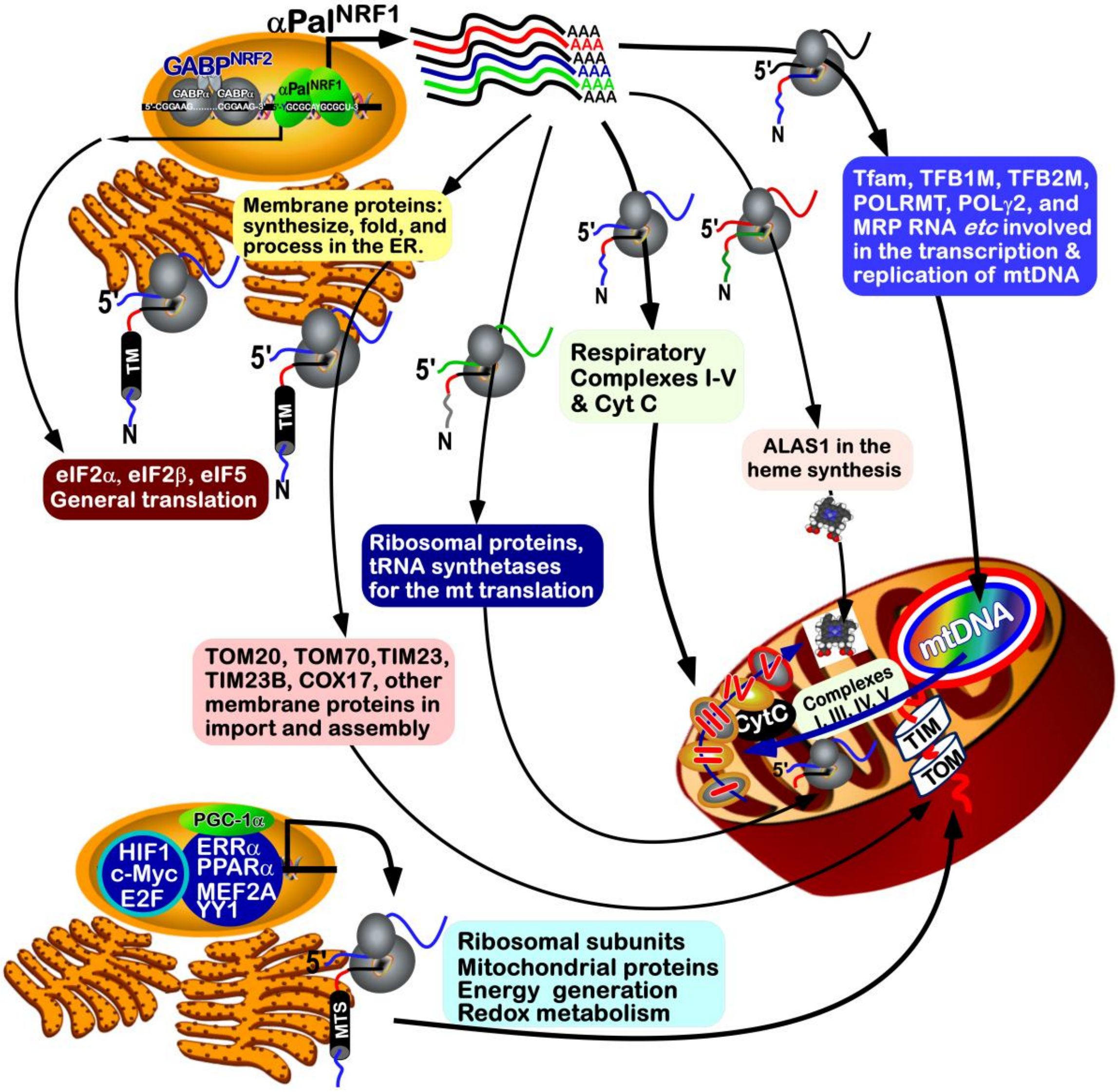

Supplemental FIGURE S9 ● A proposed model for a better understanding of **α**Pal^NRF1 acting as a major nuclear respiratory factor, which making multiple cross-talks from the nucleus (and endoplasmic reticulum) to mitochondria, through several distinct signaling to target gene regulatory networks.

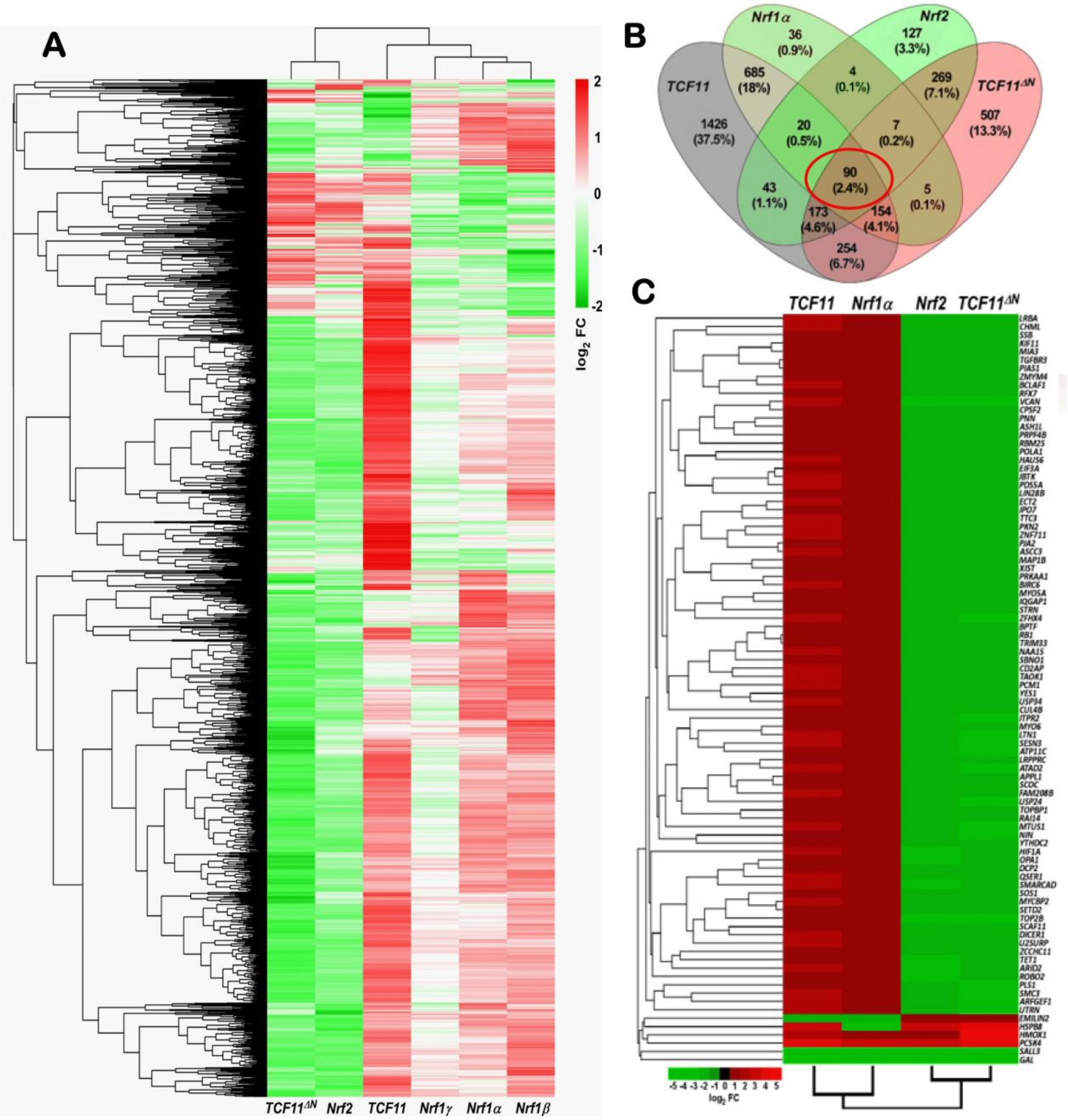

Supplemental FIGURE S10 ● **Transcriptomic analysis of tetracycline-inducible HEK293 cells expressing each of indicated CNC-bZIP factors.** Of note, Nrf1 and TCF11 have similar target gene regulatory roles, that are completely distinctive or even oppositely from those regulated by Nrf2 (but almost resembles TCF11$^{\Delta N}$).

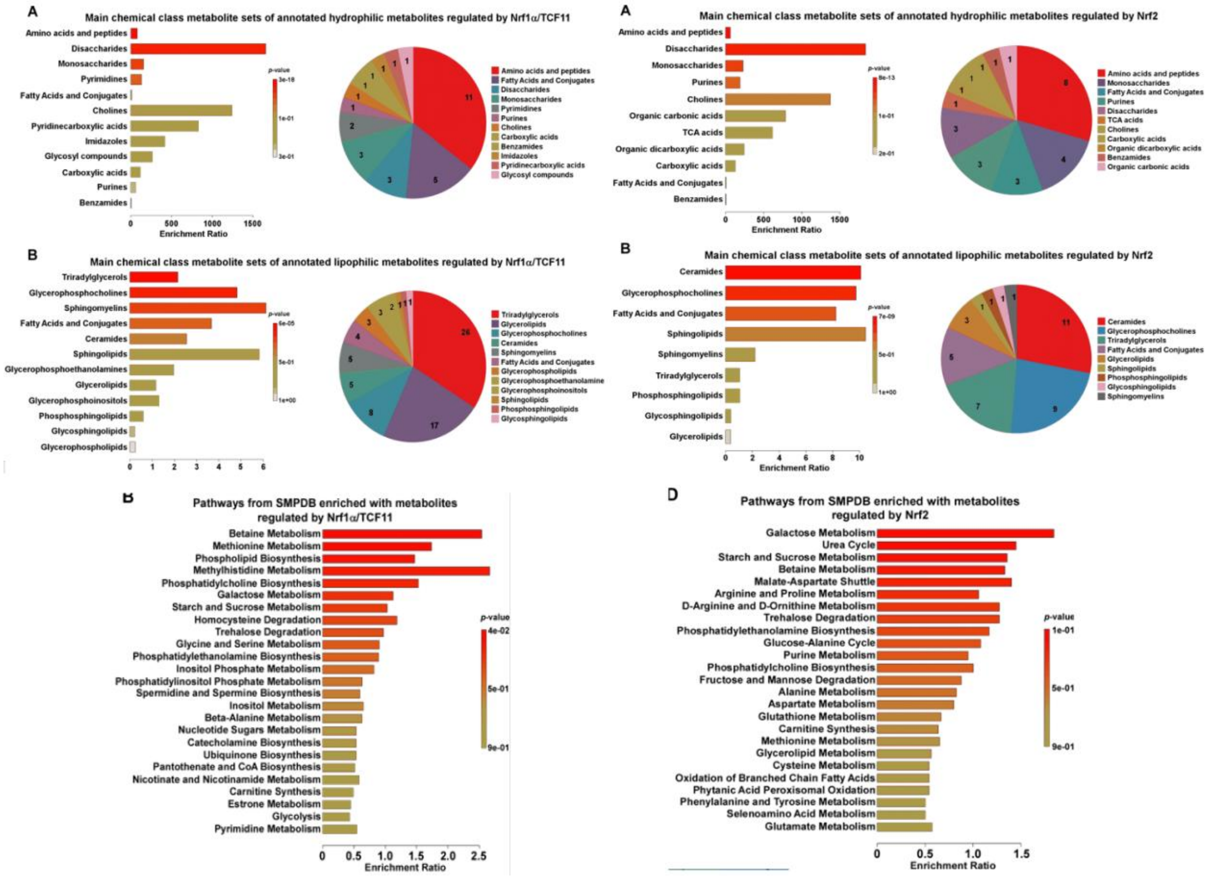

Supplemental FIGURE S11 ● Metabolome analysis of distinctions between Nrf1α/TCF11 and Nrf2.

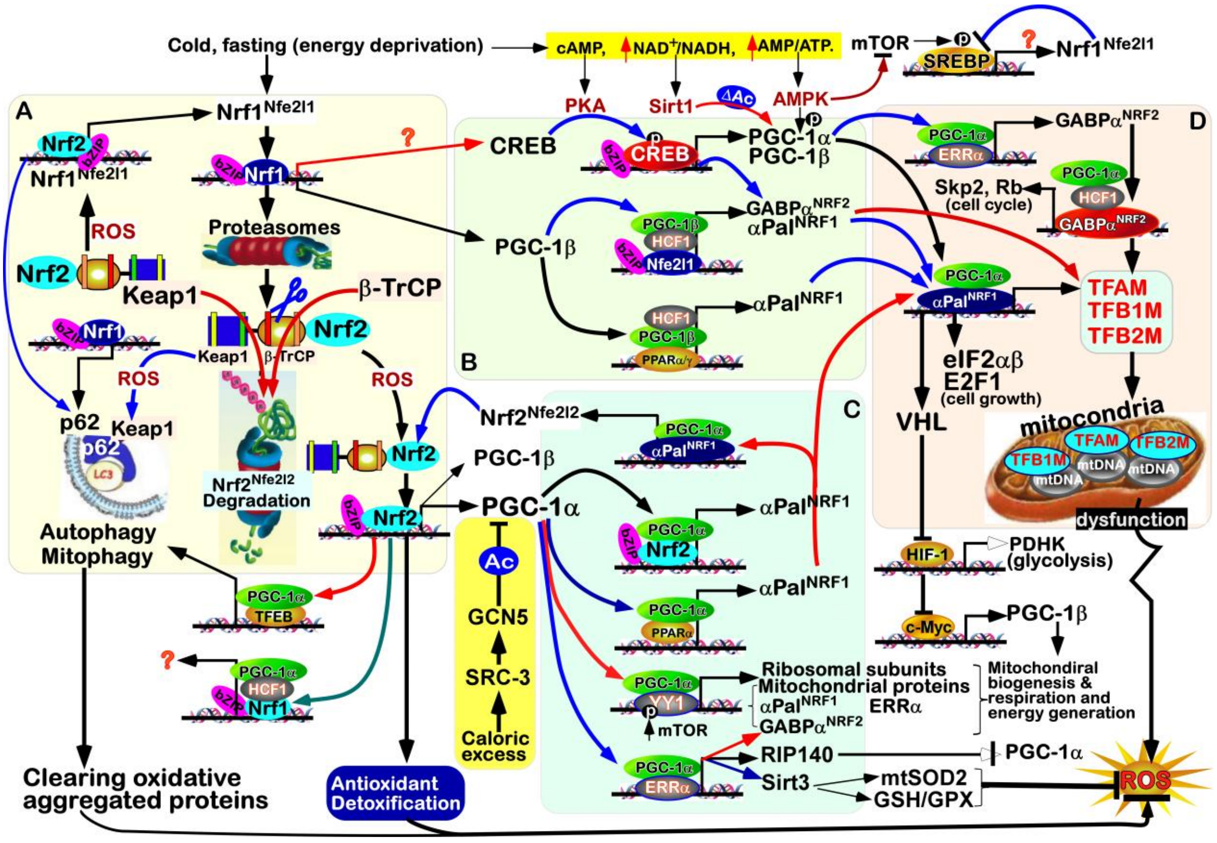

Supplemental FIGURE S12 ● A proposed model to provide an understanding of differentiated yet integrated roles of Nrf1^{Nfe2l1}, Nrf2^{Nfe2l2} and **α**Pal^{NRF1} in coordinately mediating multi-hierarchical molecular-cellular signaling to target gene regulatory networks in order to tightly govern cellular (redox and energy metabolism) homeostasis, as well as mitochondrial homeostasis, under the strict quality control.

The following work had been suspected of being fraudulent, adapted from PubPeer at https://pubpeer.com/publications/34FAE52A483C74E4D42770D878A999

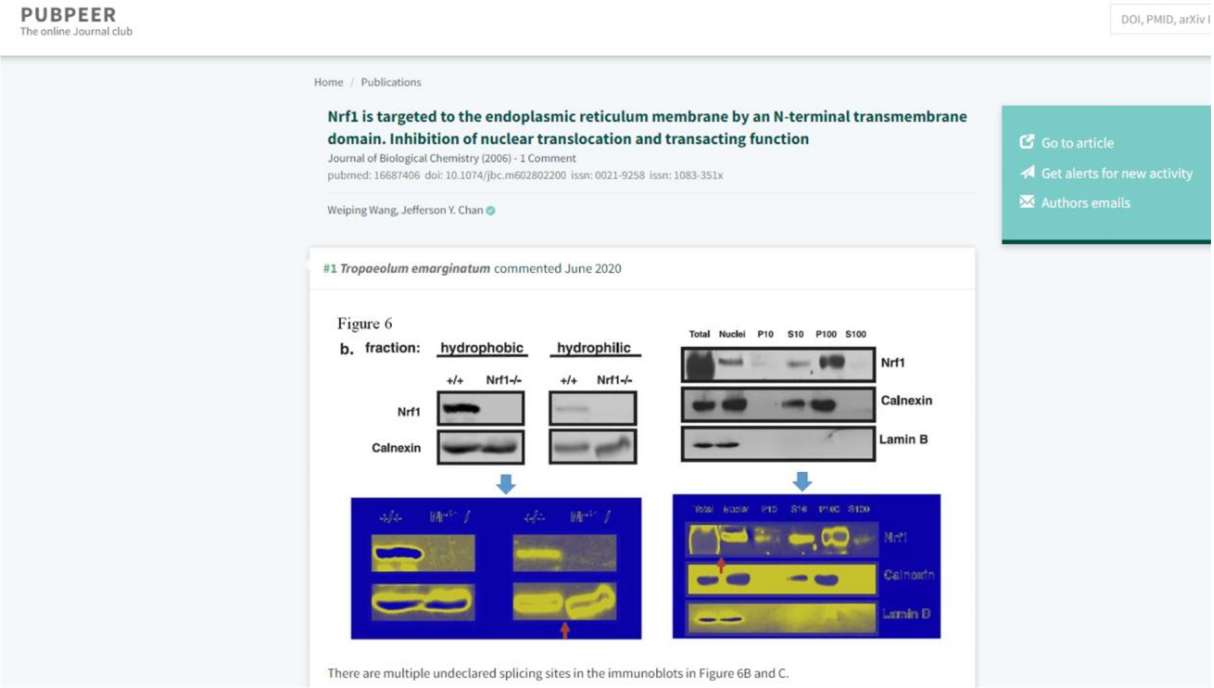

Supplemental FIGURE S13 ● **In the past near 20 years, this group has almost no more continuing studies except for this misleading publication having been suspected by the PubPeer, which was wrongly designed for the tagged protein, resulting in a misfolded topology of Nrf1 across the membranes, as accompanied by several misleading interpretations.** For example, i) Tunicamycin (TU)-leading decreases in molecular weights of Nrf1 were misunderstood to be proteolytically processed in a fashion similar to that of ATF6; ii) Nrf1 was viewed to be repressed directly by Keap1 in another fashion similar to that of Nrf2. This is attributed to the routine CO-IP assayed in the total lysates with broken membranes, leading to a false positive interaction of Keap1 with Nrf1. All these have been later experimented to be wrong. Correctly, TU enables to block the N-linked glycosylation of Nrf1 insomuch as to yield a non-glycosylated form, but not be processed by TU-induced proteases through a similar mechanism accounting for the proteolytic processing of ATF6 or SREBP. In fact, the Neh2-like domain of Nrf1 is positioned in the ER lumen and only when it is retro-translocated out of the ER, it can gain access to the cytosolic Keap1. Thus, in total cell lysates with broken membranes, Nrf1 is easy to interact with Keap1 upon lacking the membrane barrier. In addition, there should also exist another subtle 'grey' story being hidden behind this publication, which has been not uncovered heretofore to anyone except for the authors.

REFERENCES

182. Lloyd D, Aon MA, Cortassa S (**2001**). Why homeodynamics, not homeostasis? **ScientificWorldJournal** 1: 133-145. doi: 10.1100/tsw.2001.20
